# Supplementary material for: Attenuated growth factor signaling during cell death initiation sensitizes membranes towards peroxidation
Source: Nat Commun. 2025 Feb 25;16:1774. doi: 10.1038/s41467-025-56711-2 (PMC11861335; doi:10.1038/s41467-025-56711-2)
Supplement: Supplementary file 1 — Supplementary Information [file 41467_2025_56711_MOESM1_ESM.pdf]

Supplementary information

Attenuated growth factor signaling during cell death initiation sensitizes membranes towards peroxidation

André Gollowitzer[1 §], Helmut Pein[2 §], Zhigang Rao[1 #], Lorenz Waltl[1 #], Leonhard Bereuter[1,3 #], Konstantin Loeser[2], Tobias Meyer[4,5], Vajiheh Jafari[2], Finja Witt[1], René Winkler[6,7], Fengting Su[1,3], Silke Große[8], Maria Thürmer[2], Julia Grander[1], Madlen Hotze[9], Sönke Harder[10], Lilia Espada[11], Alexander Magnutzki[12], Ronald Gstir[12], Christina Weinigel[13], Silke Rummler[13], Günther Bonn[12], Johanna Pachmayr[14], Maria Ermolaeva[11], Takeshi Harayama[15], Hartmut Schlüter[10], Christian Kosan[6], Regine Heller[8], Kathrin Thedieck[16-19], Michael Schmitt[4], Takao Shimizu[20,21], Jürgen Popp[4,5], Hideo Shindou[22,23], Marcel Kwiatkowski[9] & Andreas Koeberle[1,2,3*]

[1]Michael Popp Institute and Center for Molecular Biosciences Innsbruck (CMBI), University of Innsbruck, 6020 Innsbruck, Austria. [2]Chair of Pharmaceutical/Medicinal Chemistry, Institute of Pharmacy, Friedrich-Schiller-University Jena, 07743 Jena, Germany. [3]Institute of Pharmaceutical Sciences and Excellence Field BioHealth, University of Graz, Graz, Austria. [4]Institute of Physical Chemistry and Abbe Center of Photonics, Friedrich-Schiller-University Jena, 07743 Jena, Germany. [5]Leibniz Institute of Photonic Technology Jena e.V., Member of Leibniz Health Technology, 07745 Jena, Germany. [6]Department of Biochemistry, Center for Molecular Biomedicine (CMB), Friedrich-Schiller-University Jena, 07745 Jena, Germany. [7]Josep Carreras Leukaemia Research Institute (IJC), Campus Can Ruti, 08916 Badalona, Spain. [8]Institute of Molecular Cell Biology, Center for Molecular Biomedicine (CMB), Jena University Hospital, 07745 Jena, Germany. [9]Institute of Biochemistry and Center for Molecular Biosciences Innsbruck, University of Innsbruck, 6020 Innsbruck, Austria. [10]Institute of Clinical Chemistry and Laboratory Medicine, Section Mass Spectrometry and Proteomics, University Medical Center Hamburg-Eppendorf, 20246 Hamburg, Germany. [11]Leibniz Institute on Aging - Fritz Lipmann Institute (FLI), 07745, Jena, Germany. [12]ADSI-Austrian Drug Screening Institute, University of Innsbruck, 6020 Innsbruck, Austria. [13]Institute of Transfusion Medicine, University Hospital Jena, 07747 Jena, Germany. [14]Institute of Pharmacy, Paracelsus Medical University, 5020 Salzburg, Austria. [15]Institut de Pharmacologie Moléculaire et Cellulaire, Université Côte d'Azur - CNRS UMR7275 - Inserm U1323, 06560 Valbonne, France. [16]Department Metabolism, Senescence and Autophagy, Research Center One Health Ruhr, University Alliance Ruhr & University Hospital Essen, University Duisburg-Essen, Essen, Germany, [17]Freiburg Materials Research Center FMF, Albert-Ludwigs-University of Freiburg, 79104 Freiburg, Germany. [18]Laboratory of Pediatrics, Section Systems Medicine of Metabolism and Signaling, University of Groningen, University Medical Center Groningen, 9713 AV, Groningen, The Netherlands. [19]Department of Neuroscience, School of Medicine and Health Sciences, Carl von Ossietzky University Oldenburg, 26129 Oldenburg, Germany. [20]Department of Lipid Signaling, National Center for Global Health and Medicine, Shinjuku-ku, Tokyo, Japan. [21]Institute of Microbial Chemistry, Shinagawa-ku, Tokyo, Japan. [22]Department of Lipid Life Science, National Center for Global Health and Medicine, Shinjuku-ku, Tokyo, Japan. [23]Department of Medical Lipid Science, Graduate School of Medicine, The University of Tokyo, Bunkyo-ku, Tokyo, Japan.

Supplementary Figures

Supplementary Fig. 1	Fibroblast morphology, numbers, and viability under cytotoxic conditions
Supplementary Fig. 2	PARP cleavage, PS externalization, cell viability, and membrane intactness
Supplementary Fig. 3	Cytotoxic stress-induced changes in phospholipid content and composition
Supplementary Fig. 4	Phospholipid species regulated by cytotoxic stress
Supplementary Fig. 5	Phospholipid fatty acid profile of stressed cells
Supplementary Fig. 6	Cytotoxic stress-induced changes in the PC fatty acid distribution
Supplementary Fig. 7	Phospholipid unsaturation under cytotoxic stress
Supplementary Fig. 8	Subcellular fractionation of VAL-treated fibroblasts
Supplementary Fig. 9	Dynamics of cell density and cell cycle progression and effect of medium exchange
Supplementary Fig. 10	Proportion of PUFA-PC in cancer- and non-cancer cells under cytotoxic stress
Supplementary Fig. 11	Knockdown of ACC1 in fibroblasts
Supplementary Fig. 12	Contribution of ACC to PUFA metabolism under cytotoxic stress
Supplementary Fig. 13	The stress-induced increase in PUFA-PC ratios depends on MUFA availability and LPLAT12
Supplementary Fig. 14	Regulation of key enzymes in de novo fatty acid biosynthesis
Supplementary Fig. 15	GO term enrichment analysis of biological processes
Supplementary Fig. 16	Stress-induced changes in the availability of proteins related to glycolysis, gluconeogenesis, and the TCA cycle
Supplementary Fig. 17	Stress-induced changes in the availability of proteins related to the pentose phosphate cycle
Supplementary Fig. 18	Stress-induced changes in the availability of proteins related to fatty acid metabolism
Supplementary Fig. 19	Stress-induced changes in the availability of lysophospholipids and proteins related to (phospho-)lipid metabolism
Supplementary Fig. 20	Cytotoxic stress diminishes Akt activation
Supplementary Fig. 21	Role of the RTK-PI3K-Akt-SREBP1-axis in controlling lipid metabolic gene expression and the PUFA-PC ratio

Supplementary Fig. 22	Expression of pro-survival kinases and kinase substrates upon stress
Supplementary Fig. 23	Activation status of kinases with metabolic and pro-survival function
Supplementary Fig. 24	Role of caspases in regulating Akt phosphorylation and PUFA-PC ratios
Supplementary Fig. 25	Sorafenib resistance lowers the cellular proportion of PUFAs in phospholipids
Supplementary Fig. 26	Kinetics of cell death induction by Akt inhibition in stressed and non-stressed cells
Supplementary Fig. 27	Kinetics of cell death induction by ACC inhibition in stressed and non-stressed cells
Supplementary Fig. 28	Constitutively active Akt suppresses apoptosis induction in stressed cells
Supplementary Fig. 29	Effect of constitutively active Akt (Akt-DD) on cell death induction in stressed cells
Supplementary Fig. 30	RTK ligands induce a shift from PUFA- to MUFA-containing phospholipids
Supplementary Fig. 31	Cytotoxic stress-induced PARP cleavage in cells with active and inactive ACC
Supplementary Fig. 32	Regulation of the ACSL isoenzyme pattern by cytotoxic stress
Supplementary Fig. 33	Manipulation of the cellular PUCA-PC ratio affects membrane peroxidation
Supplementary Fig. 34	Effect of apoptotic and cytotoxic stress on membrane peroxidation and sensitization to ferroptosis
Supplementary Fig. 35	Expression of RTKs and fatty acid biosynthetic enzymes in correlation with ferroptosis resistance
Supplementary Fig. 36	Regulation of proteins in redox homeostasis at the proteome level
Supplementary Fig. 37	Biosynthesis of isoprenoids via the mevalonate pathway
Supplementary Fig. 38	Uncropped versions of the Western Blots presented in Supplementary Fig. 2a
Supplementary Fig. 39	Uncropped versions of the Western Blots presented in Supplementary Fig. 8
Supplementary Fig. 40	Uncropped versions of the Western Blots presented in Supplementary Fig. 14a and b

Supplementary Fig. 41	Uncropped versions of the Western Blots presented in Supplementary Fig. 14c und d
Supplementary Fig. 42	Uncropped versions of the Western Blots (10 min, 1 h, 6 h) presented in Supplementary Fig. 20
Supplementary Fig. 43	Uncropped versions of the Western Blots (24 h) presented in Supplementary Fig. 20 (a) and 21 (b)
Supplementary Fig. 44	Uncropped versions of the Western Blots (p-mTOR and mTOR) presented in Supplementary Fig. 23
Supplementary Fig. 45	Uncropped versions of the Western Blots (p-p70/p85-S6K and p70/p85-S6K) presented in Supplementary Fig. 23
Supplementary Fig. 46	Uncropped versions of the Western Blots (p-IκB and IκB) presented in Supplementary Fig. 23
Supplementary Fig. 47	Uncropped versions of the Western Blots (p-GSK-3β and GSK-3β) presented in Supplementary Fig. 23
Supplementary Fig. 48	Uncropped versions of the Western Blots (p-MARCKS and MARCKS) presented in Supplementary Fig. 23
Supplementary Fig. 49	Uncropped versions of the Western Blots (p-ERK-1/2 and ERK-1/2) presented in Supplementary Fig. 23
Supplementary Fig. 50	Uncropped versions of the Western Blots presented in Supplementary Fig. 24a and b
Supplementary Fig. 51	Uncropped versions of the Western Blots presented in Supplementary Fig. 26a and b
Supplementary Fig. 52	Uncropped versions of the Western Blots presented in Supplementary Fig. 28
Supplementary Fig. 53	Uncropped versions of the Western Blots presented in Supplementary Fig. 31
Supplementary Fig. 54	Gating strategies

Supplementary Tables

Supplementary Table 1	Oxidized 20:4 fragment ions selected in Q3
Supplementary Table 2	Retention times and MRM transitions for oxidized PE quantitation

Supplementary Notes

Supplementary Note 1 | Induction of programmed cell death in fibroblasts by multiple pathways

Supplementary Note 2 | Cytotoxic stress increases the PUFA-PC ratio independently of caspases

Supplementary Note 3 | Akt impairs apoptotic progression in fibroblasts

Supplementary Note 4 | Cytotoxic regulation of the redox proteome

Supplementary Note 5 | Individual mechanisms of specific cytotoxic stressors

Supplementary Note 6 | Putative relevance to phenotypes of ACC-deficient mice

Supplementary Note 7 | Multiple cytotoxic mechanisms suppress fatty acid biosynthesis

Supplementary Note 8 | Cytotoxic stress impacts the CL profile

Supplementary Note 9 | Experimental setup for FLIM

Supplementary References

Supplementary Figures

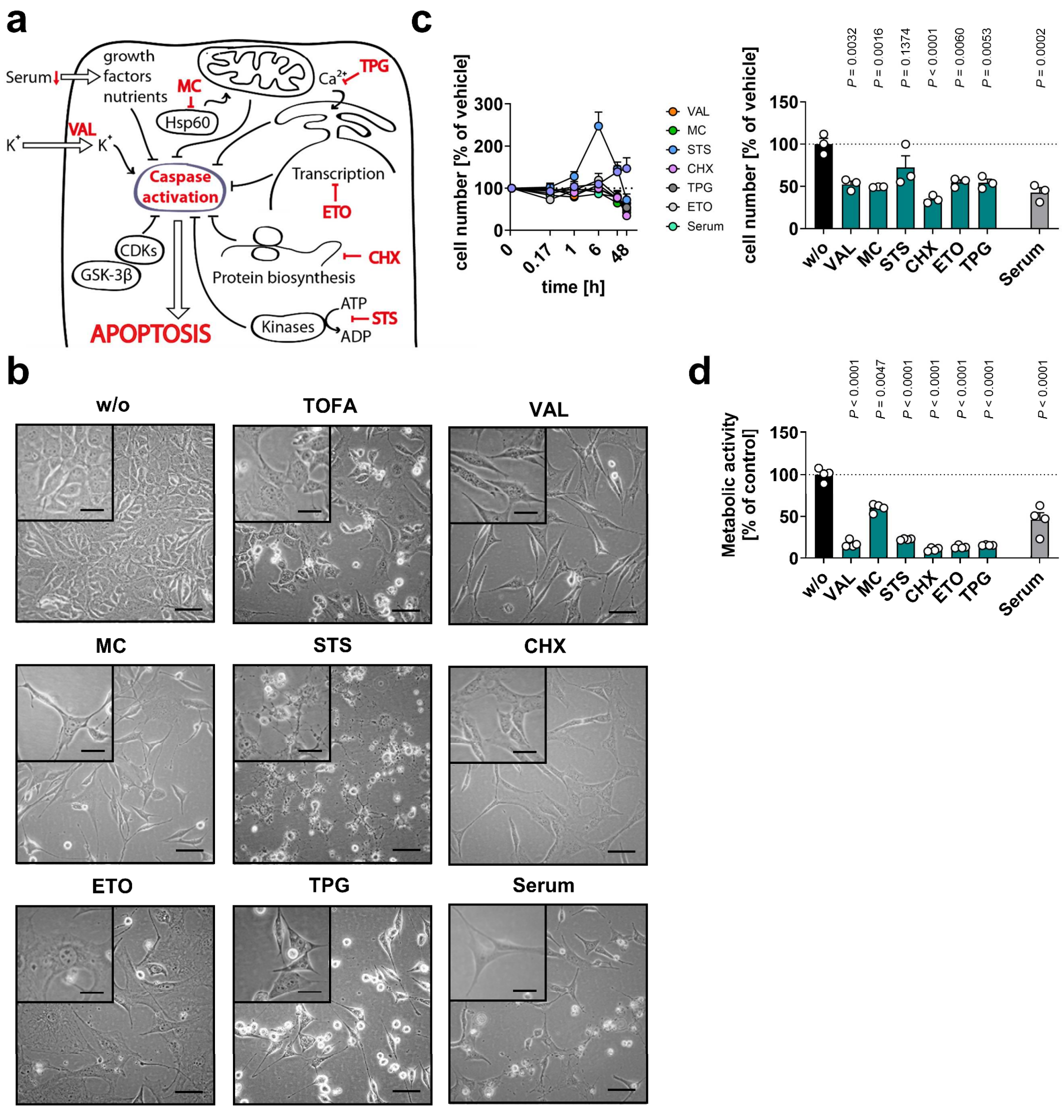

Supplementary Fig. 1 Fibroblast morphology, numbers, and viability under cytotoxic conditions. **a** Schematic overview about the molecular mechanisms of the cytotoxic stressors used in this study. CDK, cyclin-dependent kinase; GSK, glycogen synthase kinase. **b-d** Fibroblasts were cultivated under diverse cytotoxic conditions for 48 h (**b**, **d**) or as indicated (**c**). **b** Phase contrast images after 48 h; scale bar of the outer box: 25 µm, scale bar of the insert: 10 µm. Images are representative of three independent experiments. **c** Cell numbers over time (left panel) or at 48 h (right panel). **d** Metabolic activity measured by MTT assay. Mean + s.e.m. (**c**, left panel) and single data (**c**, right panel; **d**) from $n = 3$ (**c**), $n = 4$ (**d**) independent experiments. *P* values (**c**, right panel; **d**) vs. vehicle control; repeated measures one-way ANOVA of log-transformed data + Dunnett's post hoc test. The exact *P* values of panels **c** (right panel) and **d** are given in the Supplementary Data 4.

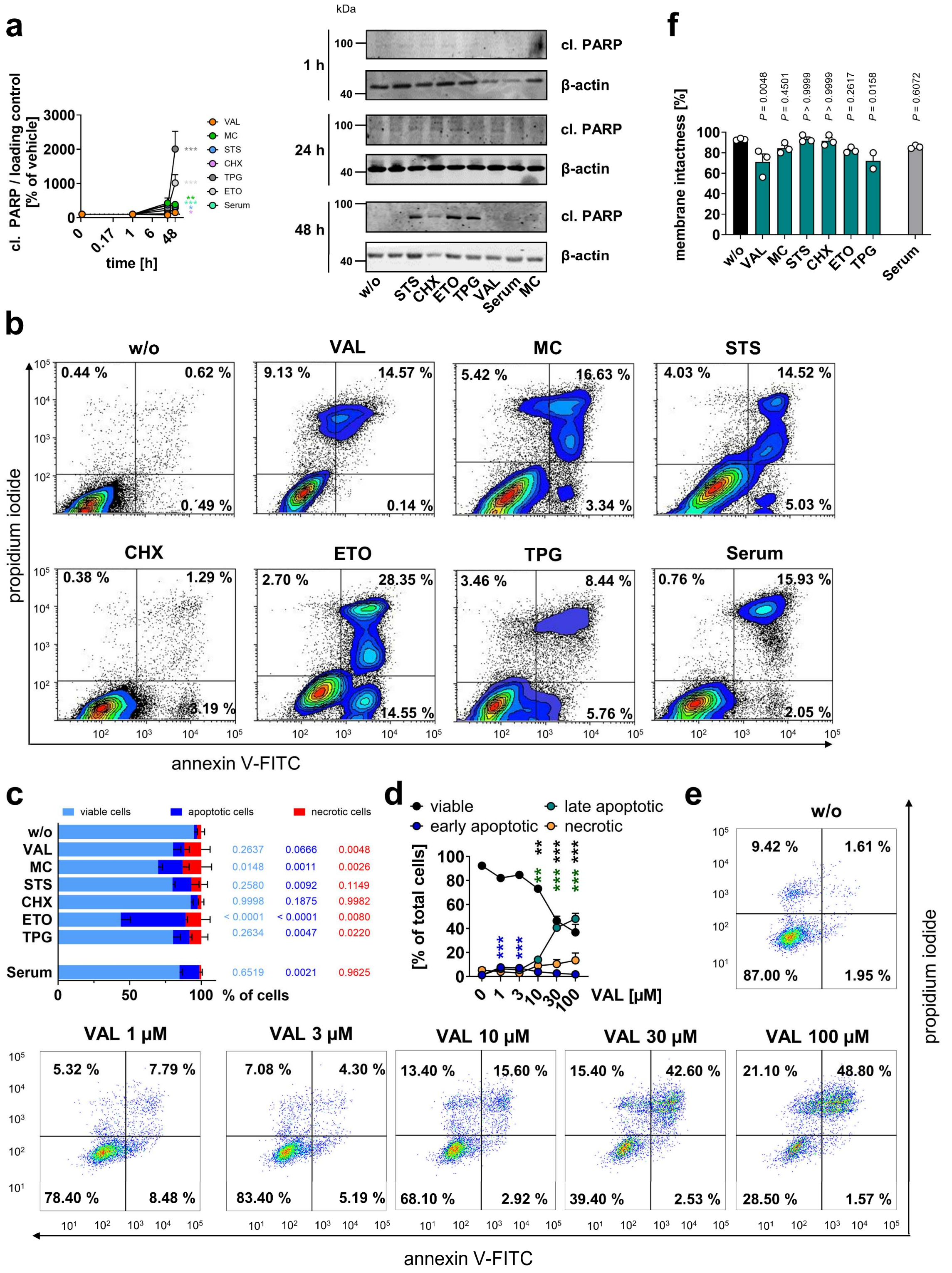

Supplementary Fig. 2 PARP cleavage, PS externalization, cell viability, and membrane intactness. Fibroblasts were cultivated under cytotoxic conditions for 48 h (**b-f**) or as indicated (**a**). **a** Time-dependent cleavage of PARP (normalized to loading control); cl., cleaved. Western blots are representative of eleven (48 h) or two (1 h and 24 h) independent experiments. Bands at 1 h were considered as not detectable and set to 100%. **b-e** Annexin V and propidium iodide (PI) staining. **b**, **e** Cytograms are representative of three independent experiments. **c**, **d** Proportion of annexin V / PI negative cells (viable cells), annexin V positive cells (apoptotic cells), and annexin V negative / PI positive cells (necrotic cells) as percentage of total cells. **f** Membrane intactness determined by trypan

blue staining. Mean + s.e.m. (**a**, **c, d**) and single data (**f**) from *n* = 2 (**a** for 24 h), *n* = 3 (**c, d**, **f**) and *n* =11 (**a** for 48 h) independent experiments. *P < 0.05, **P < 0.01, ***P < 0.001 (**a**, **d**) or *P* values (**a**, **c**, **d**, **f**) vs. vehicle control; mixed-effects model (REML) (**f**) or repeated measures one-way ANOVA + Dunnett's post hoc test (**d**) of log-transformed data (**a**, **c**). Exact *P* values for panel **a** (at 48 h) and **c**, **d**, **f** are given in Supplementary Data 4. Uncropped Western blots are shown in Supplementary Fig. 38.

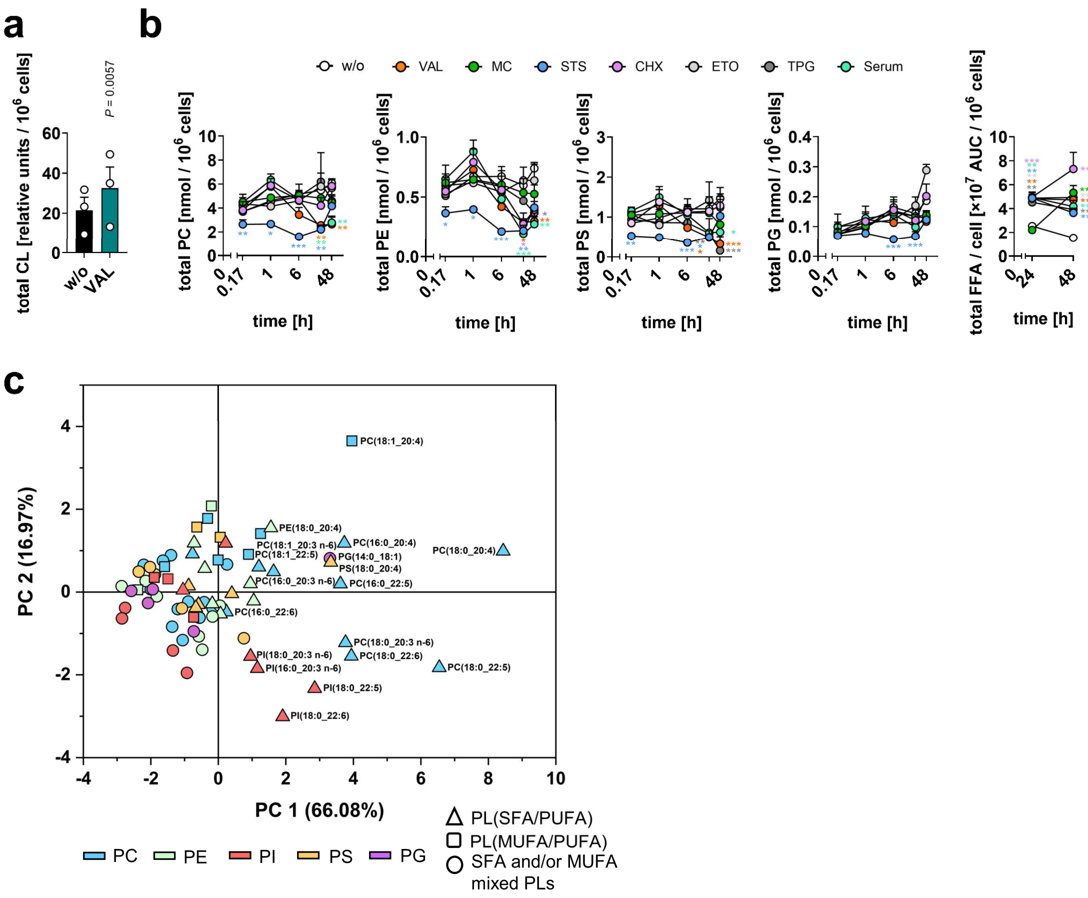

Supplementary Fig. 3 Cytotoxic stress-induced changes in phospholipid content and composition. Fibroblasts were cultured under cytotoxic stress conditions for 48 h (**a**, **c**) or as indicated (**b**). **a** Cellular content of CL. **b** Time-dependent changes of the cellular content of PC, PE, PS, PG, and FFA. Mean + s.e.m. (**b**) and single data (**a**) from $n = 3$ independent experiments. $^*P < 0.05$, $^{**}P < 0.01$, $^{***}P < 0.001$ (**b**) or P values (**a**) vs. vehicle control (**a**) for the respective time point (**b**); repeated measures one-way ANOVA of log-transformed data + Dunnett's post hoc test (**b**) or two-tailed paired t-test of log-transformed data (**a**). Exact P values for panel **b** are given in Supplementary Data 4. **c** Principal component analysis of mean percentage changes in phospholipid proportions from three (PE, PS, PI, PG) or four (PC) independent experiments relative to vehicle control. For PI, previously published data were reanalyzed.[1]

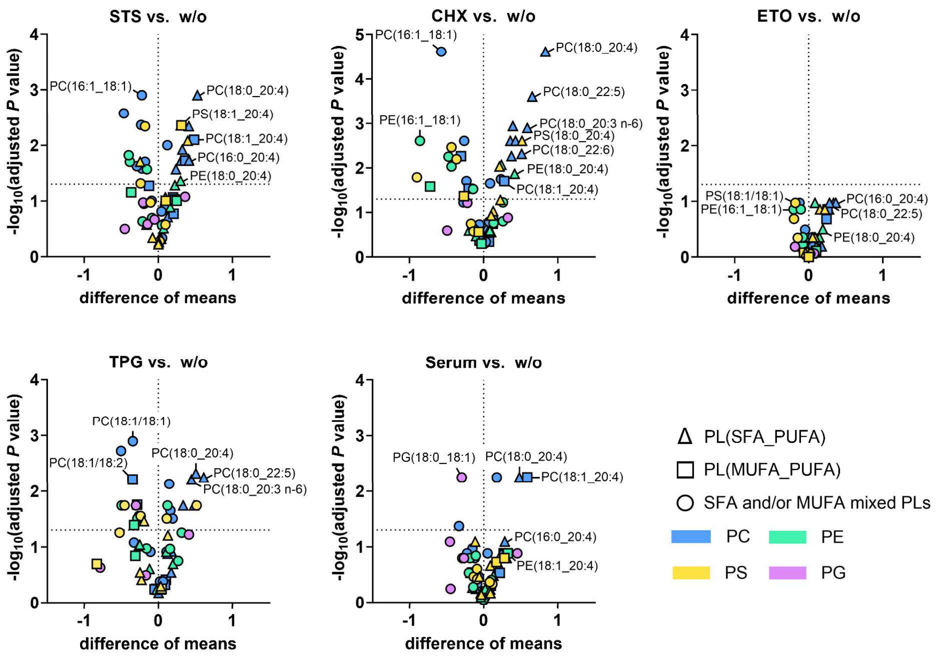

Supplementary Fig. 4 Phospholipid species regulated by cytotoxic stress. Fibroblasts were cultured under cytotoxic stress conditions for 48 h. Volcano plots indicating phospholipids, whose proportions are strongly and significantly altered by cytotoxic stress conditions. Comparisons of the indicated treatment groups show the mean difference of percentage changes and the negative $\log_{10}$(adjusted *P* value) from three (PE, PG, PS) or four (PC) independent experiments. Mean values and adjusted *P* values given vs. vehicle control from $n = 3$ independent experiments; two-tailed multiple unpaired student *t*-tests from log-transformed data with correction for multiple comparisons using a two-stage linear step-up procedure by Benjamini, Krieger, and Yekutieli (false discovery rate 5%).

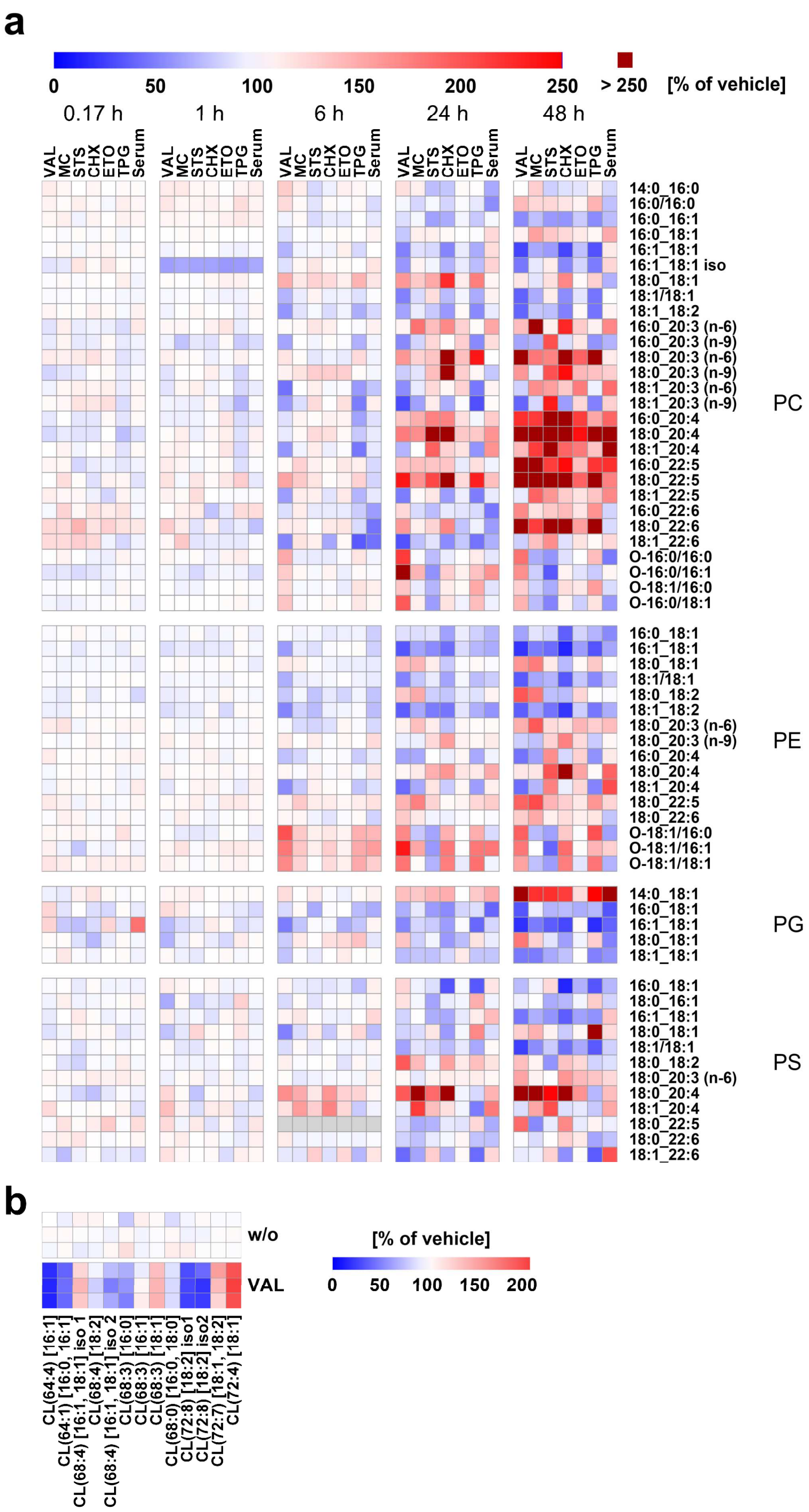

Supplementary Fig. 5 Phospholipid fatty acid profile of stressed cells. Fibroblasts were cultured under cytotoxic stress conditions for 48 h (**b**) or as indicated (**a**). Heatmaps showing changes in the PC, PE, PG, and PS (**a**) or CL (**b**) profile as compared to vehicle control. Data are given as percentage of the proportion of phospholipid species relative to vehicle control for each time point. Mean (**a**) or single data (**b**) from *n* = 3 (PE, PG, PS, CL) or n = 4 (PC) independent experiments.

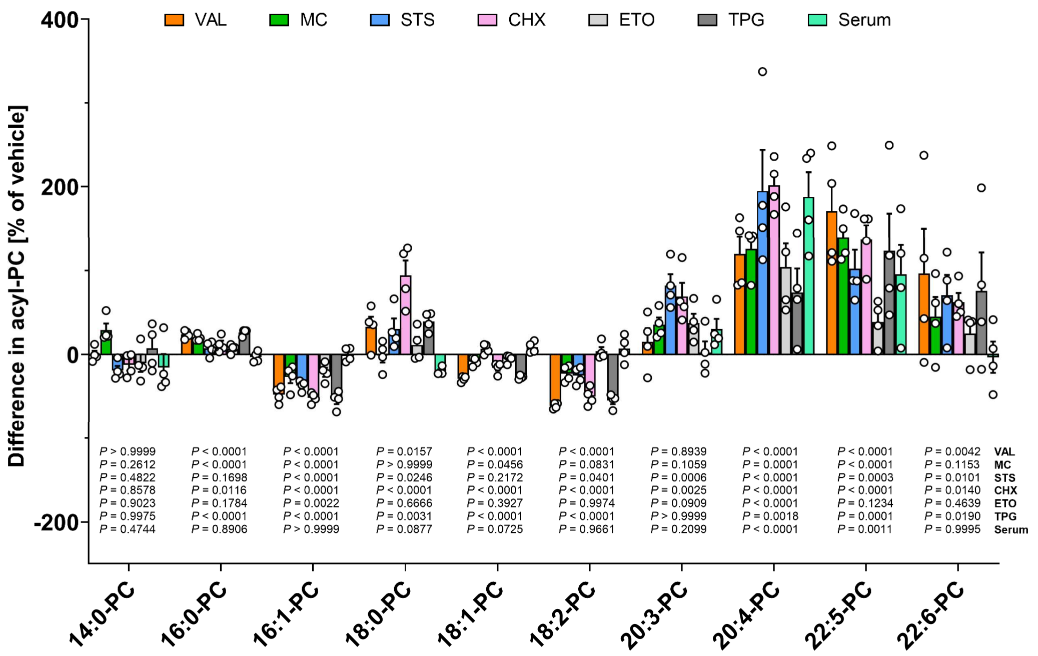

Supplementary Fig. 6 Cytotoxic stress-induced changes in the PC fatty acid distribution. Fibroblasts were cultured under cytotoxic stress conditions for 48 h, and percentage differences in the proportion of individual fatty acids in PC are shown. A difference of 0% in acyl-PC indicates no change relative to vehicle control. Mean + s.e.m. and single data from *n* = 4 independent experiments. *P* values vs. vehicle control; repeated measures ANOVA of log-transformed data + Dunnett's post hoc test. Exact *P* values are given in Supplementary Data 4.

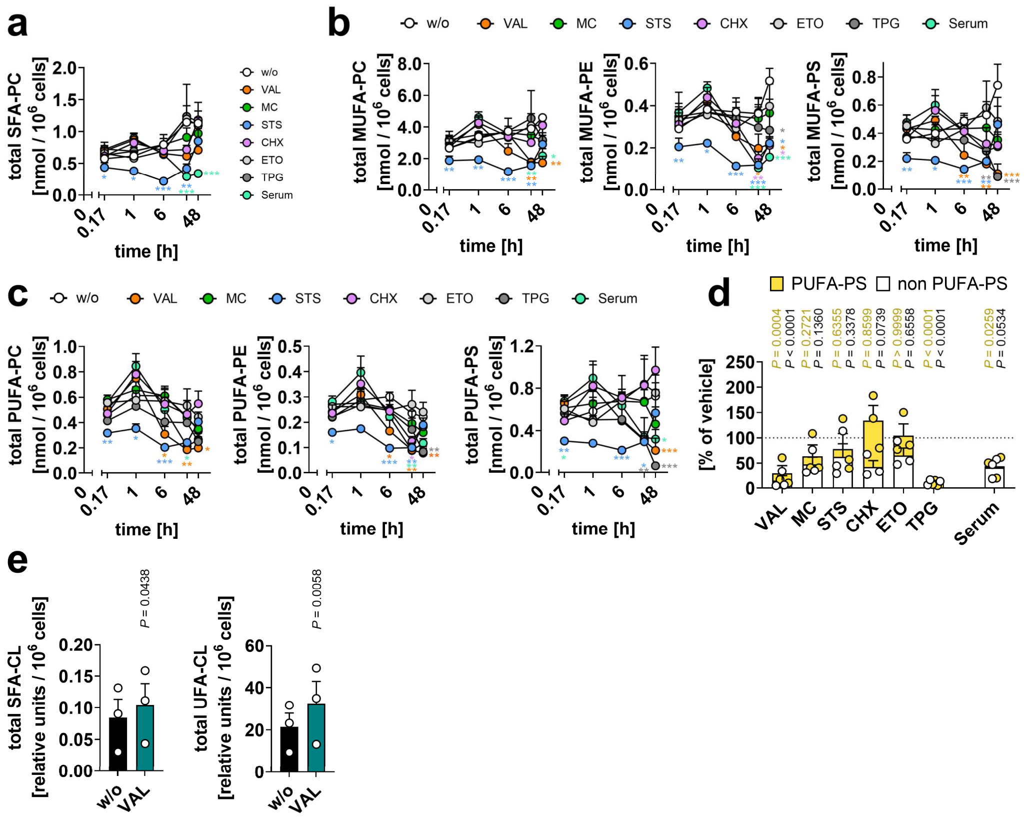

Supplementary Fig. 7 Phospholipid unsaturation under cytotoxic stress. Fibroblasts were cultured under cytotoxic stress conditions for 48 h (**d**, **e**) or as indicated (**a-c**). **a-c** Time-dependent changes of the cellular content of SFA- (**a**), MUFA- (**b**), and PUFA-containing PC, PE, or PS (**c**). **d** Overlaid changes in the cellular content of PS that either contain or are free of PUFAs. **e** Cellular content of SFA- and UFA-containing CL. Mean + s.e.m. (**a-c**) and single data (**d**, **e**) from $n = 3$ independent experiments. *$P < 0.05$, **$P < 0.01$, ***$P < 0.001$ (**a-c**) or P values (**d**, **e**) vs. vehicle control (**a-c**, **e**) for the respective time point or PUFA-PS and non PUFA-PS of the vehicle control, respectively (**d**); repeated measures one-way ANOVA of log-transformed data + Dunnett's post hoc test (**a-d**) or two-tailed paired *t*-test of log-transformed data (**e**). Exact P values for panel **a-d** are given in Supplementary Data 4.

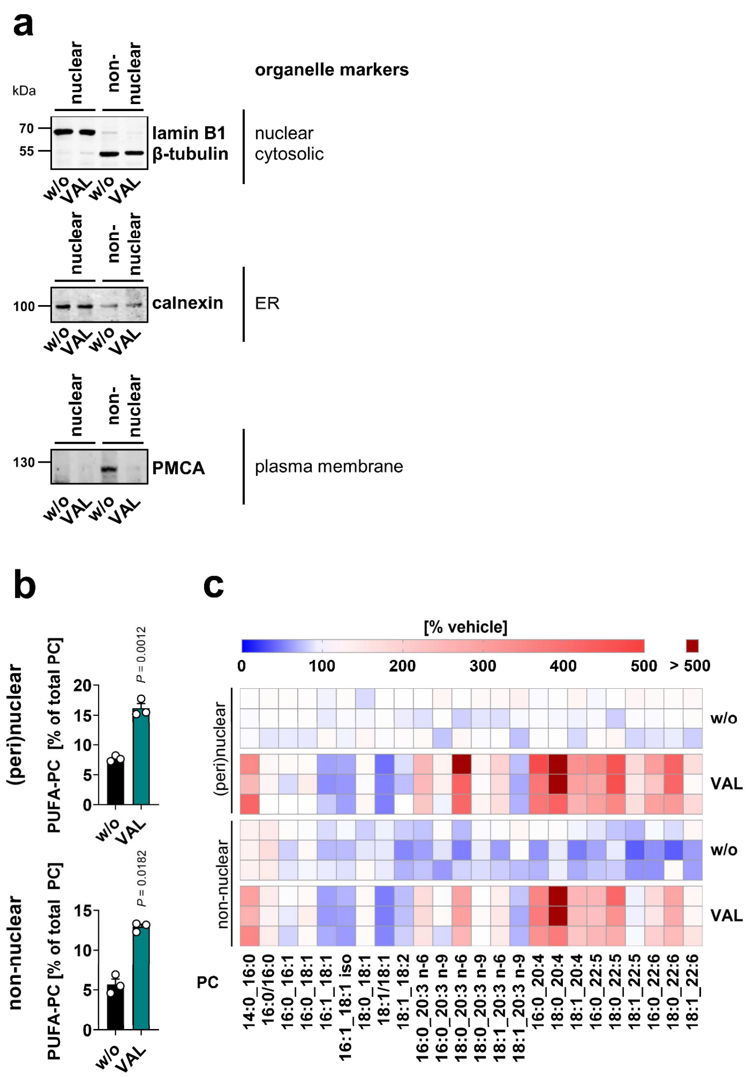

Supplementary Fig. 8 Subcellular fractionation of VAL-treated fibroblasts. Cells were treated with vehicle or VAL (10 μM) for 48 h before (peri)nuclear and non-nuclear membranes were separated by differential centrifugation. **a** Protein levels of the nuclear marker lamin B1, the cytosolic marker β-tubulin, the ER marker calnexin, and the plasma membrane Ca^{2+} ATPase (PMCA), which degrades during apoptosis. Western blots are representative of three independent experiments. Uncropped Western blots are shown in Supplementary Fig. 39. **b** PUFA-PC ratio of the (peri)nuclear and non-nuclear fraction. **c** Heatmap showing changes in the PC profile as compared to the (peri)nuclear fraction of the vehicle control. Data are given as percentage changes in the proportion of PC species relative to the average proportion in the perinuclear fraction of vehicle-treated cells. Mean + s.e.m. and single data (**b**) or single data (**c**) from $n = 3$ independent experiments. P values vs. vehicle control (**b**); two-tailed paired student t-test of log-transformed data (**b**).

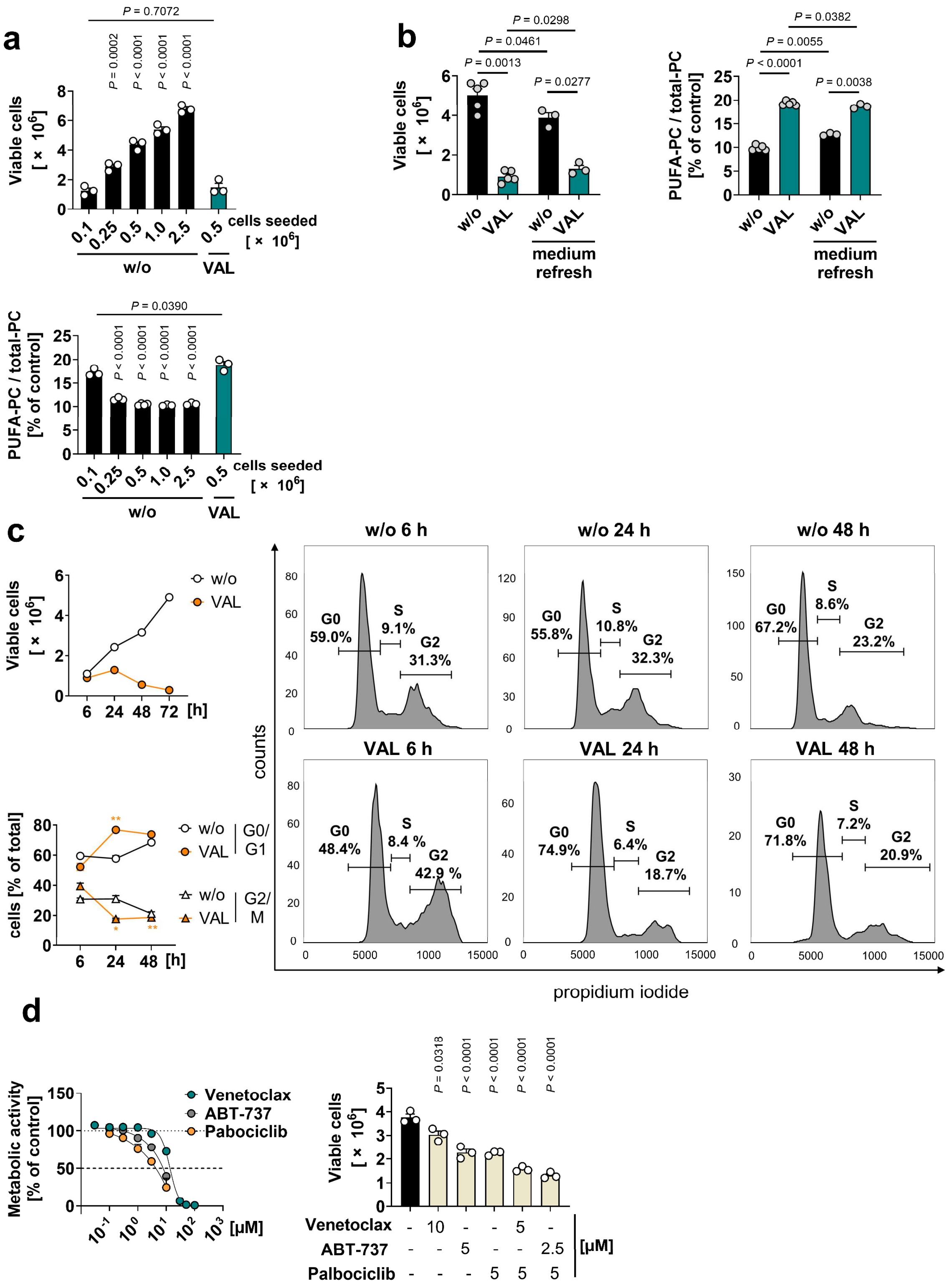

Supplementary Fig. 9 Dynamics of cell density and cell cycle progression and effect of medium exchange. **a**, **b** Number of viable NIH-3T3 fibroblasts and cellular proportion of PUFA-PC after 48 h of cultivation depending on the number of cells seeded (**a**) or on the treatment with VAL (10 µM) and medium + VAL exchange after 24 h (**b**). Two datasets in **a** (0.5×10^6 cells; w/o and VAL) are identical to **b** (without medium refresh; w/o and VAL). **c** Cell cycle progression of NIH-3T3 fibroblasts treated with VAL. Cytograms are representative of three independent experiments (**c**, right panel). **d** Metabolic activity (measured by MTT assay) and viable numbers of NIH-3T3 cells treated with the BCL-2 inhibitor Venetoclax, the BCL-2 family inhibitor ABT-737, and the CDK4/6 inhibitor

Palbociclib for 48 h. Single data (**c,** left upper panel) and mean + s.e.m. (**c,** left lower panel; **d**, left panel) and single data (**a**, **b**, **d**, right panel) from $n = 3$ independent experiments. *$P < 0.05$, **$P < 0.01$, ***$P < 0.001$ (**c**, lower left panel) or *P* values (**a**, **b**, **d**) vs. control (**a**: 1×10^5 seeded cells; **b**, **c**, **d**: vehicle control at the indicated time point); repeated measures one-way ANOVA (**d**) or mixed-effects model (REML) (**a**) of log-transformed data + Dunnett's post hoc test or two-tailed paired student *t*-test (**c**) of log-transformed data (**b**). Exact *P* values for panel **a-d** are given in Supplementary Data 4.

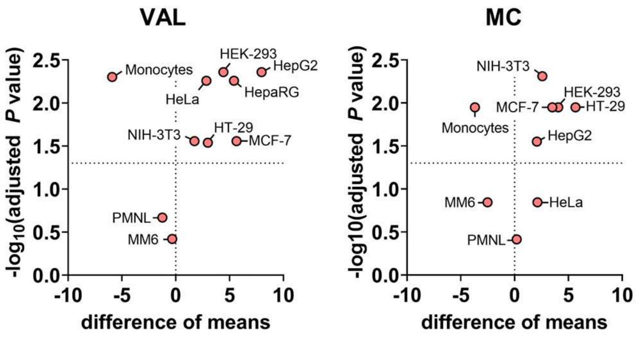

Supplementary Fig. 10 Proportion of PUFA-PC in cancer- and non-cancer cells under cytotoxic stress. Fibroblasts were treated with vehicle, VAL (10 µM), or MC (10 µM) for 48 h. Volcano plots indicating cell lines or primary cells, whose PUFA-PC proportion is strongly and significantly altered by VAL or MC. Comparisons of the indicated treatment groups show the mean difference of percentage changes and the negative $\log_{10}$(adjusted *P* value) from $n = 3$ (HEK-293, HeLa, HepaRG (only VAL), HepG2, HT-29, MCF-7, MM6), $n = 4$ (Monocytes, NIH-3T3) and $n = 5$ (PMNL) independent experiments. Mean values and adjusted *P* values given vs. vehicle control; two-tailed multiple paired student *t*-tests with correction for multiple comparisons using a two-stage linear step-up procedure by Benjamini, Krieger, and Yekutieli (false discovery rate 5%).

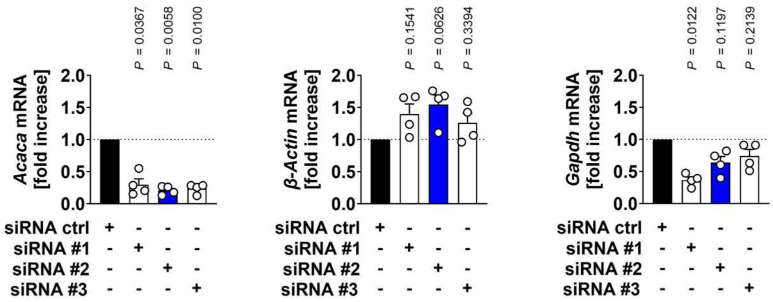

Supplementary Fig. 11 Knockdown of ACC1 in fibroblasts. ACC1 was silenced using three different siRNA sequences (#1-3), and cells were harvested after 48 h. Non-targeting siRNA was transfected as control (ctrl). *Acc*, *β-actin* and *Gapdh* mRNA levels were normalized to the total amount of cellular RNA. Mean + s.e.m. and single data from n = 4 independent experiments. P values vs. non-targeting siRNA control; repeated measures one-way ANOVA of log-transformed data + Dunnett's post hoc test.

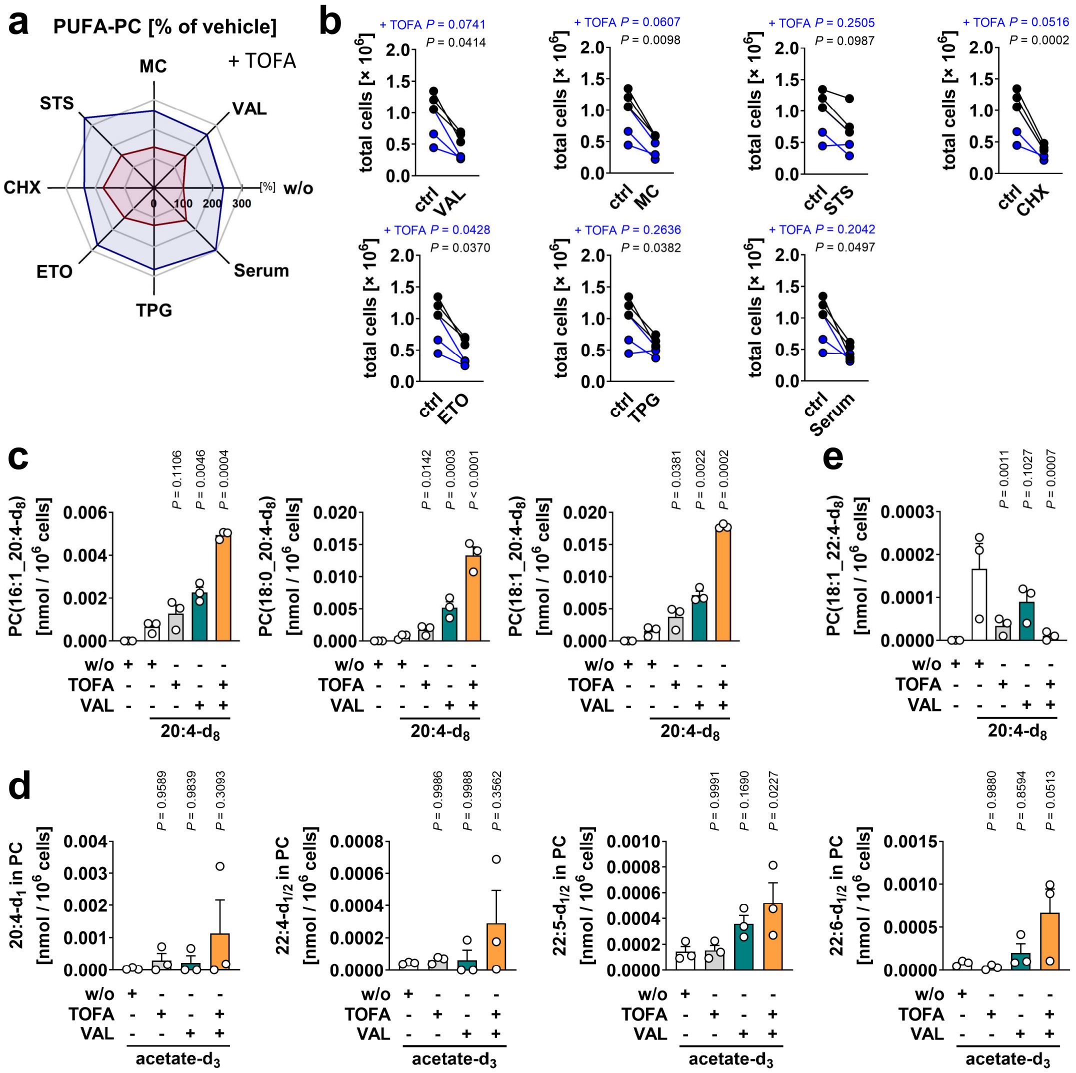

Supplementary Fig. 12 Contribution of ACC to PUFA metabolism under cytotoxic stress. **a**, **b** Fibroblasts were exposed to cytotoxic stress for 48 h in presence and absence of the selective ACC inhibitor TOFA. The radar chart indicates the percentage changes of PUFA-PC ratios relative to vehicle control (**a**) and the line charts present total cell number (**b**). Interconnected lines indicate data from the same independent experiment. **c-e** Fibroblasts were supplemented with 20:4-d_8 (1 µM) (**c**, **e**) or acetate-d_3 (30 µM) (**d**) before being treated with vehicle, TOFA (5 µM), and/or VAL (10 µM) for 48 h. **c** Cellular content of PC species containing 20:4-d_8. **d** Cellular content of PC species with either one or two acetate-d_3 incorporated into 20:4, 22:4, 22:5, or 22:6. **e** Cellular content of a PC species containing 22:4 derived from the elongation of 20:4-d_8. Mean (**a**), paired data (**b**), or mean + s.e.m. and single data (**c-e**) from n = 3 independent experiments. P values vs. vehicle control (**b-e**); two-tailed paired student t-test of log-transformed data (**b**), repeated measures one-way ANOVA (**c**, **d**) or mixed-effects model (REML) (**e**) of log-transformed data (**c**, **e**) + Dunnett's post hoc test. Exact P values for panel **c** are given in Supplementary Data 4.

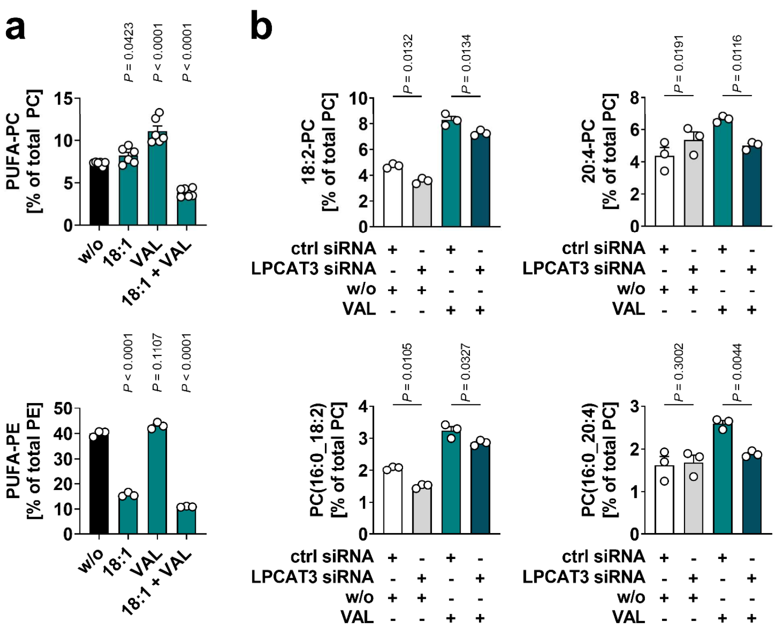

Supplementary Fig. 13 The stress-induced increase in PUFA-PC ratios depends on MUFA availability and LPLAT12. **a** Fibroblasts were supplemented with 18:1 (100 µM) and treated with vehicle or VAL (10 µM). The cellular proportion of PUFA-PC was determined after 48 h. **b** Fibroblasts with silenced LPLAT12/LPCAT3 were treated with vehicle or VAL, and the cellular proportion of PC subclasses and species was determined after 48 h. Non-targeting siRNA was transfected as control (ctrl siRNA). Mean + s.e.m. and single data from $n = 3$ (**a** for PE, **b**) and $n = 6$ (**a** for PC) independent experiments. *P* values vs. vehicle control (**a**) or ctrl-transfected cells (**b**); repeated measures one-way ANOVA of log-transformed data + Dunnett's post hoc test (**a**) or paired student *t*-test of log-transformed data (**b**). Exact *P* values for panel **a** are given in Supplementary Data 4.

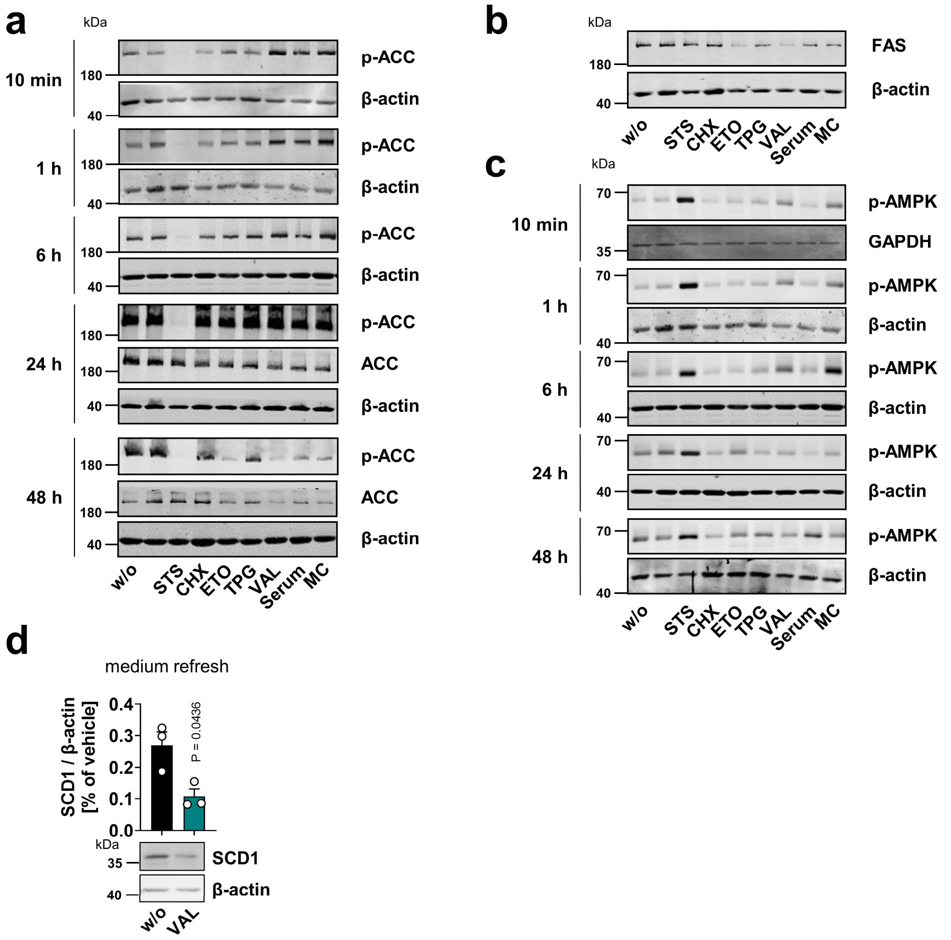

Supplementary Fig. 14 Regulation of key enzymes in *de novo* fatty acid biosynthesis. Fibroblasts were cultured under cytotoxic stress conditions for 48 h (**b**) or as indicated (**a**, **c**) with the culture medium + VAL (10 µM) being replaced after 24 h (**d**). Phosphorylation of ACC (**a**) and AMPK (**c**) and expression of ACC (**a**), FAS (**b**), and SCD1 (**d**). Western Blots are representative of three (**a**, **c**, **d**) or six (**b**) independent experiments. Blots for the loading control β-actin at 1 h and 6 h are identical in **a** and **c**, and blots for β-actin at 10 min (**a**), 1 h (**a**, **c**) and 24 h (**c**) are identical to Thürmer et al.[2]. Mean + s.e.m. and single data (**d**). *P* values vs. vehicle control; paired student *t*-test of log-transformed data (**d**). Uncropped Western blots are shown in Supplementary Fig. 40 and 41.

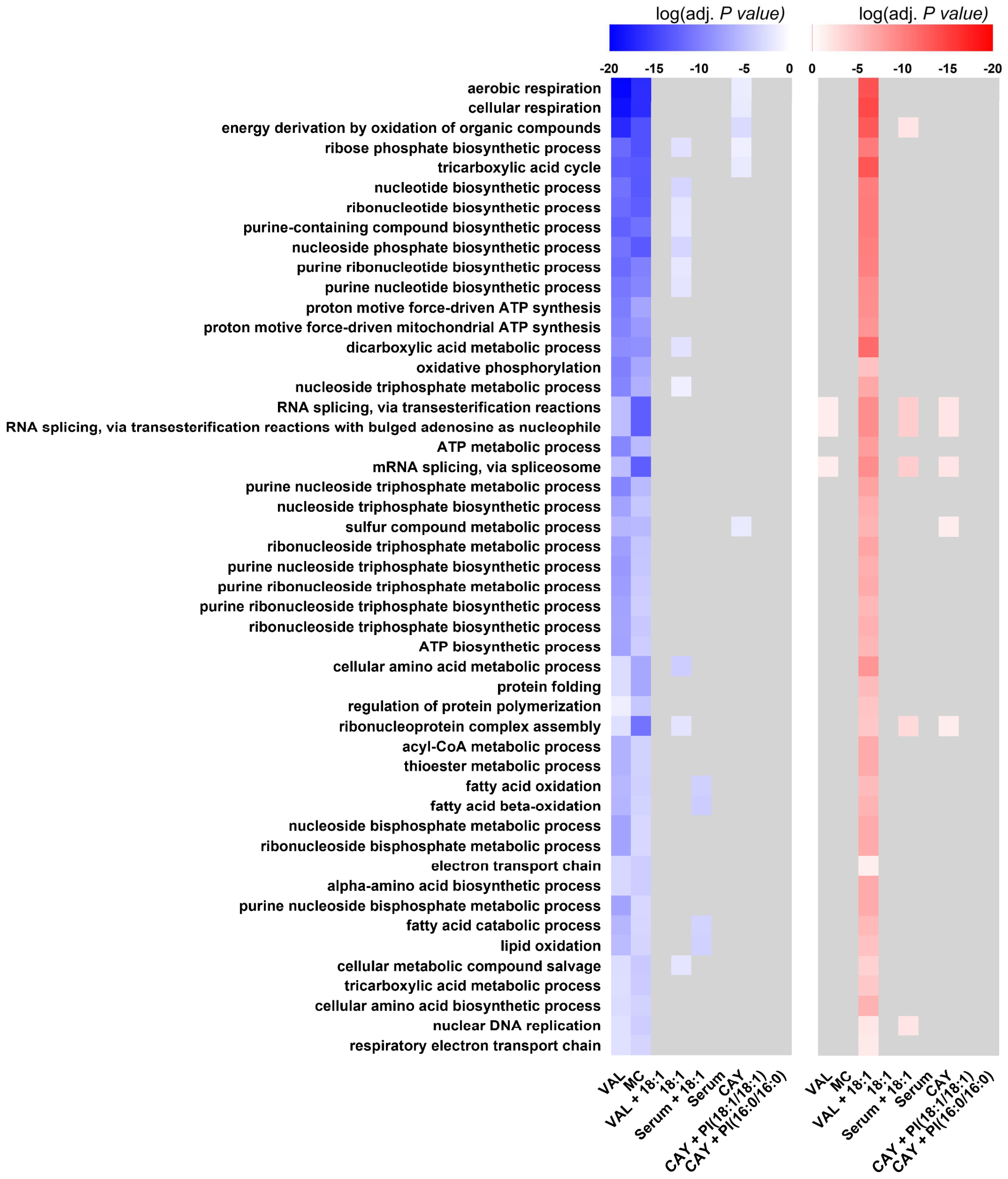

Supplementary Fig. 15 GO term enrichment analysis of biological processes. Fibroblasts were treated with vehicle, VAL (10 µM), MC (10 µM), 18:1 (100 µM), VAL (10 µM) plus 18:1 (100 µM), CAY10566 (CAY, 3 µM), or CAY (3 µM) plus PI(18:1/18:1) (50 µM) or the saturated control PI(16:0/16:0) (50 µM), or were serum starved (serum) in presence or absence of 18:1 (100 µM) for 48 h. Proteins were considered to be upregulated or downregulated with fold changes of at least $\log_2(1.5)$ or $-\log_2(1.5)$, respectively, and an adjusted *P*-value < 0.05 (Benjamini-Hochberg correction). The left heatmap lists the 49 biological processes among the top 100 enriched ones that were most significantly depleted from the considered proteins by both VAL and MC relative to vehicle control when separately ranking them for the two stressors and calculating the average position (from top to bottom). Log_{10}(adjusted *P* values) are visualized by color. The right heatmap indicates for the same set of biological processes those that are significantly enriched in the considered proteins relative to vehicle control with fold changes ≥ 0.5 and an adjusted P-value < 0.05. Independent experiments: n = 3, except n = 2 for serum depletion.

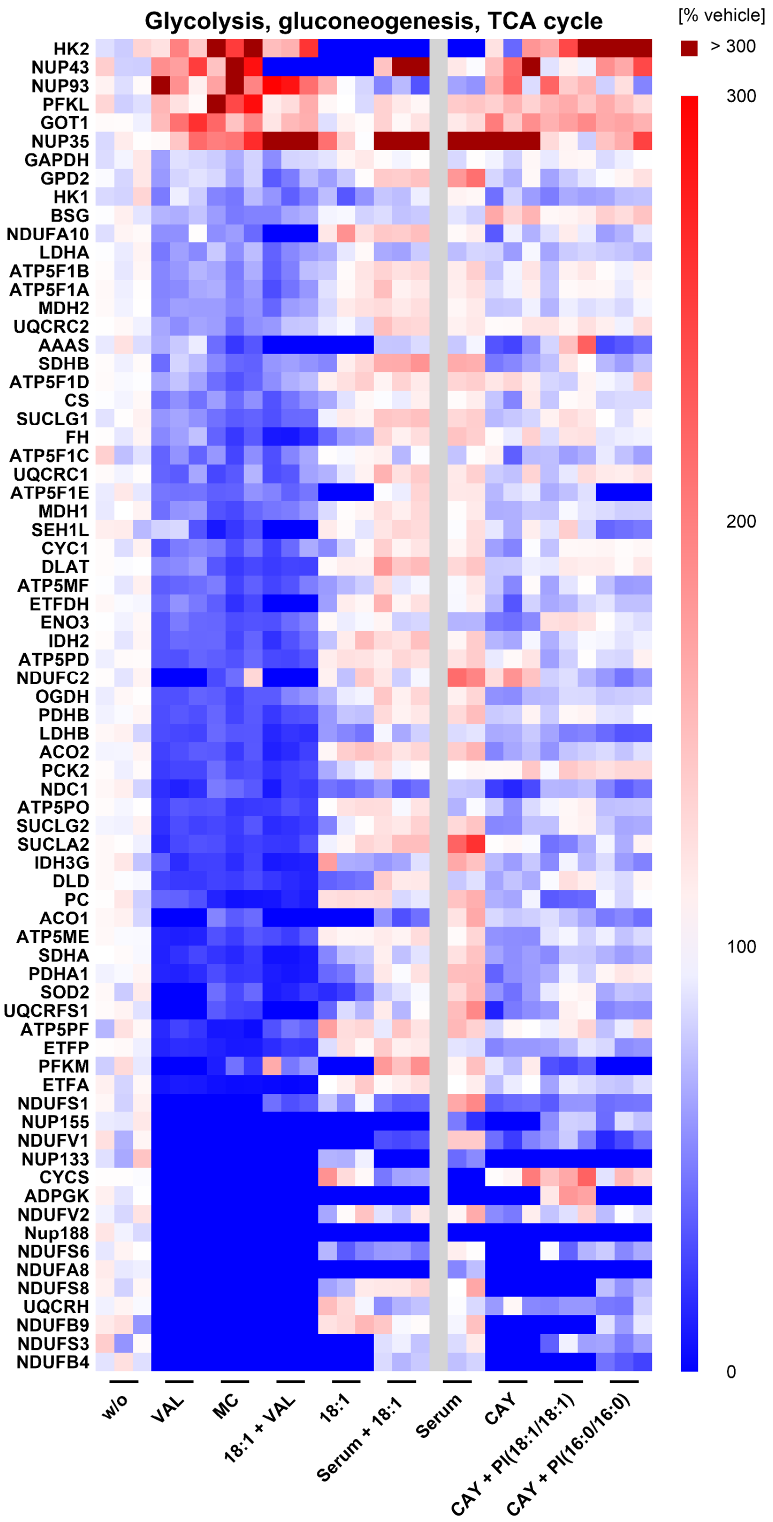

Supplementary Fig. 16 Stress-induced changes in the availability of proteins related to glycolysis, gluconeogenesis, and the TCA cycle. Fibroblasts were treated with vehicle, VAL (10 µM), MC (10 µM), 18:1 (100 µM), VAL (10 µM) plus 18:1 (100 µM), CAY10566 (CAY, 3 µM), or CAY (3 µM) plus PI(18:1/18:1) (50 µM) or the saturated control PI(16:0/16:0) (50 µM), or were serum starved (serum) in presence or absence of 18:1 (100 µM) for 48 h. The heatmap shows relative changes in protein levels. Focus is placed on proteins that are up- or downregulated by VAL and MC in the same

direction (≥ 20%). Single data of independent experiments (*n* = 3, except *n* = 2 for serum depletion) were calculated as percentage of vehicle control.

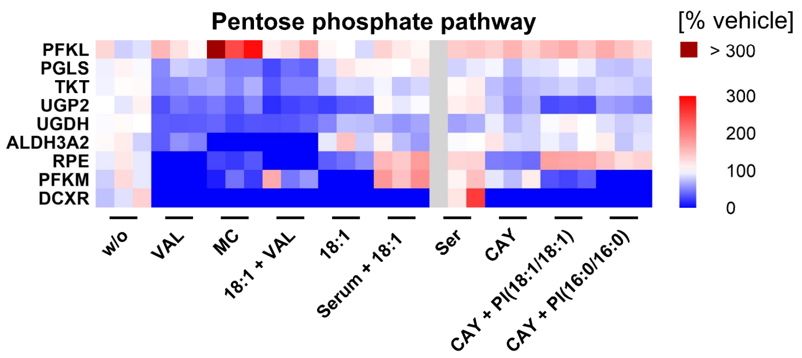

Supplementary Fig. 17 Stress-induced changes in the availability of proteins related to the pentose phosphate cycle. Fibroblasts were treated with vehicle, VAL (10 µM), MC (10 µM), 18:1 (100 µM), VAL (10 µM) plus 18:1 (100 µM), CAY10566 (CAY, 3 µM), or CAY (3 µM) plus PI(18:1/18:1) (50 µM) or the saturated control PI(16:0/16:0) (50 µM), or were serum starved (serum) in presence or absence of 18:1 (100 µM) for 48 h. The heatmap shows relative changes in protein levels. Focus is placed on proteins that are up- or downregulated by VAL and MC in the same direction (≥ 20%). Single data of independent experiments (n = 3, except n = 2 for serum depletion) were calculated as percentage of vehicle control.

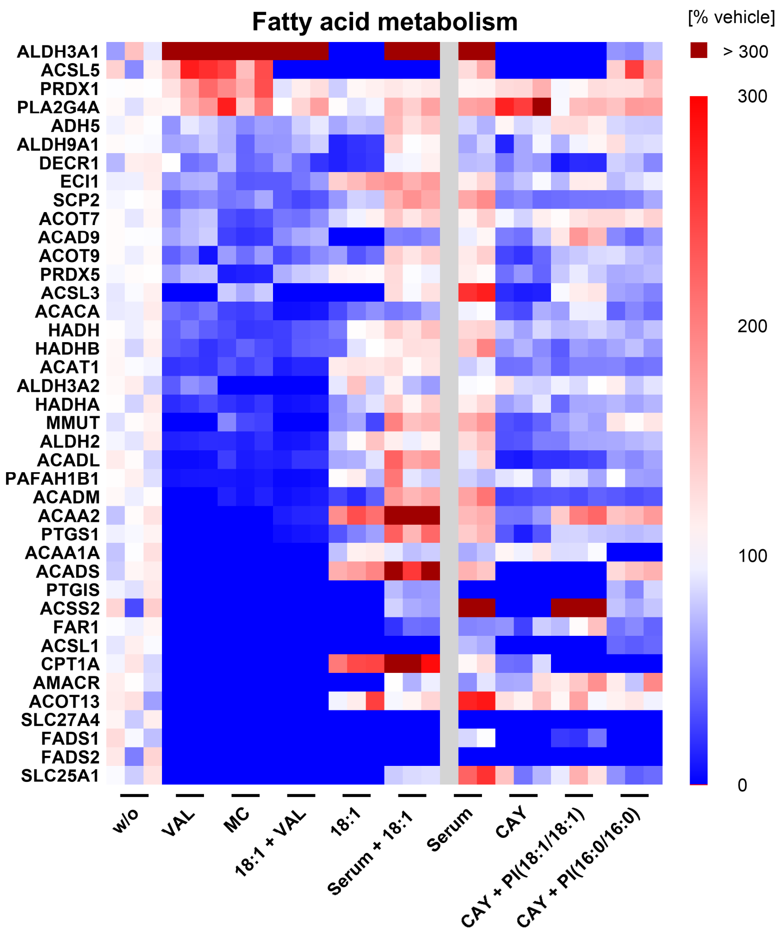

Supplementary Fig. 18 Stress-induced changes in the availability of proteins related to fatty acid metabolism. Fibroblasts were treated with vehicle, VAL (10 µM), MC (10 µM), 18:1 (100 µM), VAL (10 µM) plus 18:1 (100 µM), CAY10566 (CAY, 3 µM), or CAY (3 µM) plus PI(18:1/18:1) (50 µM) or the saturated control PI(16:0/16:0) (50 µM), or were serum starved (serum) in presence or absence of 18:1 (100 µM) for 48 h. The heatmap shows relative changes in protein levels. Focus is placed on proteins that are up- or downregulated by VAL and MC in the same direction (≥ 20%). Single data of independent experiments (*n* = 3, except *n* = 2 for serum depletion) were calculated as percentage of vehicle control.

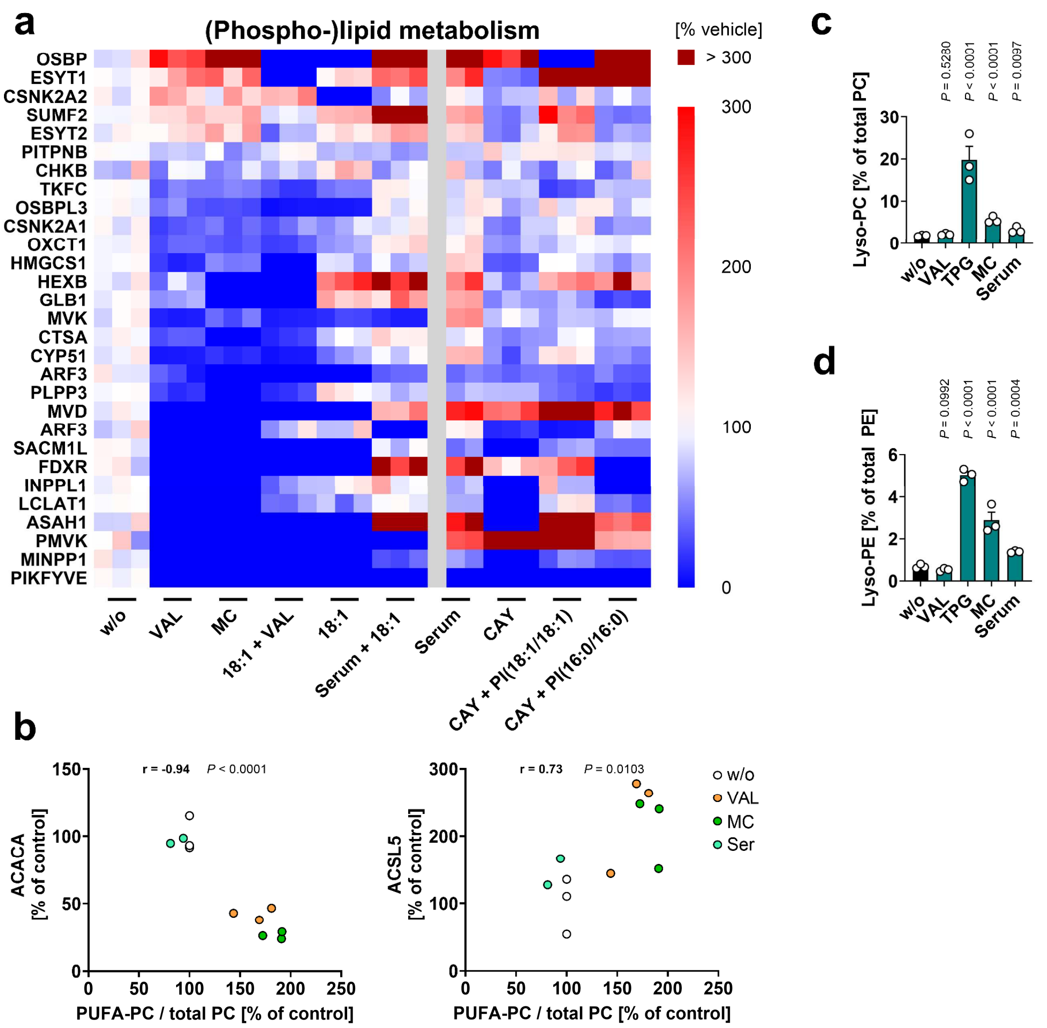

Supplementary Fig. 19 Stress-induced changes in the availability of lysophospholipids and proteins related to (phospho-)lipid metabolism. Fibroblasts were treated with vehicle, VAL (10 µM), MC (10 µM), 18:1 (100 µM), VAL (10 µM) plus 18:1 (100 µM), CAY10566 (CAY, 3 µM), or CAY (3 µM) plus PI(18:1/18:1) (50 µM) or the saturated control PI(16:0/16:0) (50 µM), or were serum starved (serum) in presence or absence of 18:1 (100 µM) for 48 h. **a** The heatmap shows relative changes in protein levels. Focus is placed on proteins that are up- or downregulated by VAL and MC in the same direction (≥ 20%). Single data of independent experiments (*n* = 3, except *n* = 2 for serum depletion) were calculated as percentage of vehicle control. **b** Pearson correlation between PUFA-PC ratio and relative protein expression of ACACA and ACSL5 for the independent datasets of vehicle, VAL, MC and Serum. **c**, **d** Cellular proportion of lyso-PC (**c**) and lyso-PE (**d**), summarizing 14:0- (only for lyso-PC), 16:0-, 16:1-, 18:0-, 18:1-, 18:2-, 20:3-, 20:4-, and 22:5-containing species. Single data (**a, b**) or mean + s.e.m. and single data (**c, d**) from *n* = 2 (**a** and **b** for serum) or *n* = 3 (**a-d**) independent experiments. *P* values vs. vehicle control (**c, d**), repeated measures one-way ANOVA of log-transformed data + Dunnett's post hoc test (**c, d**) or Pearson correlation (**b**). Exact *P* values for panels **b, c** and **d** are given in Supplementary Data 4.

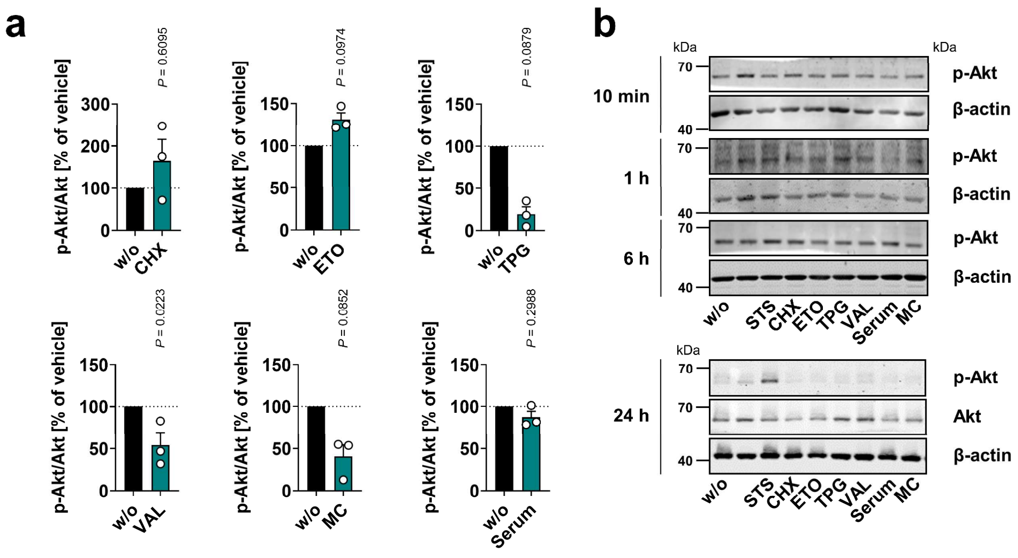

Supplementary Fig. 20 Cytotoxic stress diminishes Akt activation. **a**, **b** Fibroblasts were cultivated under cytotoxic conditions for 48 h (**a**) or as indicated (**b**). **a** Changes in the cellular levels of p-Akt (normalized to Akt) relative to vehicle control. Mean + s.e.m. and single data (**a**) from *n* = 3 independent experiments. *P* values vs. vehicle control (**a**); two-tailed paired student *t*-test. **b** Phosphorylation and expression of Akt. Western blots are representative of three independent experiments. Uncropped Western blots are shown in Supplementary Fig. 42 and 43a.

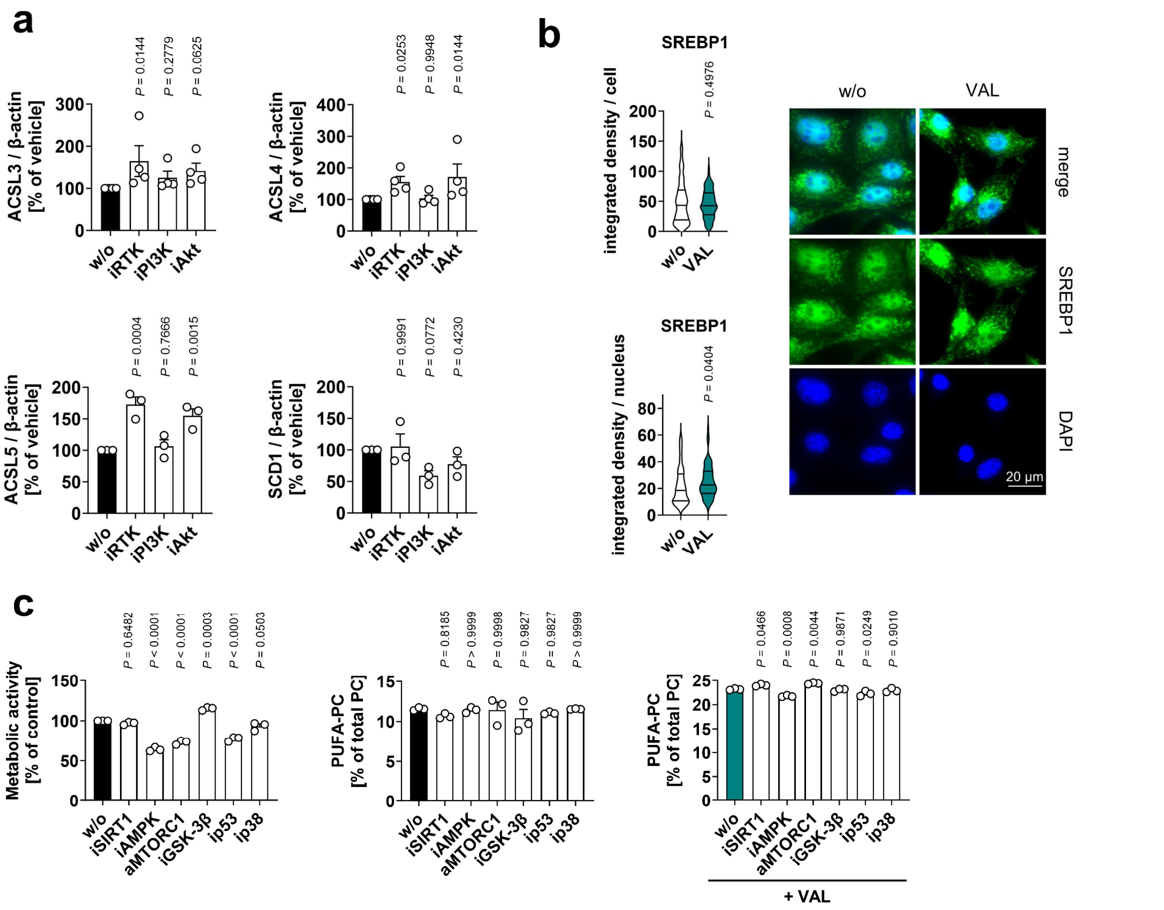

Supplementary Fig. 21 Role of the RTK-PI3K-Akt-SREBP1-axis in controlling lipid metabolic gene expression and the PUFA-PC ratio. **a-c** Fibroblasts were treated with VAL (10 µM), the enzyme inhibitors picropodophyllin (iRTK, 10 µM), LY294002 (iPI3K, 10 µM), ipatasertib (iAkt, 10 µM), selisistat (iSIRT1, 10 µM), BAY3827 (iAMPK, 5 µM), and laduviglusib (iGSK-3β, 5 µM), pifithrin-β (ip53, 10 µM) or Skepinone L (ip38, 1 µM) or the AMPK activator MHY1485 (aMTORC1, 5 µM) for 48 h. **a** Changes in the expression levels of ACSL3, ACSL4, ACSL5, and SCD1 (normalized to β-actin) relative to vehicle control. Uncropped Western blots are shown in Supplementary Fig. 43b. **b** Immunofluorescence images are stained for SREBP1 and nuclei (DAPI). Images are representative of ≥90 single cells analyzed in n = 3 independent experiments (scale bar: 20 µm). Violin plots show the integrated density per cell or per nucleus. **c** Metabolic activity (measured by MTT assay) and cellular proportion of PUFA-PC. Mean + s.e.m and single data (**a**, **c**) or median and quartiles (**b**) from $n = 4$ (**a**, ACSL3, ACSL4, ACSL5) or $n = 3$ (**a,** SCD1, **b**, **c**) independent experiments. P values vs. vehicle control (**a-c**); repeated measures one-way ANOVA of log-transformed data (**a, c**) + Dunnett´s post hoc test and two-tailed unpaired student t-test (**b**). The exact P values of panel **c** are listed in Supplementary Data 4.

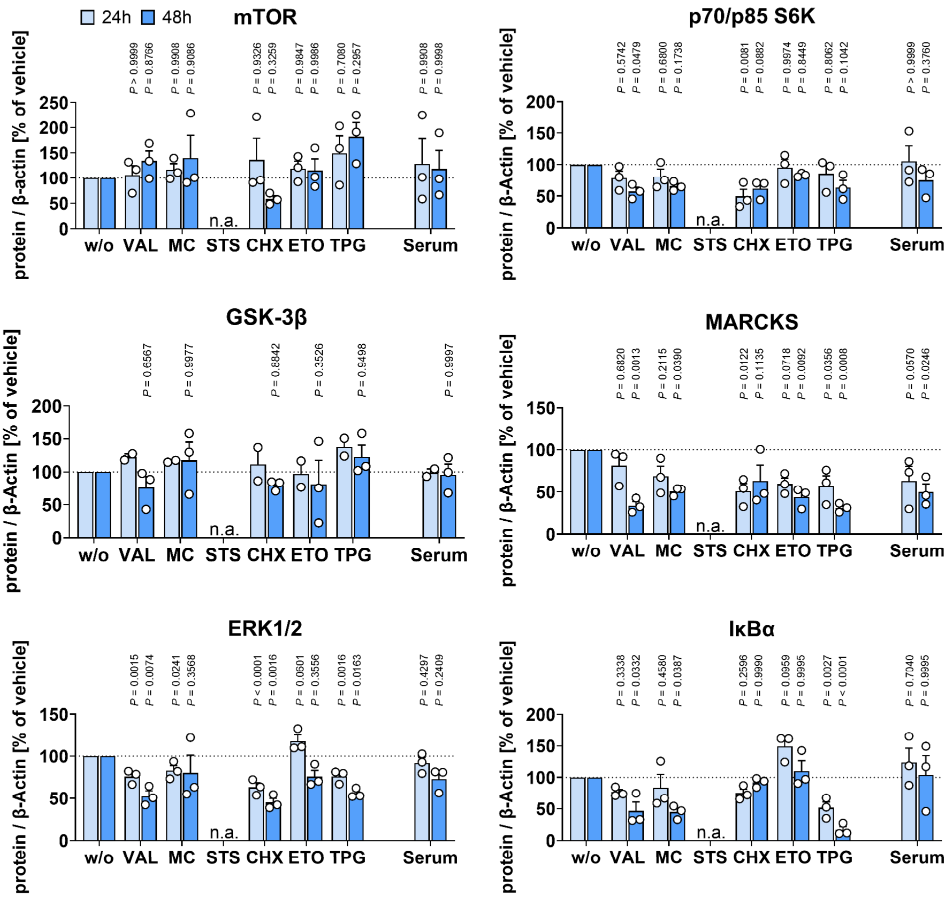

Supplementary Fig. 22 Expression of pro-survival kinases and kinase substrates upon stress. Fibroblasts were cultivated under cytotoxic conditions for 48 h. Total protein expression was determined after 24 and 48 h and normalized to β-actin; n.a., not analyzed. Representative Western blots are shown in Supplementary Figure 23. Mean + s.e.m. and single data from $n = 3$ (except $n = 2$ for GSK-3β, 24 h) independent experiments. P values vs. vehicle control; repeated measures one-way ANOVA of log-transformed data + Dunnett´s post hoc test. Exact P values for mTOR, p70/p85 S6K, ERK1/2 and IκBα are given in Supplementary Data 4.

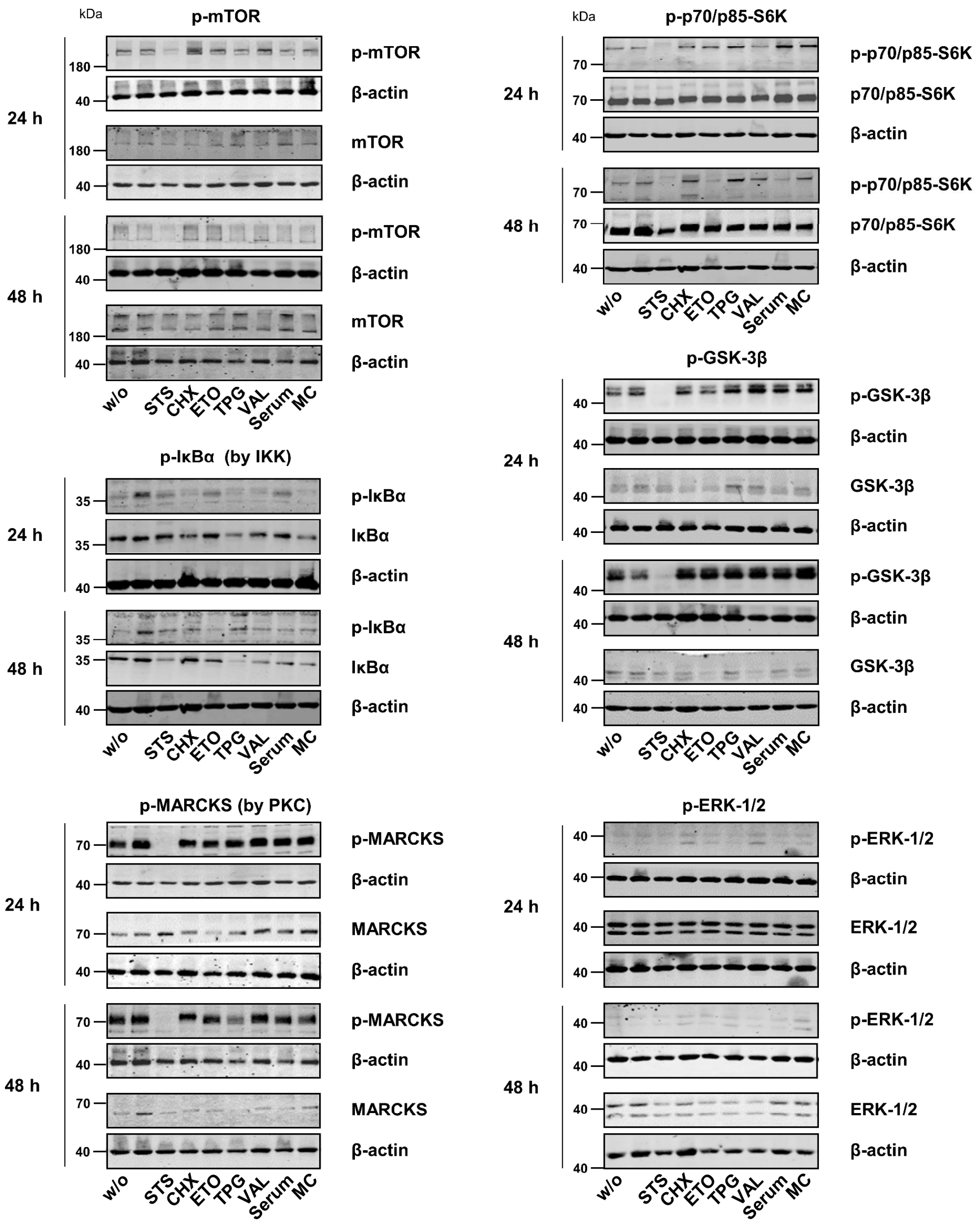

Supplementary Fig. 23 Activation status of kinases with metabolic and pro-survival function. Phosphorylation of kinases or kinase substrates and total protein expression were determined after 24 and 48 h in fibroblasts cultured under cytotoxic conditions. Western Blots are representative of two (p-GSK-3β, 24 h) or three (all other blots) independent experiments. Uncropped Western blots are shown in Supplementary Fig. 44–49.

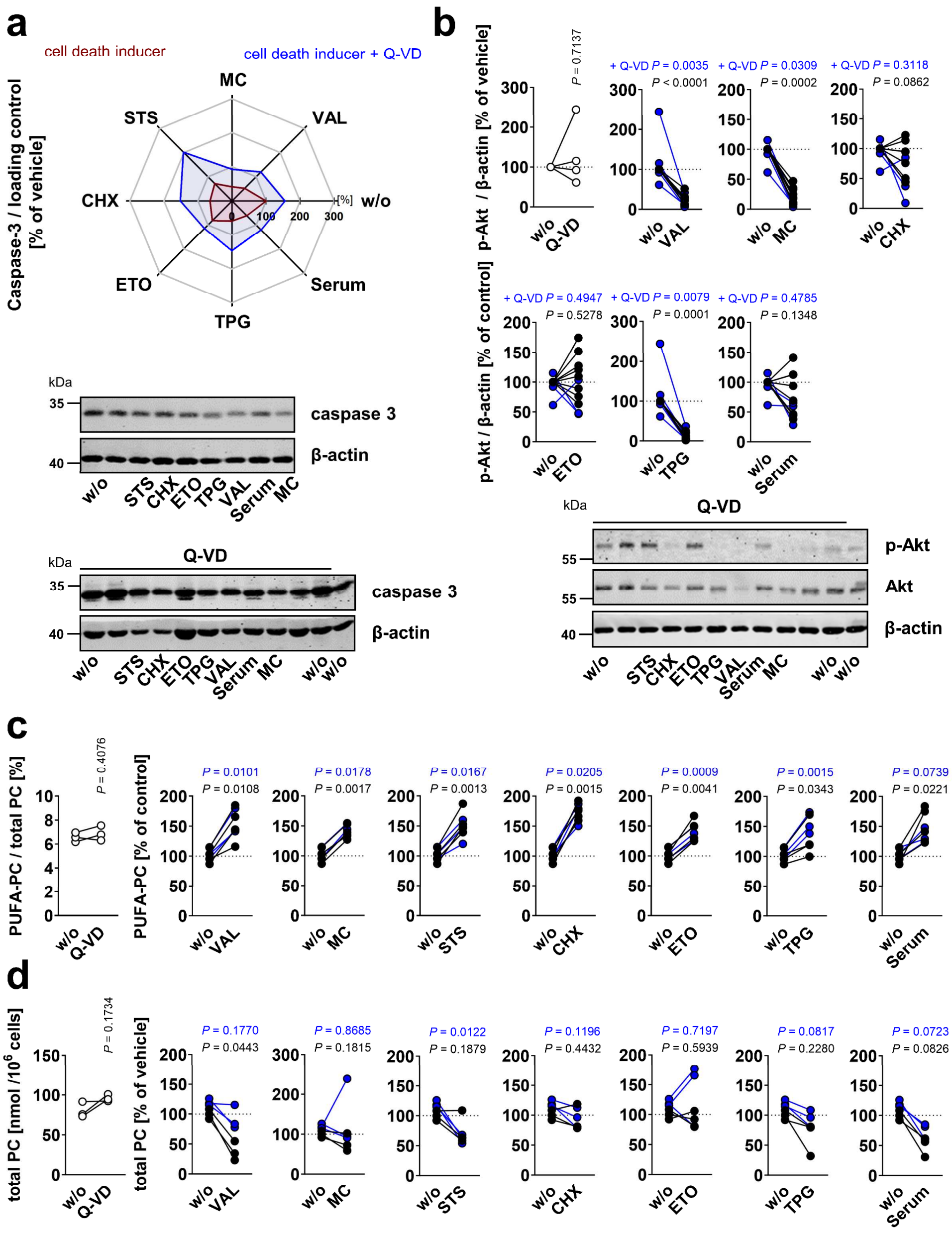

Supplementary Fig. 24 Role of caspases in regulating Akt phosphorylation and PUFA-PC ratios. Fibroblasts were treated with vehicle or the pan-caspase inhibitor Q-VD-OPh (Q-VD; 20 µM) and were exposed to cytotoxic stress for 48 h. **a** Changes of the cellular levels of non-cleaved caspase 3 (normalized to β-actin or GAPDH) relative to vehicle control and representative Western blots for non-cleaved caspase 3 and β-actin. **b-d** Interconnected lines compare data from the same independent experiment. **b** Changes of the cellular levels of p-Akt (normalized to β-actin or GAPDH) relative to vehicle control and representative Western blots for p-Akt, total Akt, and β-actin. **c** Cellular proportion of PUFA-PC. **d** Cellular content of PC. Data of **c** are identical to w/o in **Fig. 2g** and **Supplementary Fig. 12a**. Mean (**a**) or paired data from n = 3 (**a**, **b** for + Q-VD in the MC chart, **c** for the left chart, **c** for + Q-VD in the STS, CHX, ETO, TPG, VAL, Serum, and MC charts, **d**), n = 4 (**b** for the left panel, **b** for + Q-VD, **c** for - Q-VD), n = 8 (**b** for - Q-VD in the STS, CHX, ETO, TPG, VAL, Serum, and MC charts) independent experiments. *P* values vs. vehicle or Q-VD control (**b-d**); two-tailed paired student *t*-test of log-transformed data (**b-d**). Uncropped Western blots are shown in Supplementary Fig. 50. Exact *P* values for panel **b** are given in Supplementary Data 4.

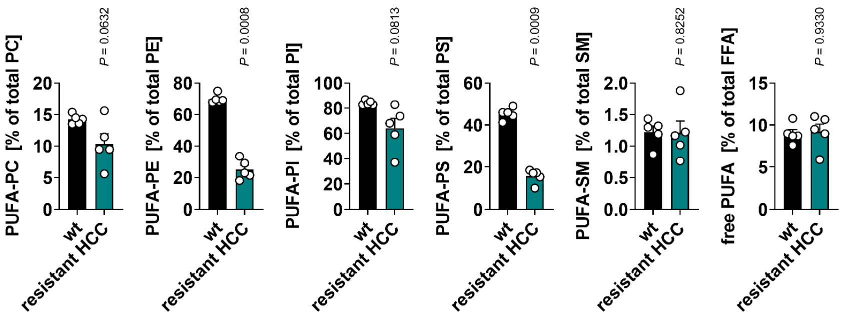

Supplementary Fig. 25 Sorafenib resistance lowers the cellular proportion of PUFAs in phospholipids. Cellular proportion of PUFA-PC, PUFA-PE, PUFA-PI, and PUFA-PS, PUFA-containing sphingomyelins (PUFA-SM), and free PUFAs in wild-type (‘wt’) and sorafenib-resistant Huh-7 hepatocarcinoma cells (‘resistant HCC’). Mean + s.e.m. and single data from n = 5 independent experiments. P values vs. vehicle control; two-tailed paired student t-test of log-transformed data.

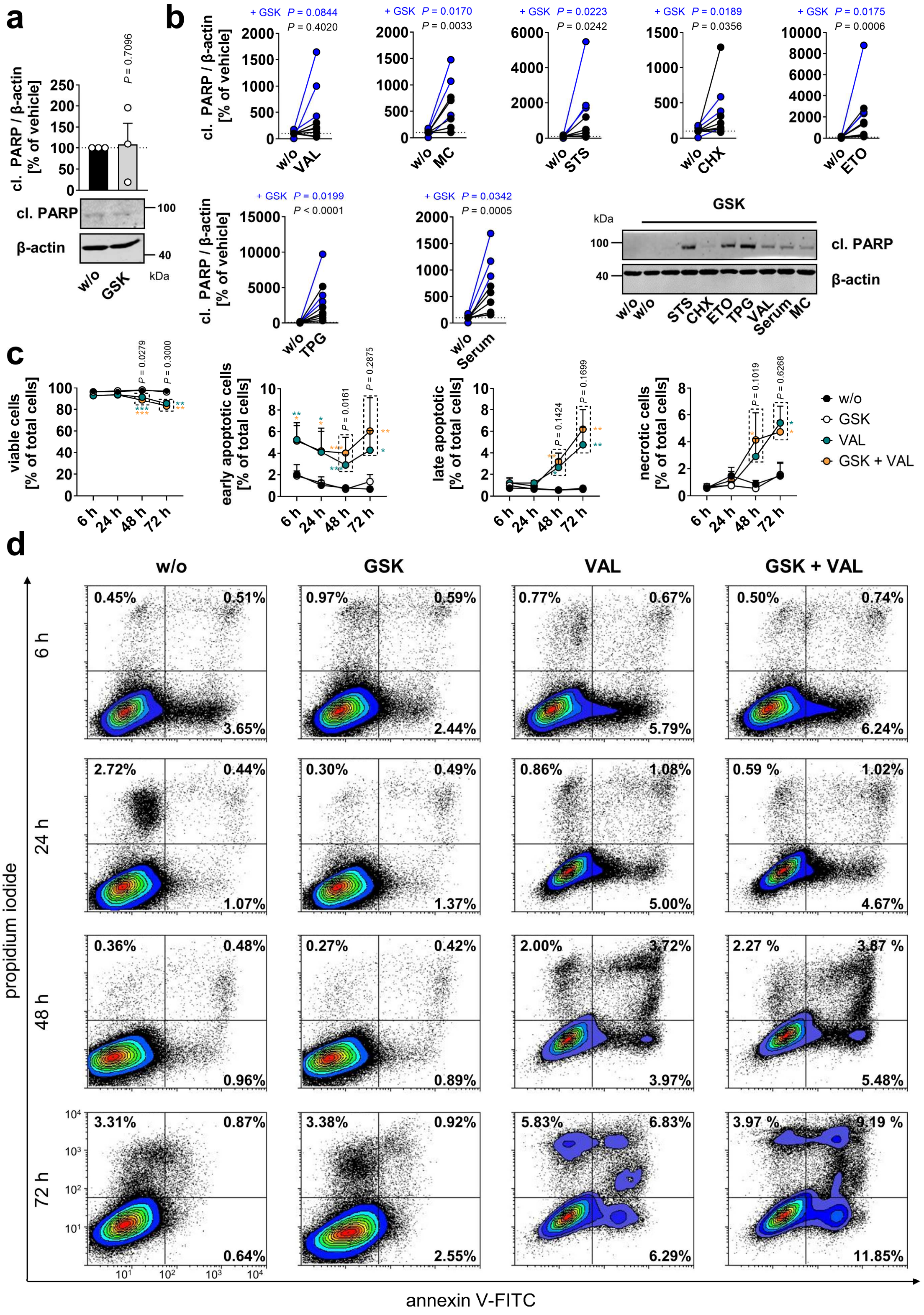

Supplementary Fig. 26 Kinetics of cell death induction by Akt inhibition in stressed and non-stressed cells. Fibroblasts were treated with vehicle or the selective Akt inhibitor GSK690693 (GSK, 1 µM), and exposed to cytotoxic stress. **a**, **b** PARP cleavage normalized to β-actin; cl., cleaved. Western blots are representative of nine (**b** for w/o) or three (**b** for GSK) independent experiments in technical duplicates (**a**). Data of **a**, **b** are identical to w/o in Supplementary Figs. 2a and 31. **c**, **d** Annexin V and propidium iodide (PI) staining. **c** Proportion of annexin V / PI negative cells (viable cells), annexin V

positive / PI negative cells (early apoptotic cells), annexin V positive / PI positive cells (late apoptotic cells), and annexin V negative / PI positive cells (necrotic cells) as percentage of total cells. **d** Cytograms are representative of three independent experiments. Number of cells analyzed: ≥ 25,000. Mean + s.e.m. (**c**) and single data (**a**) or paired data (**b**) from $n = 3$ (**a**, **b**, with GSK, **c**, **d**), $n = 9$ (**b**, without GSK) independent experiments. *$P < 0.05$, **$P < 0.01$, ***$P < 0.001$ vs. vehicle control at the respective timepoint (**c**) or P values vs. vehicle control (**a**, **b**) or as indicated (**c**); two-tailed paired student *t*-test of log-transformed data (**a-c**) or repeated measures one-way ANOVA of log-transformed data + Dunnett´s post hoc test (**c**). Exact P values for panel **b** and **c** are given in Supplementary Data 4. Uncropped Western blots are shown in Supplementary Fig. 51.

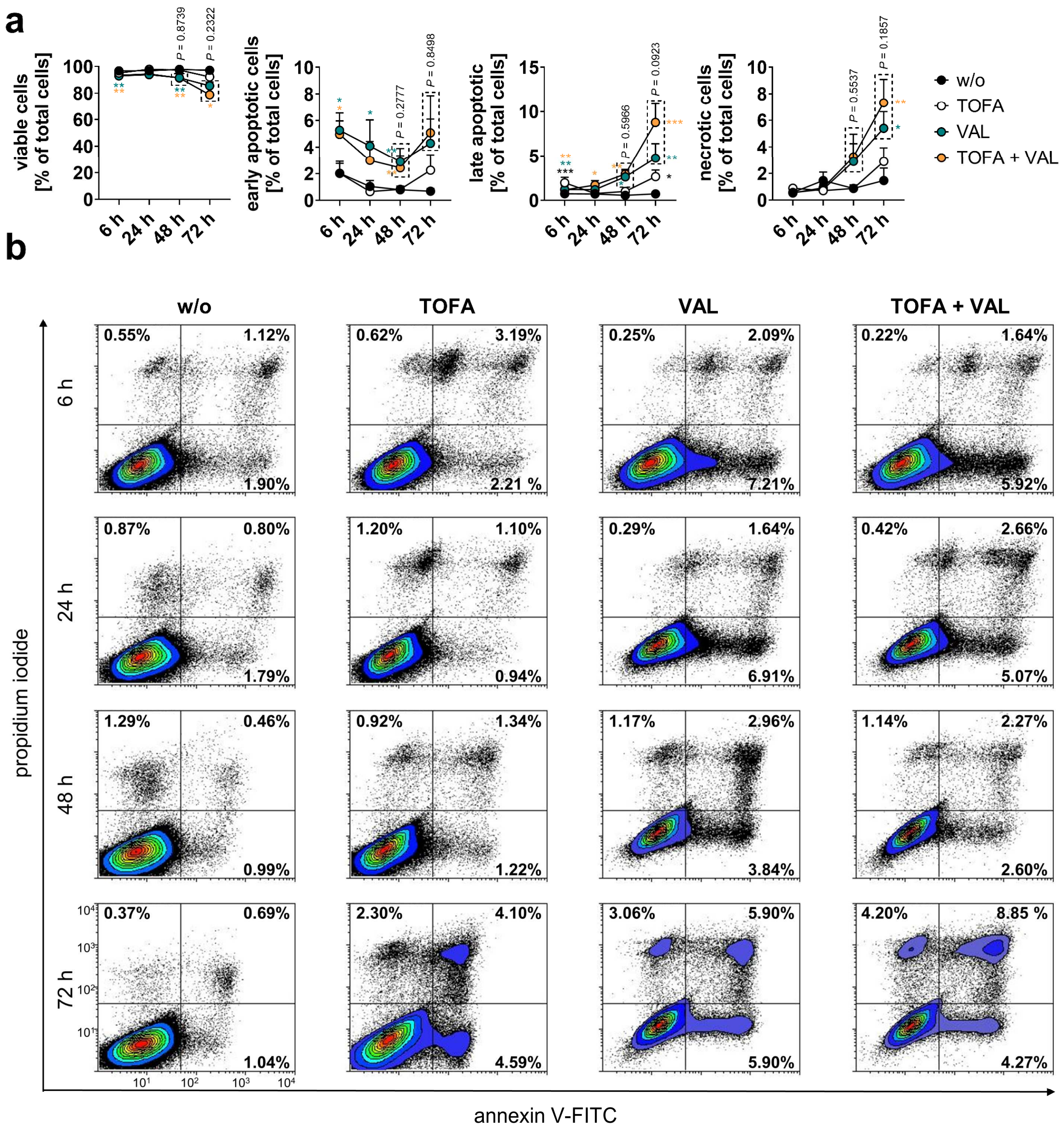

Supplementary Fig. 27 Kinetics of cell death induction by ACC inhibition in stressed and non-stressed cells. Fibroblasts were treated with vehicle, the selective ACC inhibitor TOFA (5 µM), and/or VAL (10 µM) and stained with annexin V and propidium iodide (PI). **a** Proportion of annexin V / PI negative cells (viable cells), annexin V positive / PI negative cells (early apoptotic cells), annexin V positive / PI positive cells (late apoptotic cells), and annexin V negative / PI positive cells (necrotic cells) as percentage of total cells. **b** Cytograms are representative of three independent experiments. Number of cells analyzed: ≥ 25,000. Data shown for w/o and VAL are identical to Supplementary Fig. 26c, d. Mean + s.e.m. (**a**) from $n = 3$ independent experiments. *$P < 0.05$, **$P < 0.01$, ***$P < 0.001$ vs. vehicle control at the respective timepoint or P values as indicated (**a**); two-tailed paired student t-test of log-transformed data (**a**) or repeated measures one-way ANOVA of log-transformed data + Dunnett´s post hoc test (**a**). Exact P values for panel **a** are given in Supplementary Data 4.

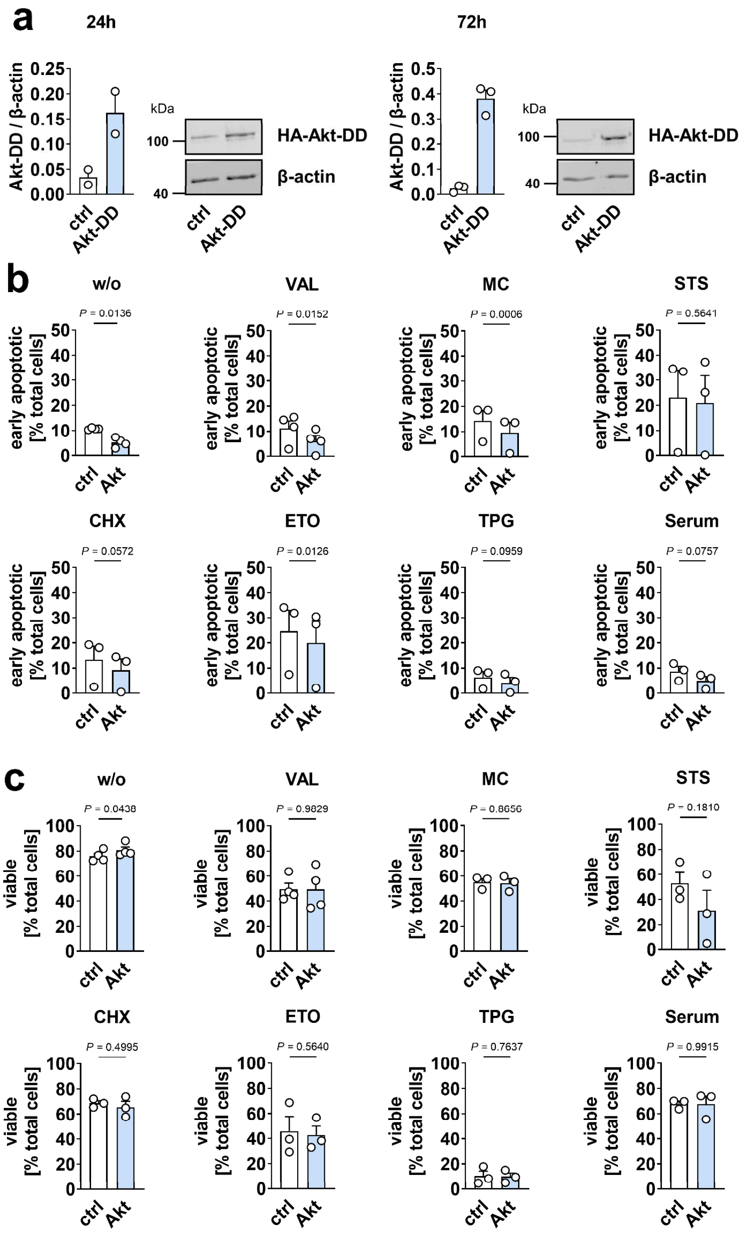

Supplementary Fig. 28 Constitutively active Akt suppresses apoptosis induction in stressed cells. **a** Time-dependent expression of the active Akt-DD mutant (T308D/S473D) (normalized to β-actin) upon transient transfection of fibroblasts. Western blots are representative of two (left panel) or three (right panel) independent experiments. **b**, **c** Annexin V and DRAQ7 staining. Representative cytograms are shown in Supplementary Fig. 29. **b** Proportion of annexin V positive / DRAQ7 negative cells (early apoptotic cells). **c** Proportion of annexin V negative / DRAQ7 negative cells (viable cells). Mean + s.e.m. from n = 2 (**a** for 24 h), n = 3 (**a** for 48 h, **b**, **c**), n = 4 (**b** and **c** for w/o and VAL) independent experiments. P values vs. vehicle control; two-tailed paired student t-test. Uncropped Western blots are shown in Supplementary Fig. 52.

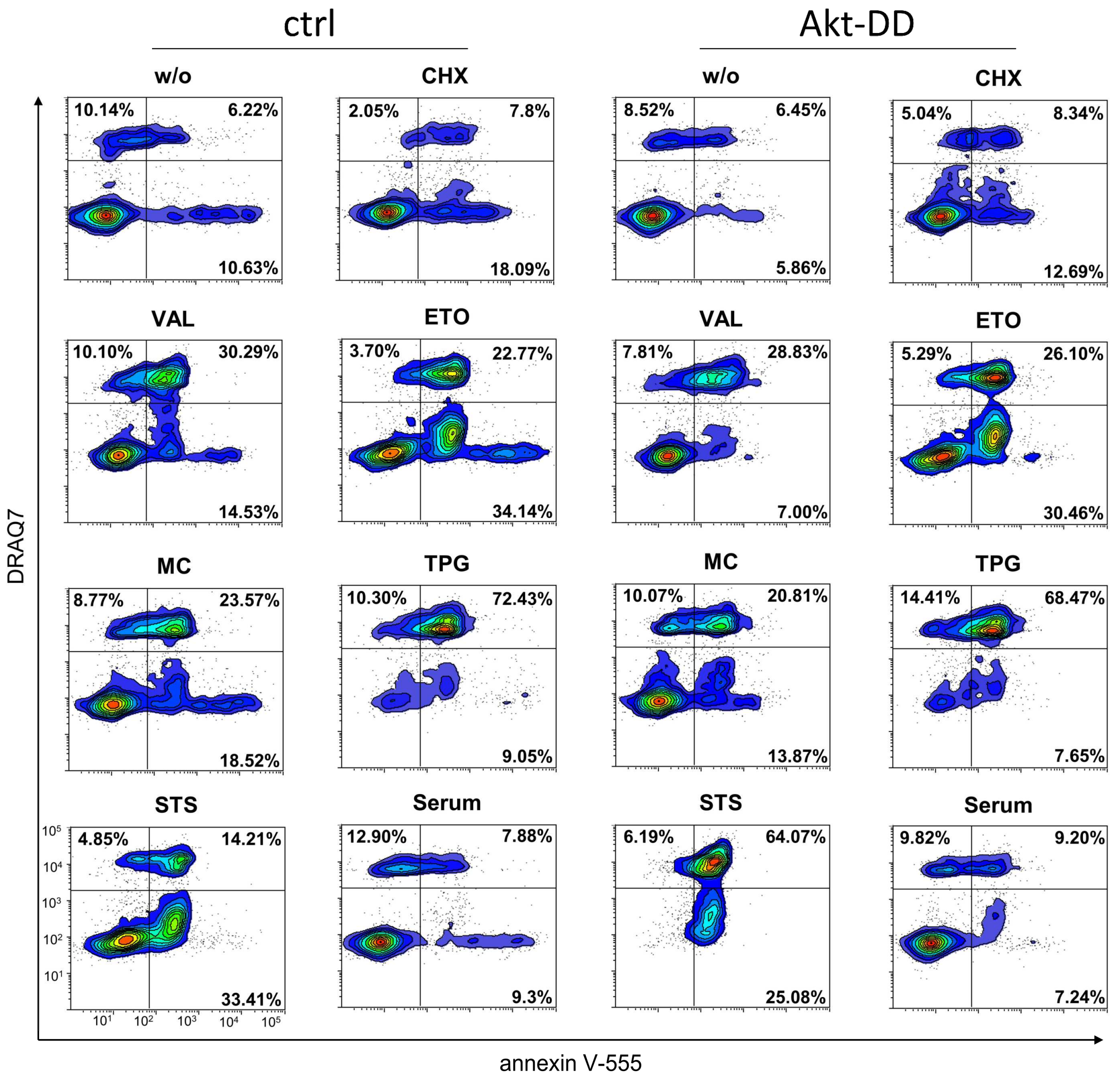

Supplementary Fig. 29 Effect of constitutively active Akt (Akt-DD) on cell death induction in stressed cells. Annexin V-555 and DRAQ7 staining. Cytograms are representative of three or four (w/o and VAL) independent experiments. Number of cells analyzed: ≥ 5,000.

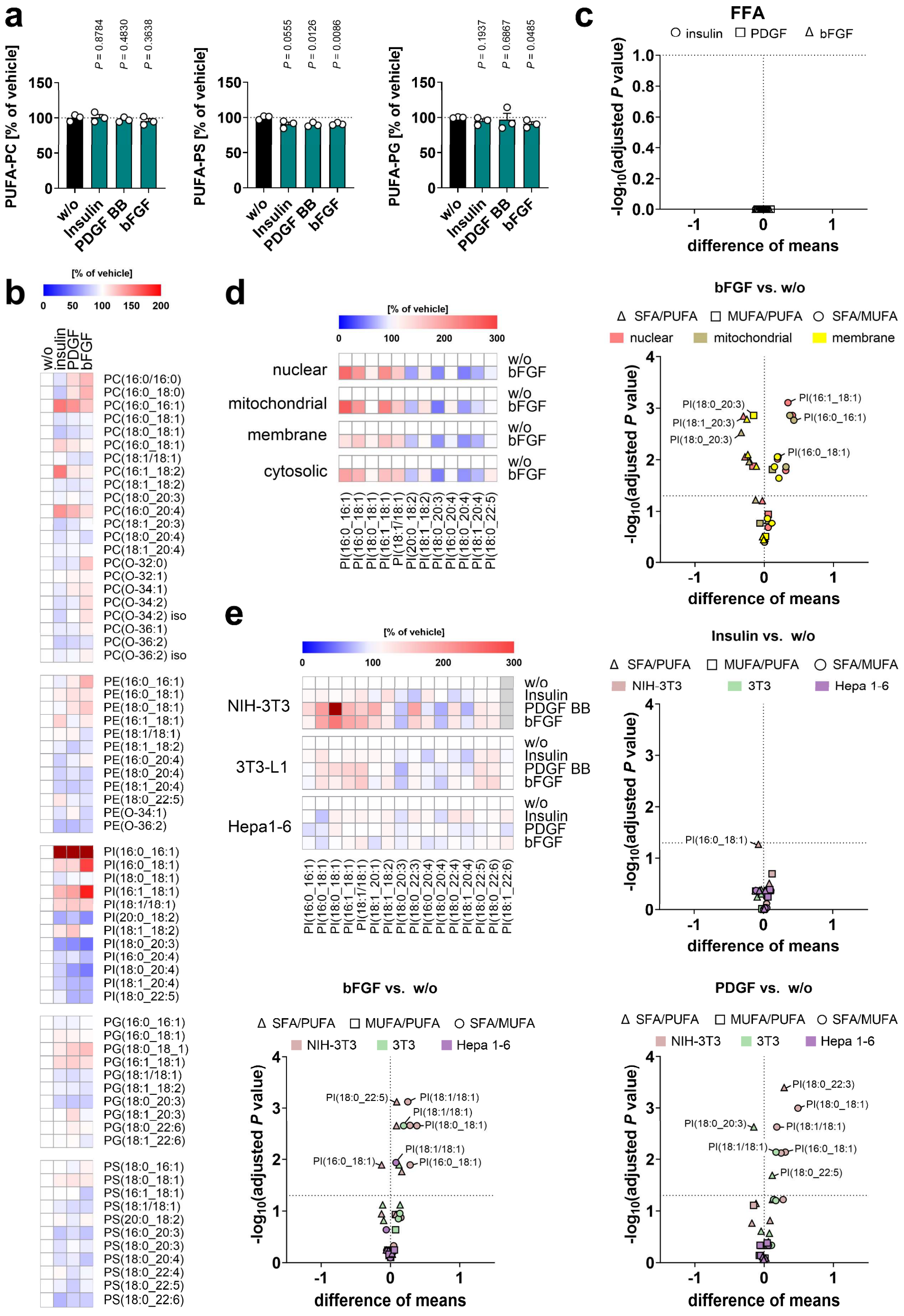

Supplementary Fig. 30 RTK ligands induce a shift from PUFA- to MUFA-containing phospholipids. **a-c** Swiss-3T3 fibroblasts were stimulated with vehicle, insulin (1 µM), PDGF (25 ng/ml), or bFGF (10 ng/ml) for 24 h. **a** Changes in the cellular proportion of PUFA-PC, PUFA-PS, and PUFA-PG relative to vehicle control. **b** Heatmap showing mean percentage changes in the cellular proportion of phospholipids relative to vehicle control. **c** Volcano plot indicating that FFA proportions are neither strongly nor significantly altered by insulin, PDGF, or bFGF. Comparisons show the mean difference of relative intensities and the negative $\log_{10}$(adjusted *P* value). **d** Swiss-3T3 cells were treated with

vehicle or bFGF for 24 h before being subcellularly fractionated by differential centrifugation. The heatmap shows mean percentage changes in the proportion of PI species relative to vehicle control. The volcano plot was calculated as described for panel **c** and indicates nuclear, mitochondrial, and nuclear PI species, whose proportions are strongly and significantly altered. **e** NIH-3T3 fibroblasts, 3T3-L1 preadipocytes, and Hepa1-6 hepatoma cells were stimulated with insulin, PDGF, or bFGF for 24 h. The heatmap shows mean percentage changes in the cellular proportion of PI species relative to vehicle control. Volcano plots were calculated as described for panel **c** and indicate PI species, whose proportions are strongly and significantly altered by insulin, PDGF, or bFGF. Mean (**b-e**) or mean + s.e.m. and single data (**a**) from $n = 3$ (**a-e**; except $n = 2$ for the vehicle control of the cytosolic fraction in panel **d**) independent experiments. P values vs. vehicle control (**a**); two-tailed unpaired student t-test (**a**). Adjusted P values given vs. vehicle control (**c**, **d, e**); two-tailed multiple unpaired student t-tests from log-transformed data with correction for multiple comparisons using a two-stage linear step-up procedure by Benjamini, Krieger, and Yekutieli (false discovery rate 5%) (**c**, **d**, **e**).

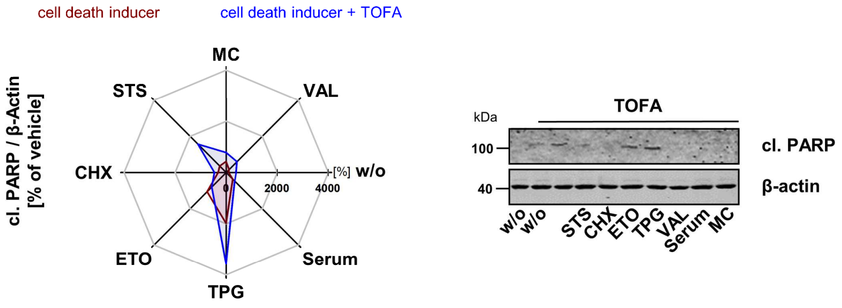

Supplementary Fig. 31 Cytotoxic stress-induced PARP cleavage in cells with active and inactive ACC. Fibroblasts were treated with vehicle or the selective ACC inhibitor TOFA (5 µM) and/or were cultured under cytotoxic conditions for 48 h. Radar chart indicating the percentage changes of PARP cleavage (normalized to β-actin) relative to vehicle control; cl., cleaved. Mean from n = 9 (w/o) or n = 3 (TOFA) independent experiments. Data are identical to w/o in Supplementary Fig. 2a, 26a and 26b. Western blots are representative of three independent experiments. Uncropped Western blots are shown in Supplementary Fig. 53.

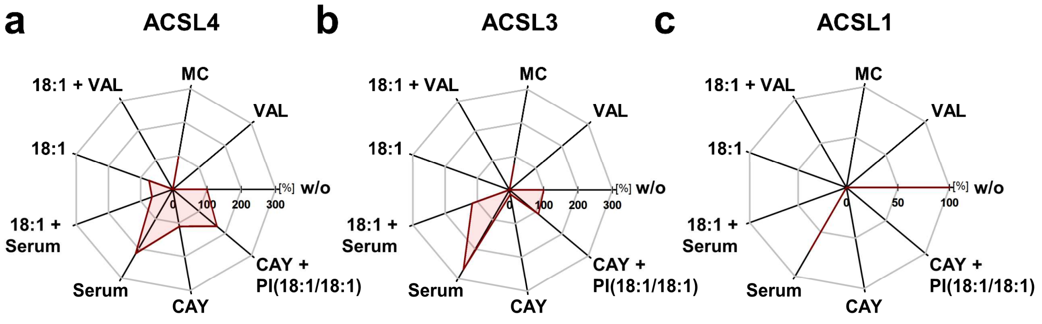

Supplementary Fig. 32 Regulation of the ACSL isoenzyme pattern by cytotoxic stress. Fibroblasts were exposed to cytotoxic stress for 48 h and subjected to quantitative proteomics. **a-c** Radar charts indicate the percentage changes of cellular ACSL4 (**a**), ACSL3 (**b**), and ACSL1 (**c**) levels relative to vehicle control. Mean from n = 3 (except n = 2 for serum) independent experiments.

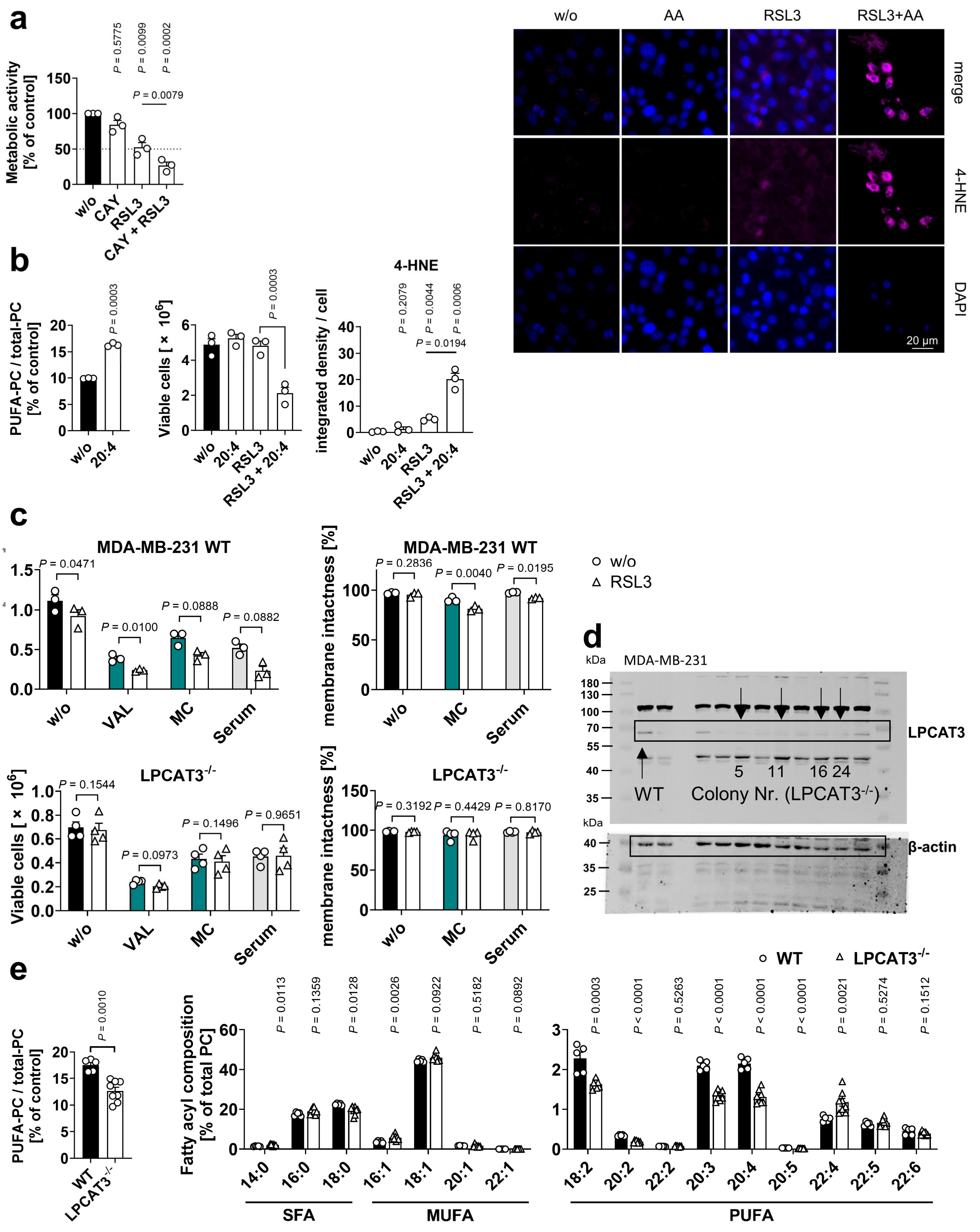

Supplementary Fig. 33 Manipulation of the cellular PUCA-PC ratio affects membrane peroxidation. **a** MDA-MB-231 (shGFP) breast cancer cells were treated with vehicle, RSL3 (1 µM), and/or CAY10566 (CAY, 3 µM) for 48 h. Metabolic activity was measured by MTT assay. **b** NIH-3T3 cells were preincubated with 20:4/arachidonic acid (20 µM) for 24 h and then treated with vehicle or RSL3 (0.5 µM) for 24 h before determination of the PUFA-PC ratio and viable cell numbers and visualization of 4-HNE together with nuclei (DAPI) by immunofluorescence microscopy. Images are representative of ≥ 84 single cells analyzed in *n* = 3 independent experiments (scale bar: 20 µm). The bar chart shows the integrated density per cell. Data for w/o are identical to Fig. 8g. **c** Changes in viable cell number and membrane intactness (trypan blue exclusion) of MDA-MB-231 cells (WT and LPCAT3$^{-/-}$) treated with vehicle, VAL (10 µM), or MC (10 µM), or serum-starved (Serum) for 48 h

and challenged with vehicle or RSL3 (0.1 μM). **d** Uncropped Western blot of LPCAT3 and β-actin showing the deletion of LPCAT3 in MDA-MB-231 cell derived from experiments #5, #11, #16, and #24. **e** PUFA-PC ratio and proportions of individual fatty acids in PC of MDA-MB-231 cells (WT and LPCAT3$^{-/-}$). Mean + s.e.m. and single data (**a-c**, **e**) from *n* = 3 (**a**, **b**, **c**, WT), *n* = 4 (**c**, LPCAT2$^{-/-}$ based on deletions #5, #11, #16 and #24), *n* = 5 (**e**, WT) or *n* = 8 (**e**, LPCAT3$^{-/-}$ based on deletions #5, #11, #16 and #24) independent experiments. *P* values vs. vehicle control (**a**, **b**), as indicated (**a-c**, **e**) or vs. wild type (**e** right panel); repeated measures one-way ANOVA of log-transformed data + Tukey post hoc test (**a**, **b**, viable cells and 4-HNE) or two-tailed unpaired (**e**) or paired (**b**, PUFA-PC, **c**) student *t*-test of log-transformed data.

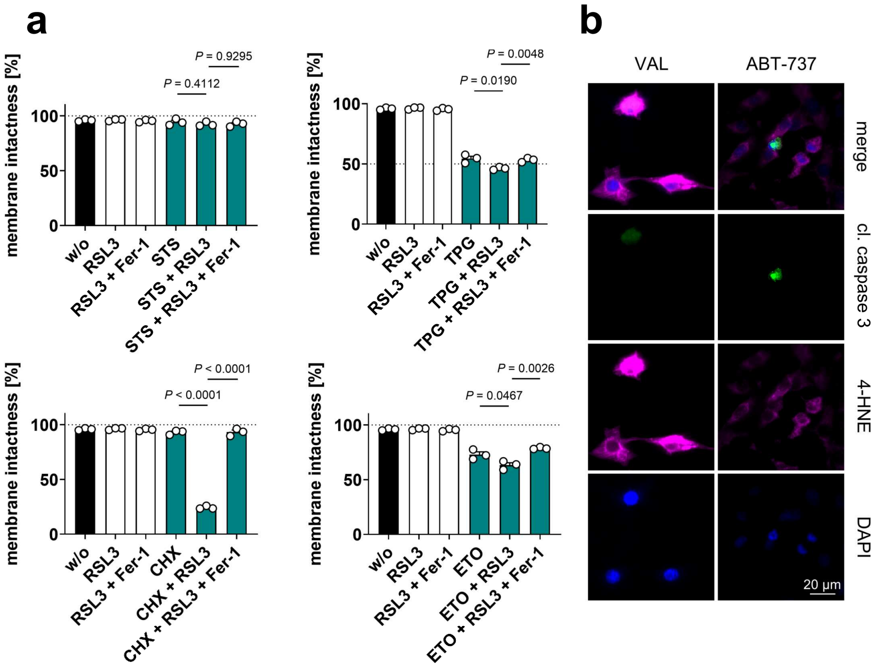

Supplementary Fig. 34 Effect of apoptotic and cytotoxic stress on membrane peroxidation and sensitization to ferroptosis. NIH-3T3 fibroblasts were treated with vehicle, RSL3 (0.05 µM), STS (0.3 µM), TPG (2 µM), CHX (20 µg/ml), ETO (10 µM), Fer-1 (3 µM), VAL (10 µM), or ABT-737 (10 µM) for 48 h. **a** Membrane intactness determined by trypan blue staining. Mean + s.e.m. and single data from *n* = 3 independent experiments. *P* values vs. vehicle control; two-tailed unpaired student *t*-test. Exact *P* values are given in Supplementary Data 4. **b** Immunofluorescence images were stained for cleaved caspase-3, 4-HNE, and nuclei (DAPI). Images are representative of *n* = 3 independent experiments (scale bar: 20 µm).

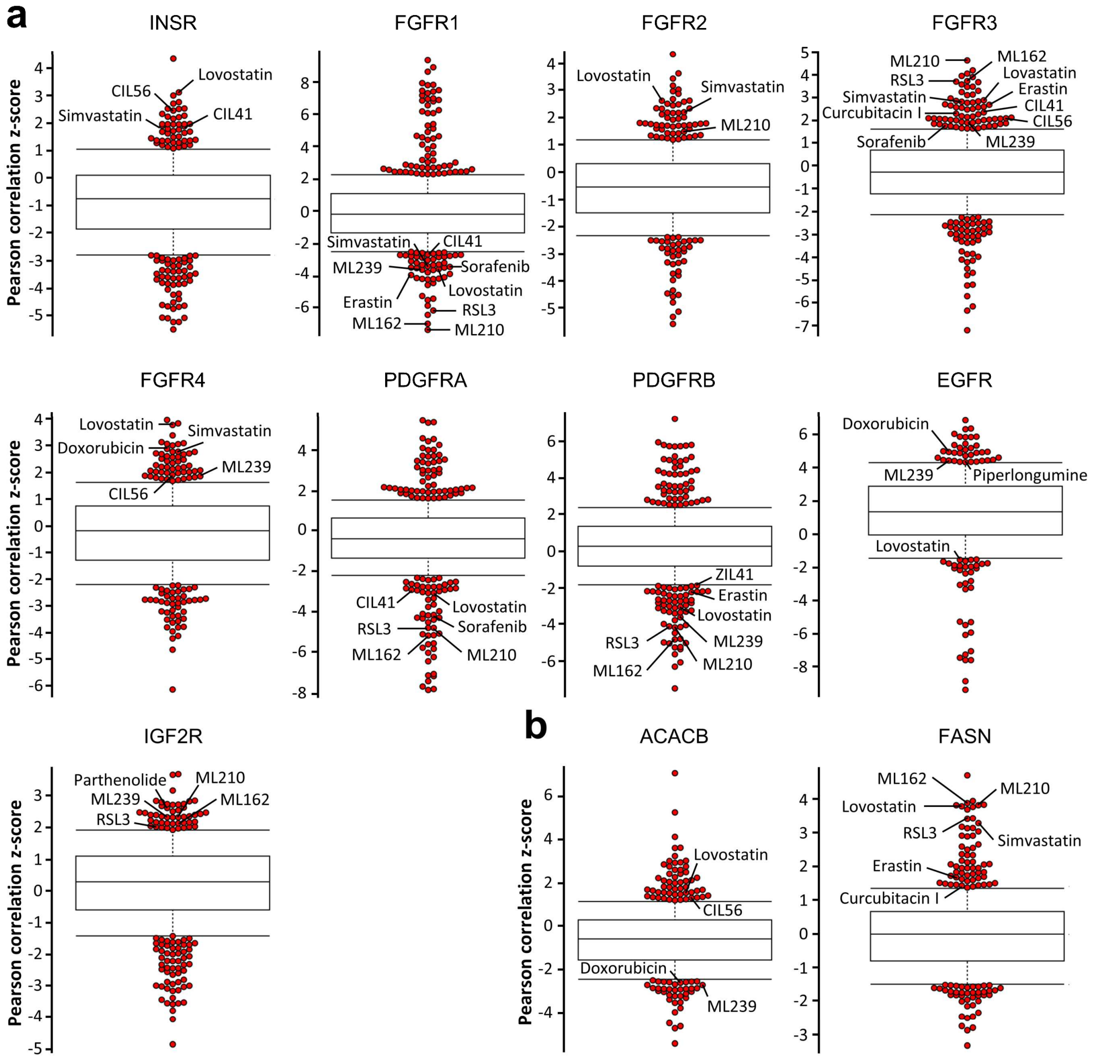

Supplementary Fig. 35 Expression of RTKs and fatty acid biosynthetic enzymes in correlation with ferroptosis resistance. **a**, **b** Pearson correlation Z-scores for each compound inform about the correlation of cancer cell resistance with the expression of insulin and growth factor receptors (**a**) as well as ACACB (ACC2) and FASN (**b**). Number of cancer cell lines: 824; number of small molecules: 481. Data were extracted from the Cancer Therapeutics Response Portal and are presented in box plots with an interquartile multiplier of 0.5.

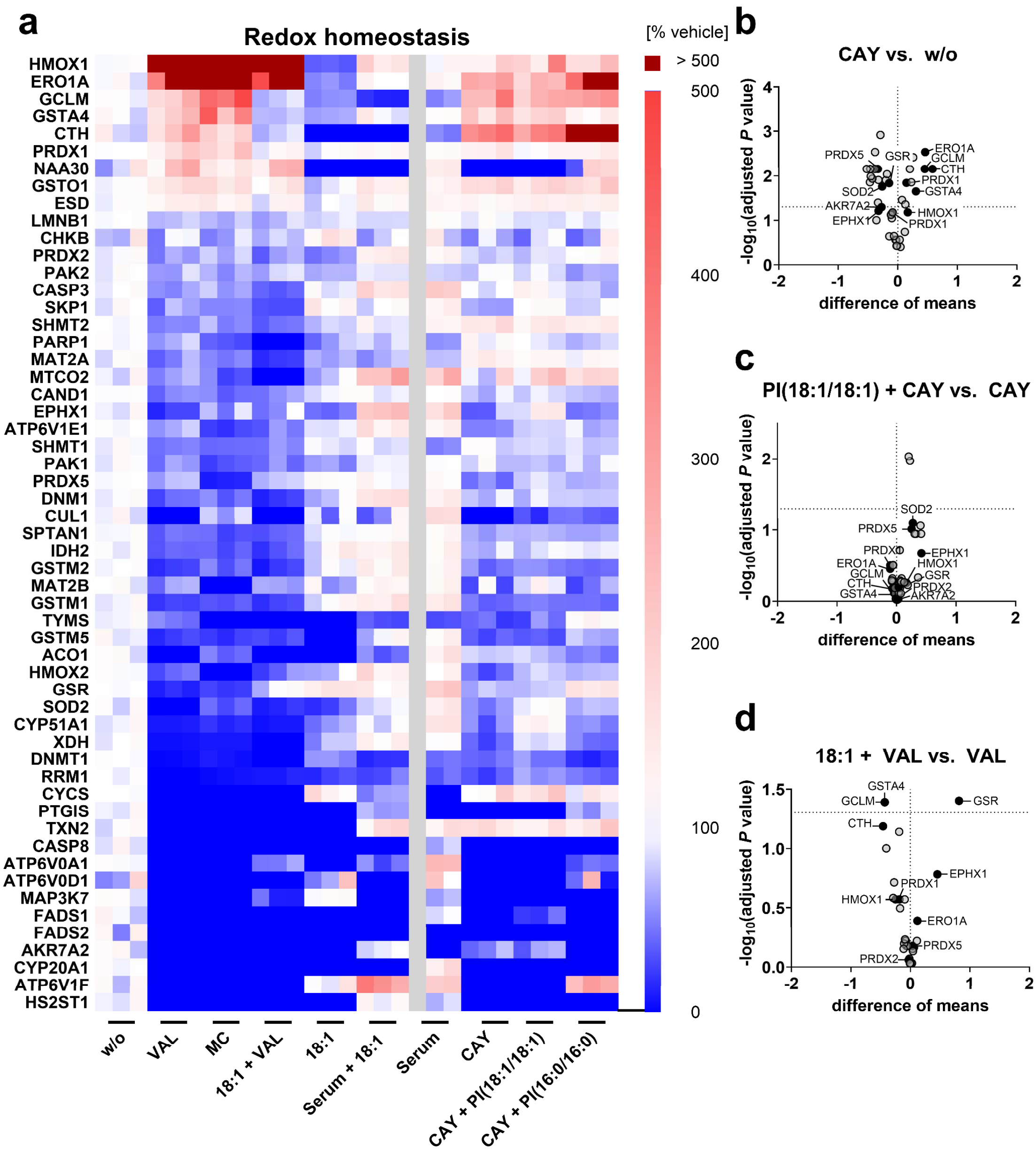

Supplementary Fig. 36 Regulation of proteins in redox homeostasis at the proteome level. Fibroblasts were treated with vehicle, VAL (10 µM), MC (10 µM), 18:1 (100 µM), VAL (10 µM) plus 18:1 (100 µM), CAY10566 (CAY, 3 µM), or CAY (3 µM) plus PI(18:1/18:1) (50 µM) or the saturated control PI(16:0/16:0) (50 µM), or were serum starved (serum) in presence or absence of 18:1 (100 µM) for 48 h. Quantitative proteomics focused on proteins that contribute to redox homeostasis (and are either related to ROS regulation, antioxidant function, glutathione metabolism, iron metabolism, NRF2 signaling, or ferroptosis) and are up- or downregulated by VAL and MC in the same direction (≥ 20%). **a** Heatmap showing relative changes in protein levels. Single data of independent experiments (n = 3, except n = 2 for serum depletion) were calculated as percentage of vehicle control. **b-d** Volcano plots highlight proteins that are regulated by CAY compared to vehicle control (**b**) or whose regulation by CAY (**c**) and VAL (**d**) is compensated by PI(18:1/18:1) and 18:1, respectively. Comparisons of the indicated treatment groups show the difference of mean absolute intensities of $\log_{10}$ data and the negative $\log_{10}$(adjusted P value) from three independent experiments. Adjusted P values given vs. vehicle control; two-tailed multiple unpaired student t-tests from log-transformed data with correction for multiple comparisons using a two-stage linear step-up procedure by Benjamini, Krieger, and Yekutieli (false discovery rate 5%).

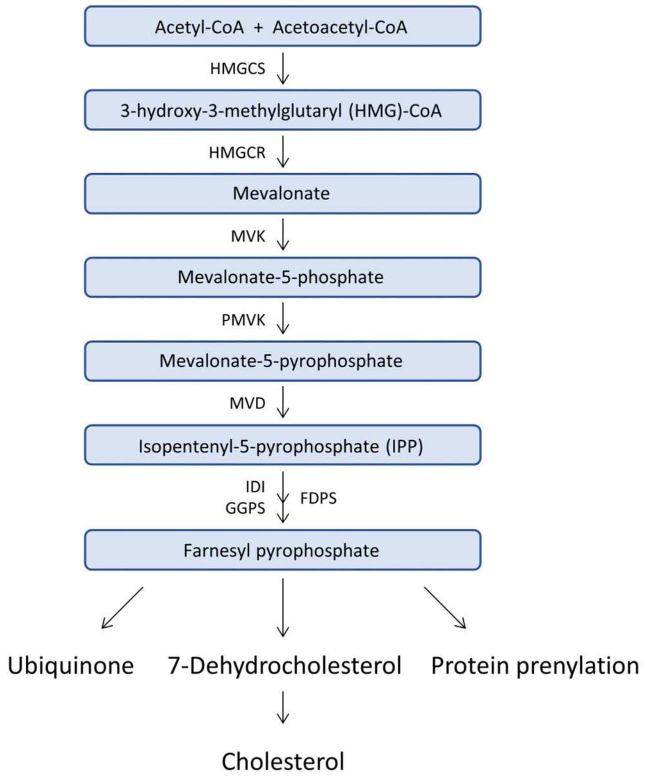

Supplementary Fig. 37 Biosynthesis of isoprenoids via the mevalonate pathway. FDPS, farnesyl diphosphate synthase; GGPS, geranylgeranyl diphosphate synthase; HMGCR, HMG-CoA reductase; IDI, isopentenyl-diphosphate delta isomerase.

Supplementary Fig. 2a

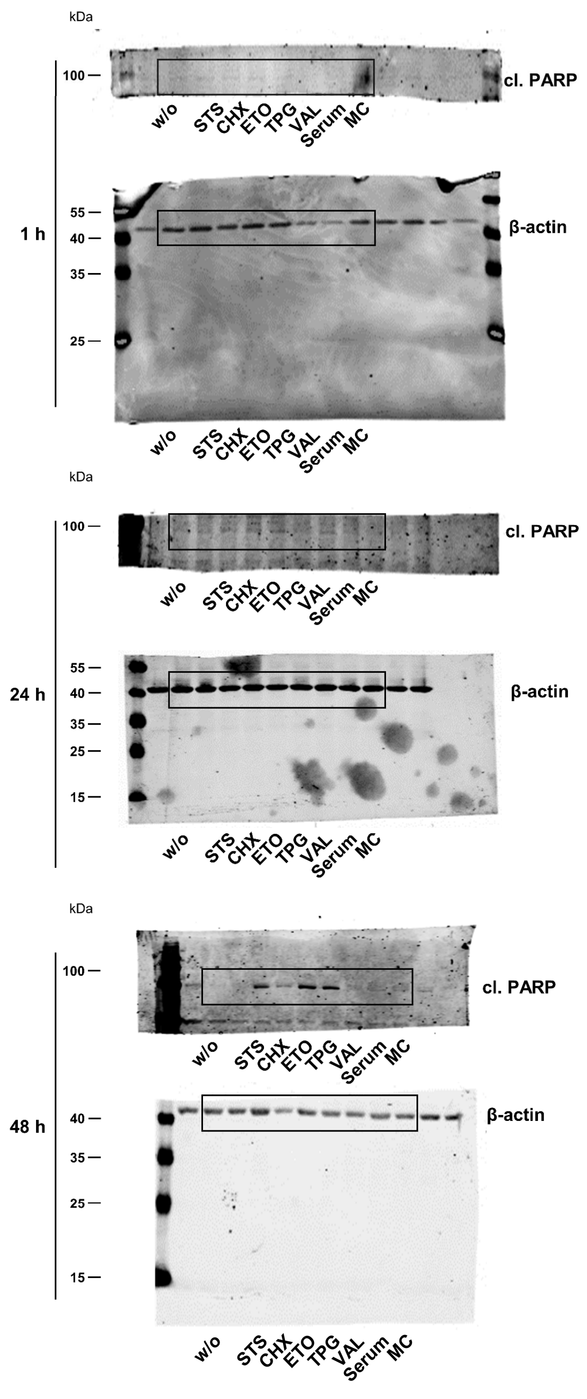

Supplementary Fig. 38 Uncropped versions of the Western Blots presented in Supplementary Fig. 2a.

Supplementary Fig. 8

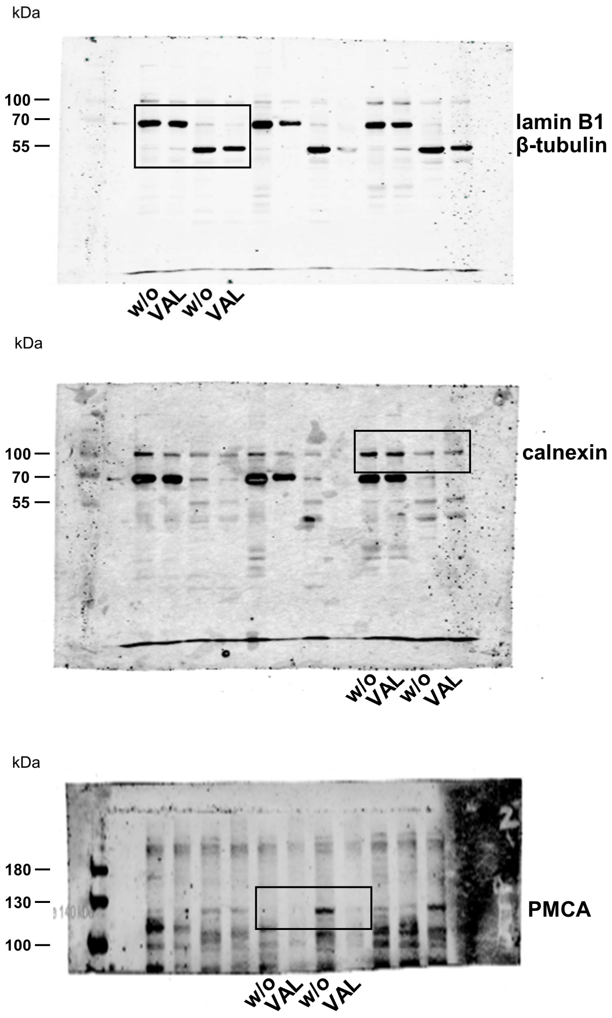

Supplementary Fig. 39 Uncropped versions of the Western Blots presented in Supplementary Fig. 8.

Supplementary Fig. 14a

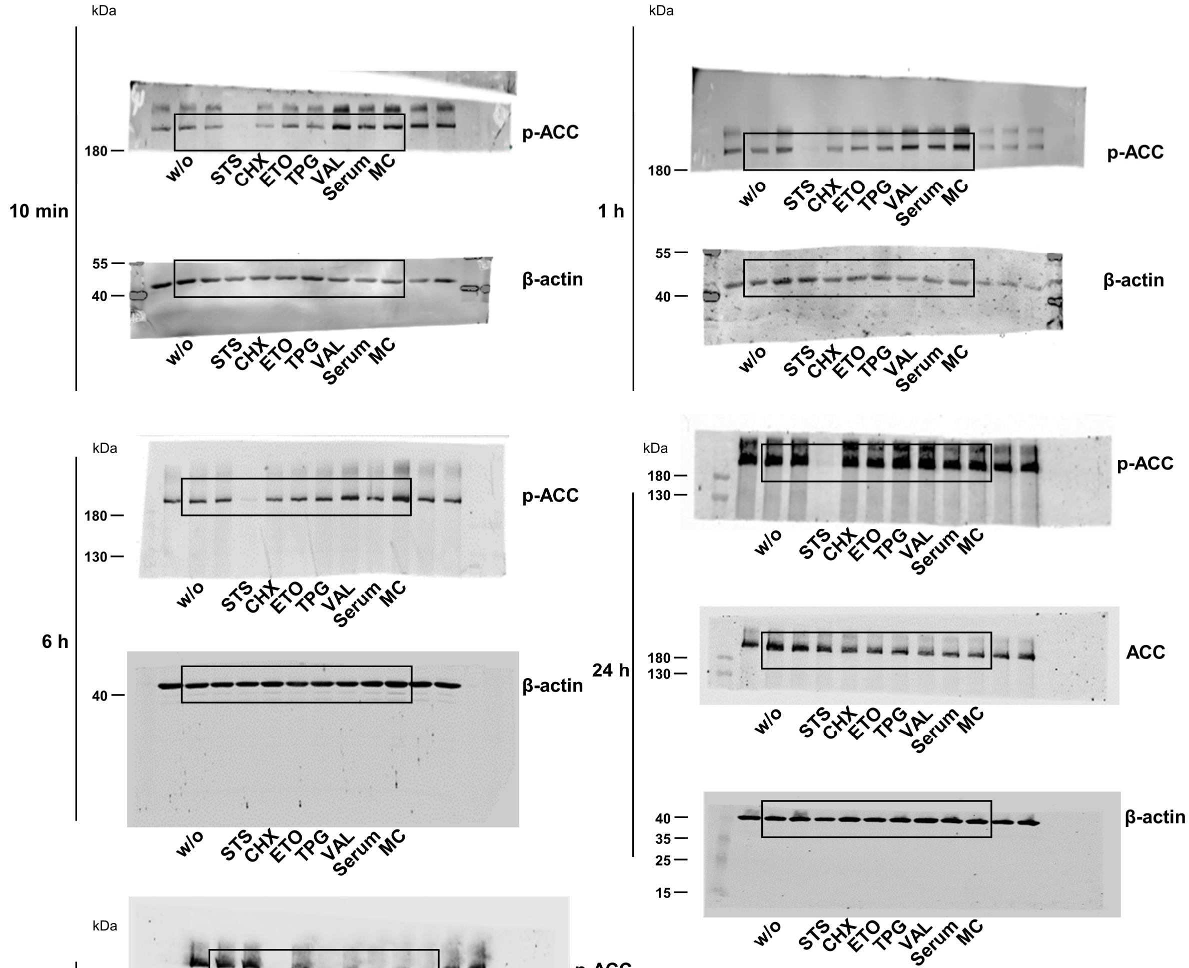

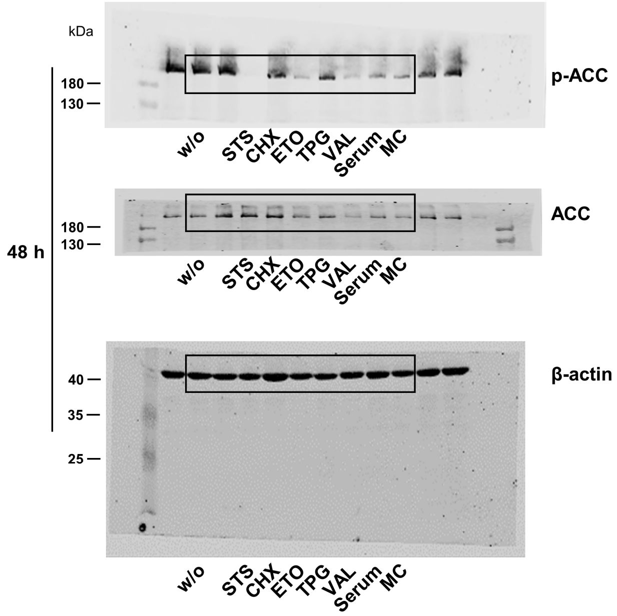

Supplementary Fig. 14b

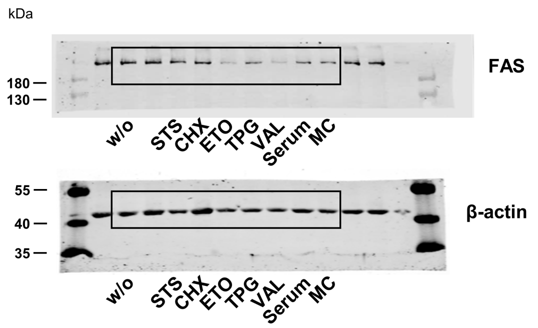

Supplementary Fig. 40 Uncropped versions of the Western Blots presented in Supplementary Fig. 14a and b. The blots for the loading control β-actin at 10 min and 1 h are identical to Thürmer et al.[2].

Supplementary Fig. 14c

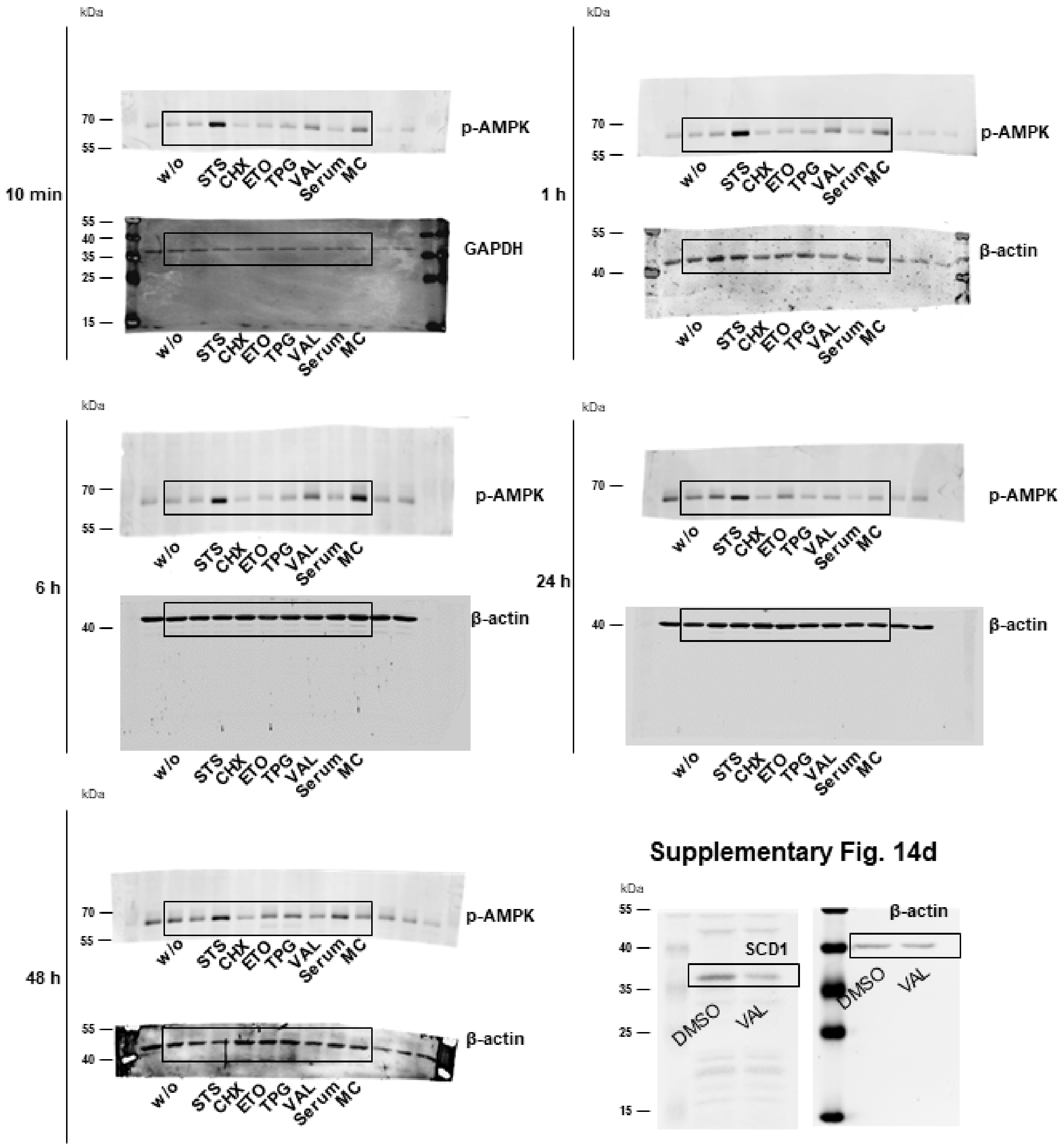

Supplementary Fig. 41 Uncropped versions of the Western Blots presented in Supplementary Fig. 14c and d. The blot for the loading control β-actin 1 h is also shown by Thürmer et al.[2]. The blots for the loading control β-actin 1 h and 6 h are identical to Supplementary Fig. 14a and Supplementary Fig. 40.

Supplementary Fig. 20

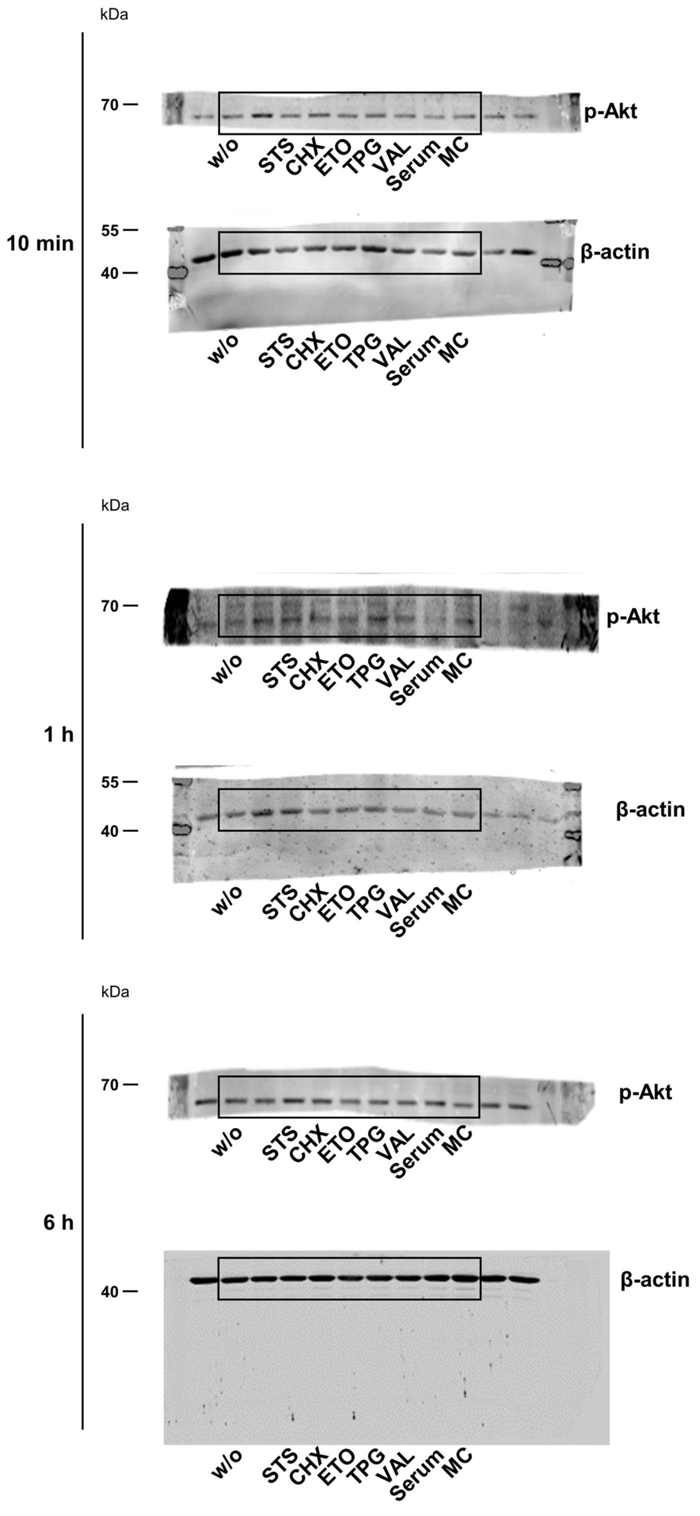

Supplementary Fig. 42 Uncropped versions of the Western Blots (10 min, 1 h, 6 h) presented in Supplementary Fig. 20. The blots for the loading control β-actin at 10 min and 1 h are also shown by Thürmer et al.[2]. The blots for the loading control β-actin at 10 min, 1 h and 6 h are identical to Supplementary Fig. 14a, c and Supplementary Fig. 40 and 41.

a Supplementary Fig. 20

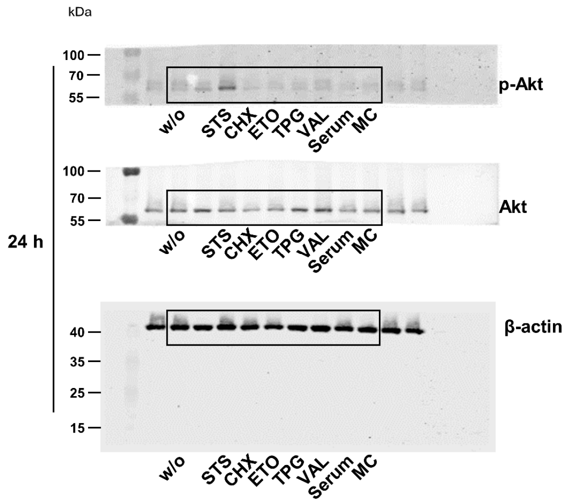

b Supplementary Fig. 21

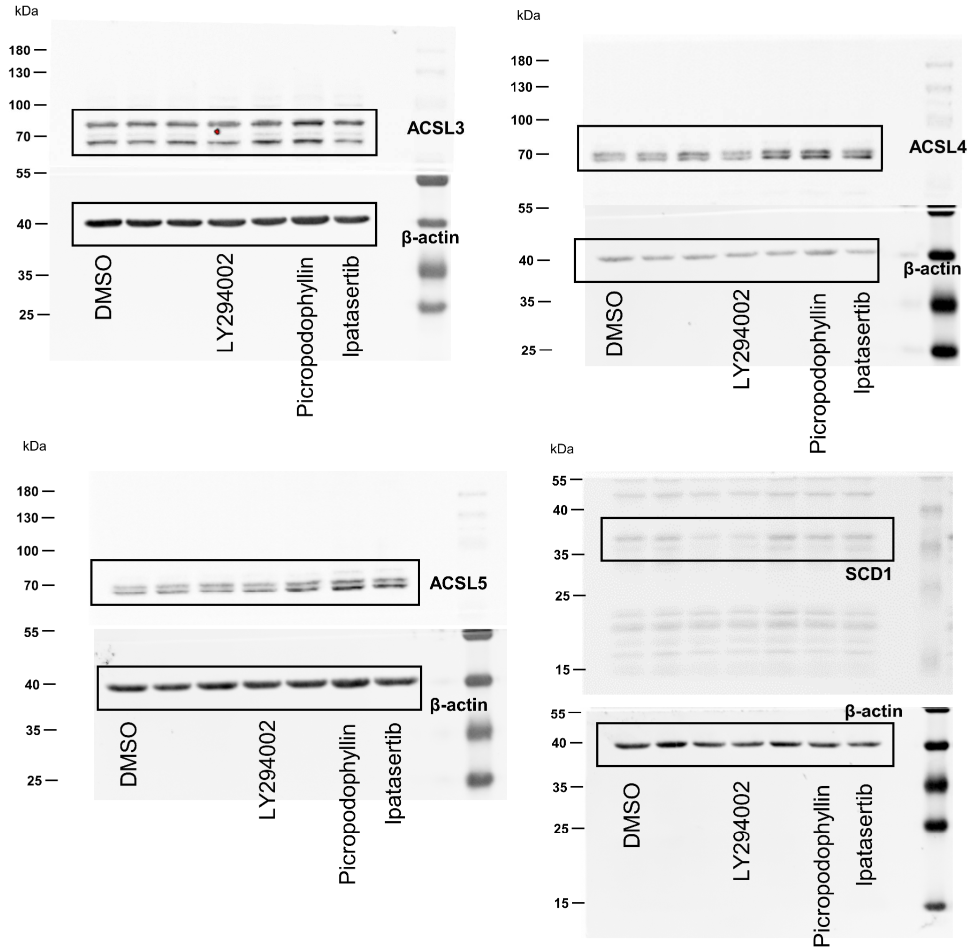

Supplementary Fig. 43 Uncropped versions of the Western Blots (24 h) presented in Supplementary Fig. 20 (**a**) and 21 (**b**).

Supplementary Fig. 23

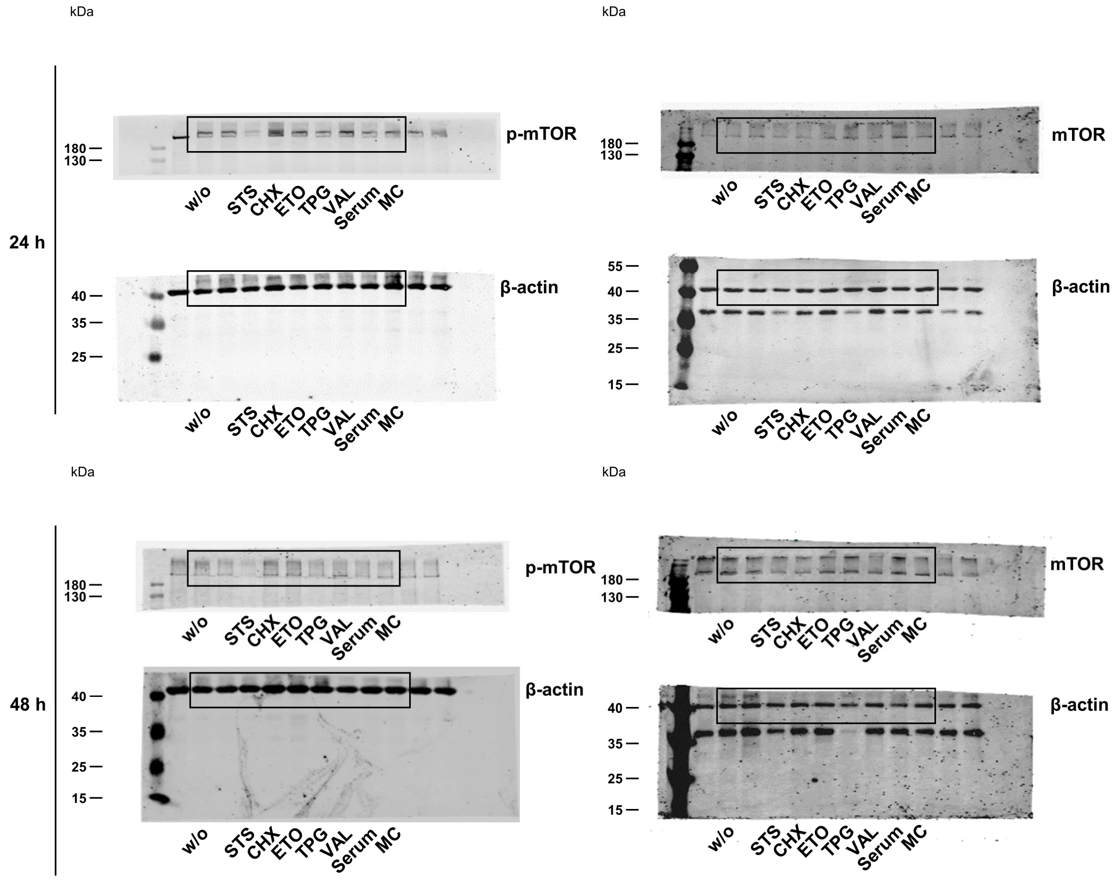

Supplementary Fig. 44 Uncropped versions of the Western Blots (p-mTOR and mTOR) presented in Supplementary Fig. 23.

Supplementary Fig. 23

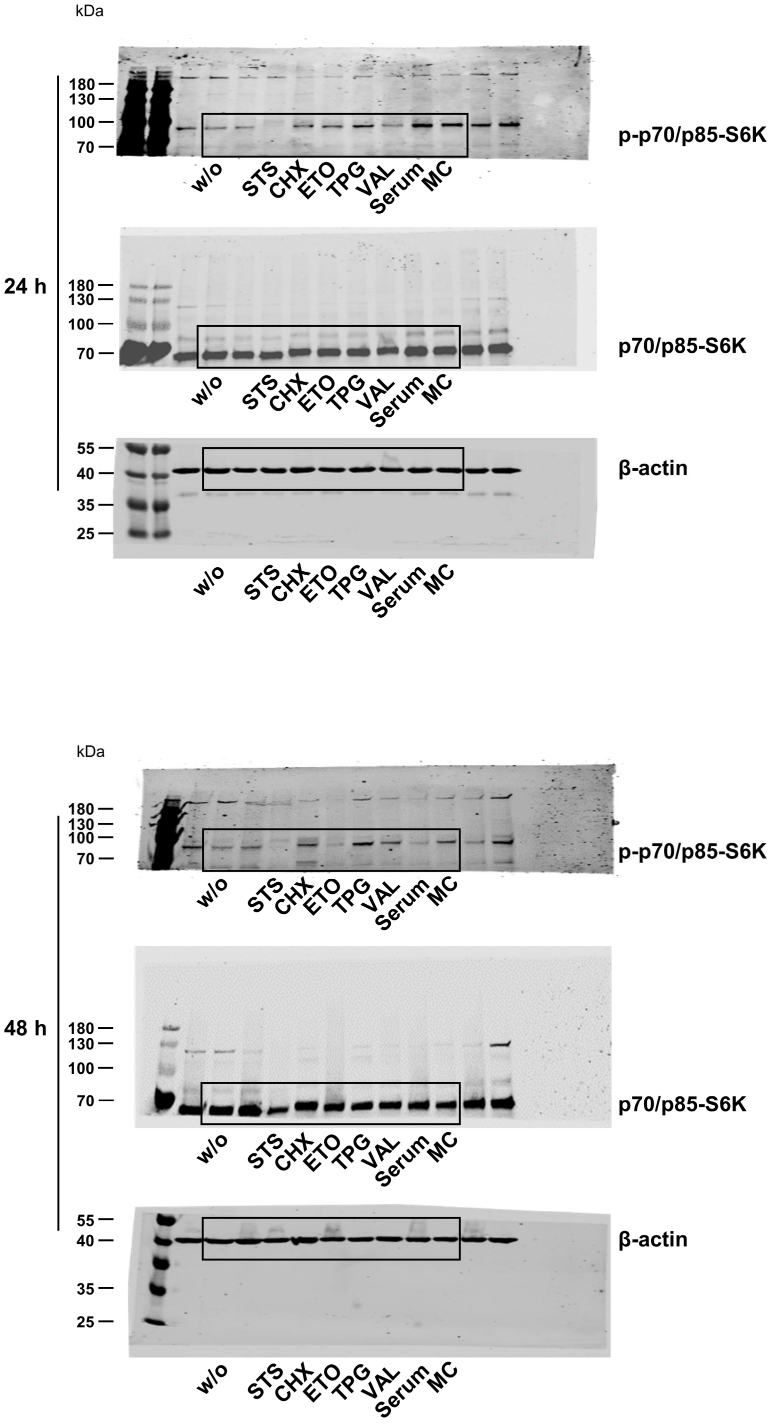

Supplementary Fig. 45 Uncropped versions of the Western Blots (p-p70/p85-S6K and p70/p85-S6K) presented in Supplementary Fig. 23. The blot for the loading control β-actin at 24 h is identical to Thürmer et al.[2].

Supplementary Fig. 23

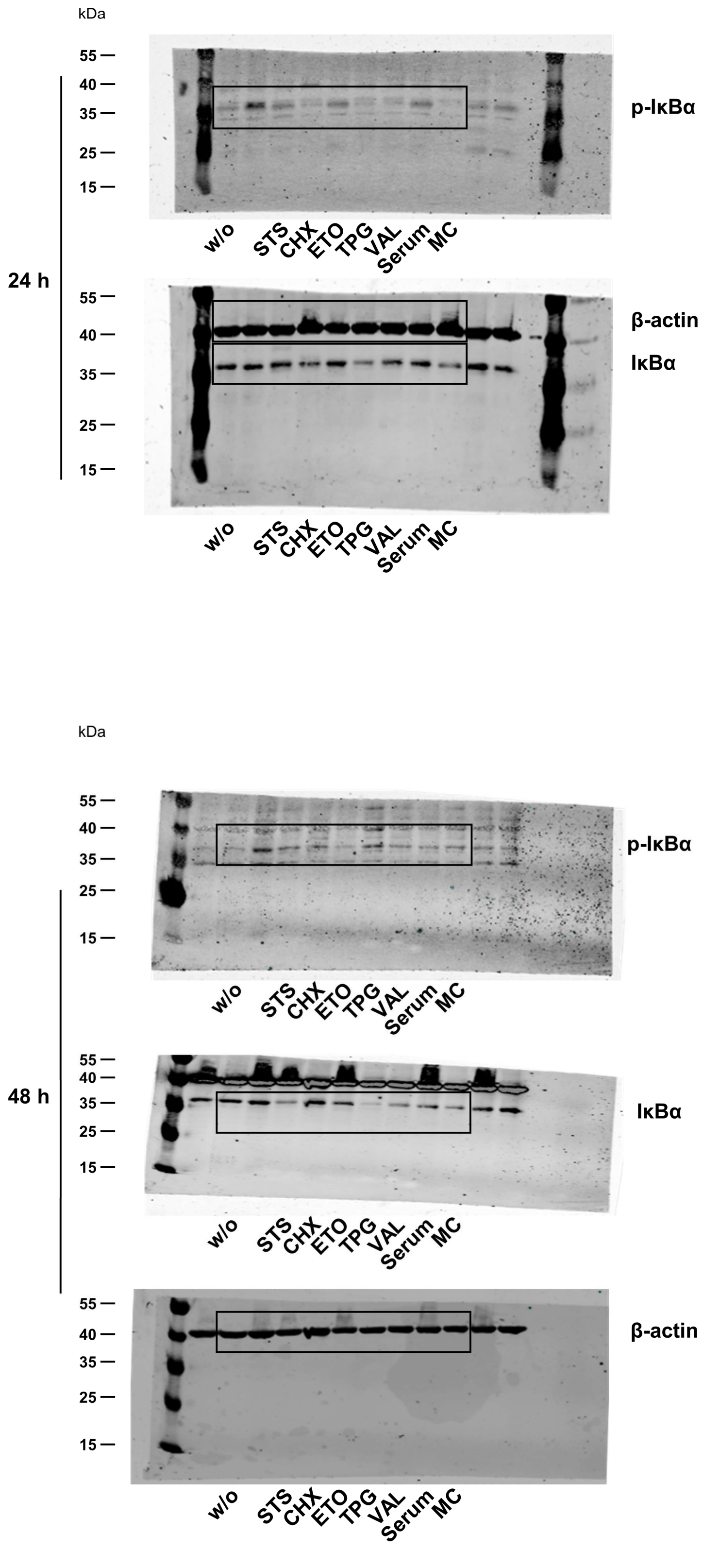

Supplementary Fig. 46 Uncropped versions of the Western Blots (p-IκB and IκB) presented in Supplementary Fig. 23.

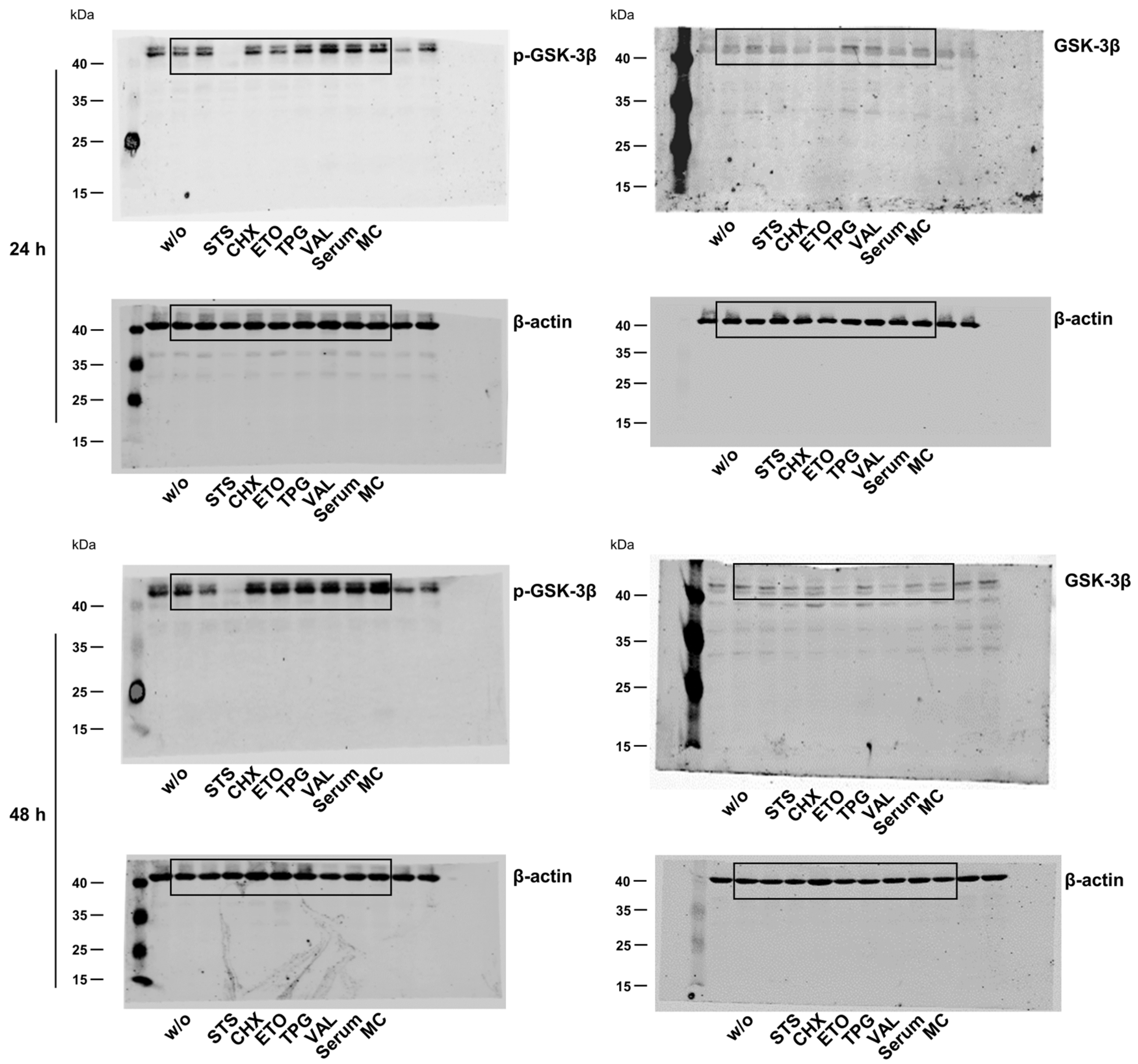

Supplementary Fig. 47 Uncropped versions of the Western Blots (p-GSK-3β and GSK-3β) presented in Supplementary Fig. 23. The blot for the loading control β-actin shown for p-GSK3β at 48 h is identical to Supplementary Fig. 23 (p-mTOR) and Supplementary Fig. 44.

Supplementary Fig. 23

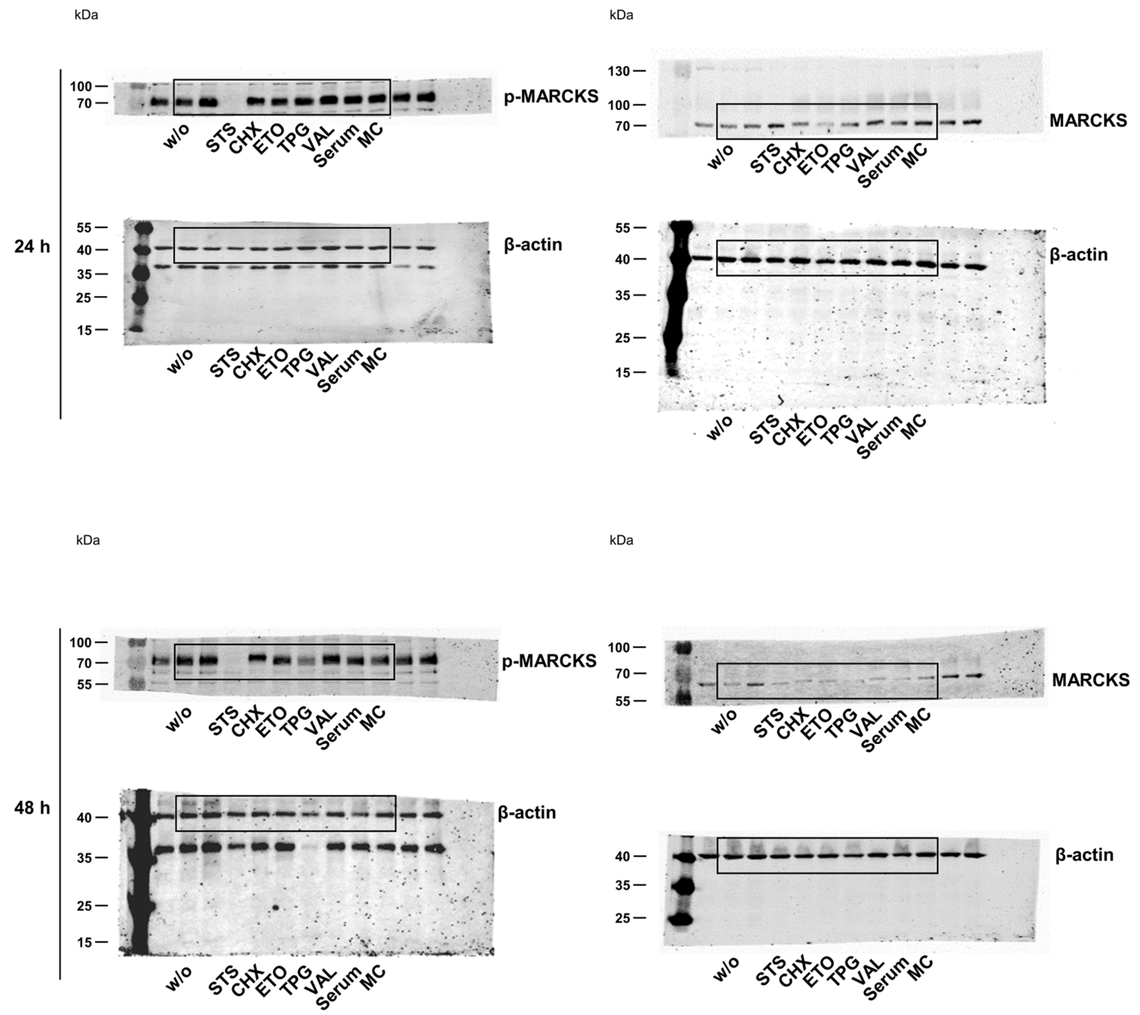

Supplementary Fig. 48 Uncropped versions of the Western Blots (p-MARCKS and MARCKS) presented in Supplementary Fig. 23. The blots for the loading control β-actin shown for p-MARCKS at 24 h and 48 h are identical to Supplementary Fig. 23 (mTOR) and Supplementary Fig. 44.

Supplementary Fig. 23

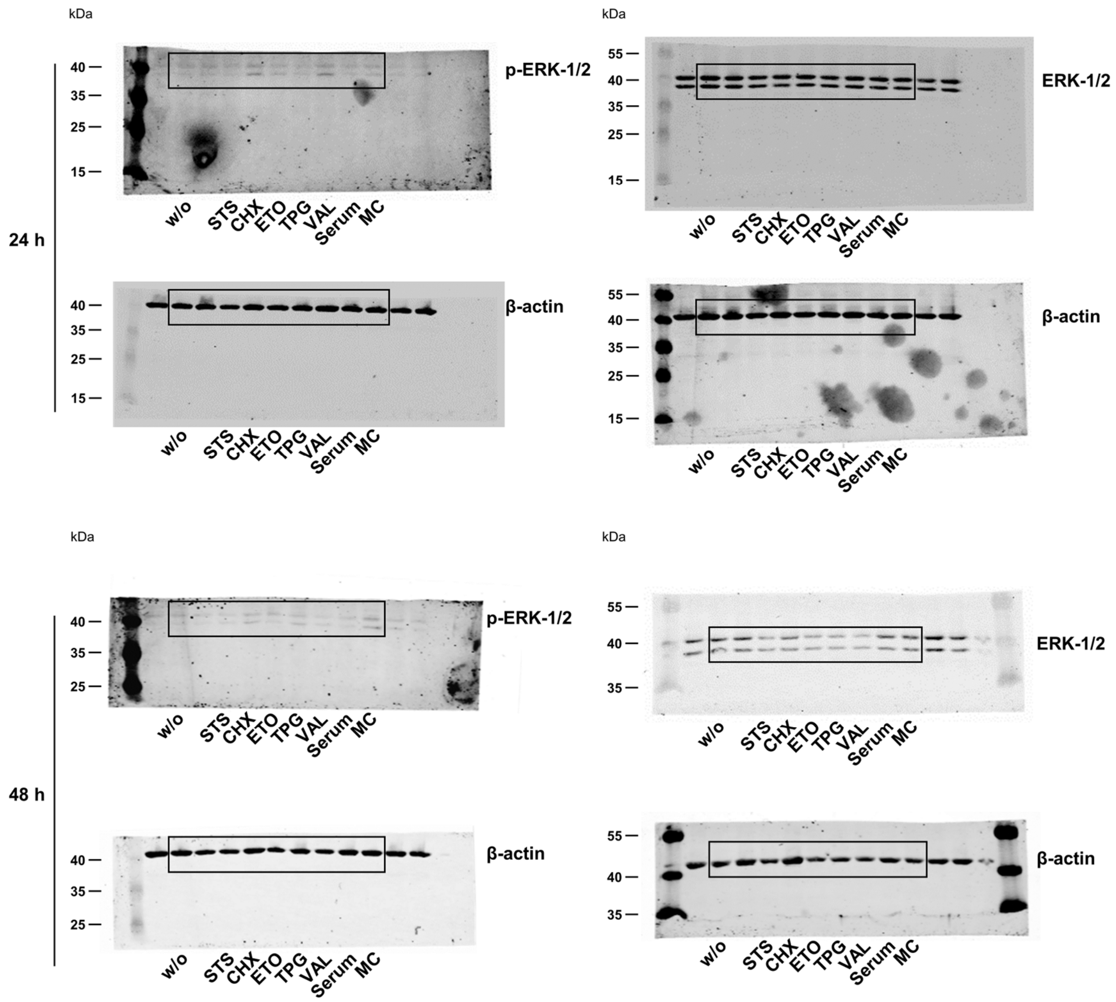

Supplementary Fig. 49 Uncropped versions of the Western Blots (p-ERK-1/2 and ERK-1/2) presented in Supplementary Fig. 23. The blot for the loading control β-actin shown for ERK-1/2 at 24 h is identical to Supplementary Fig. 2a and Supplementary Fig. 38

Supplementary Fig. 24a

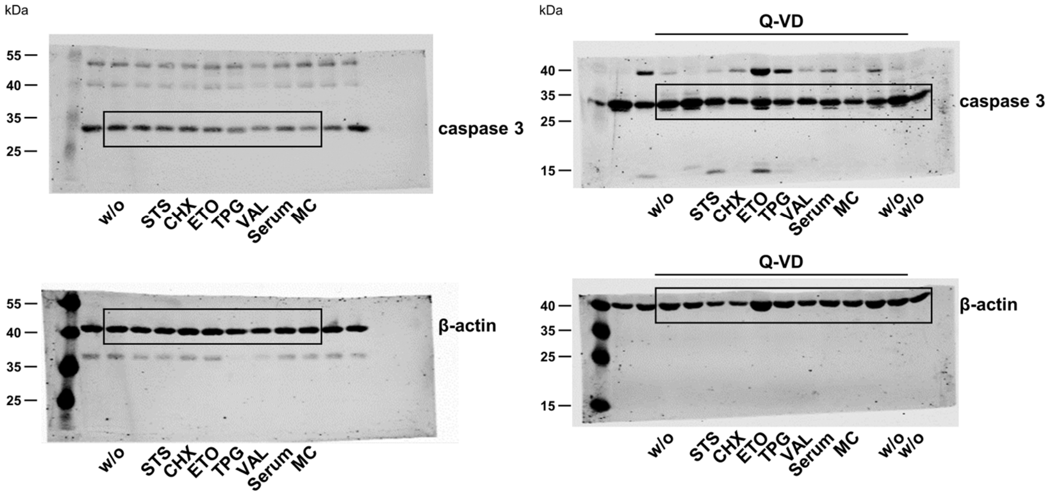

Supplementary Fig. 24b

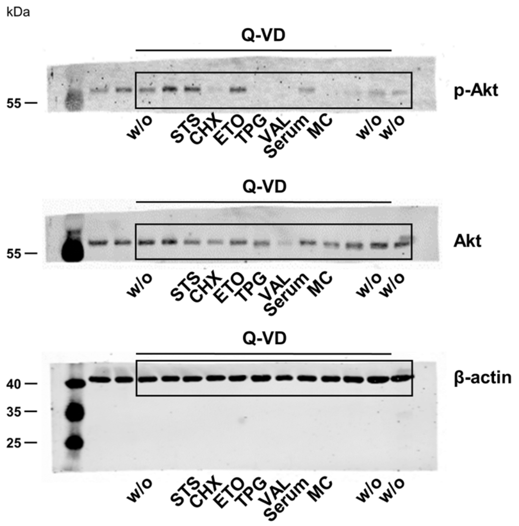

Supplementary Fig. 50 Uncropped versions of the Western Blots presented in Supplementary Fig. 24a and b.

Supplementary Fig. 26a

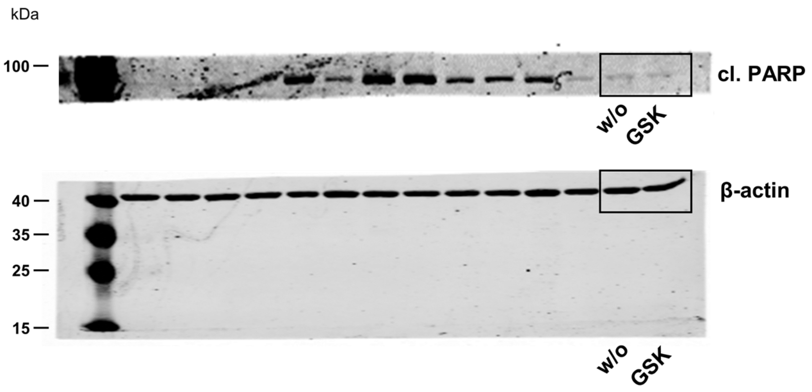

Supplementary Fig. 26b

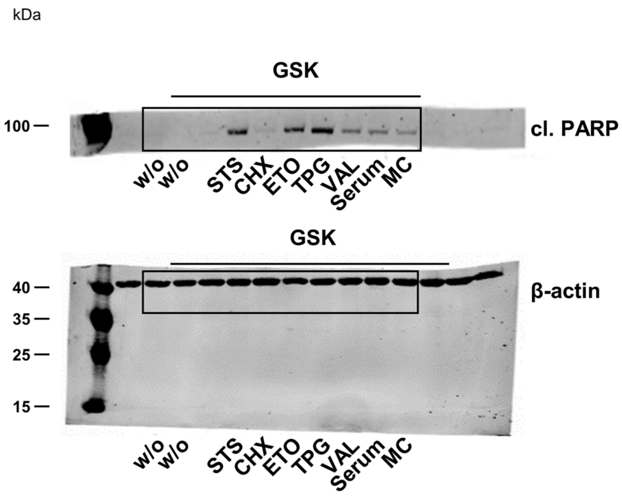

Supplementary Fig. 51 Uncropped versions of the Western Blots presented in Supplementary Fig. 26a and b.

Supplementary Fig. 28

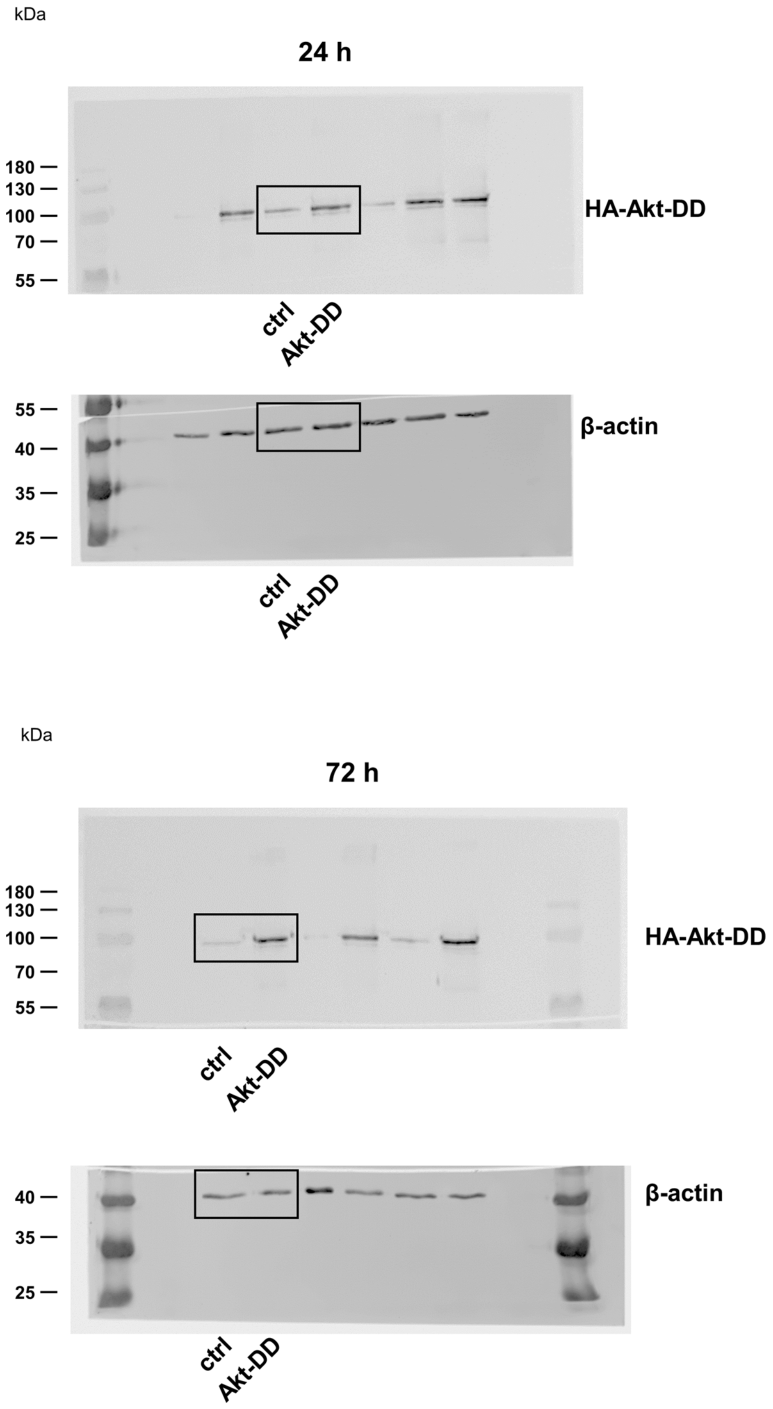

Supplementary Fig. 52 Uncropped versions of the Western Blots presented in Supplementary Fig. 28.

Supplementary Fig. 31

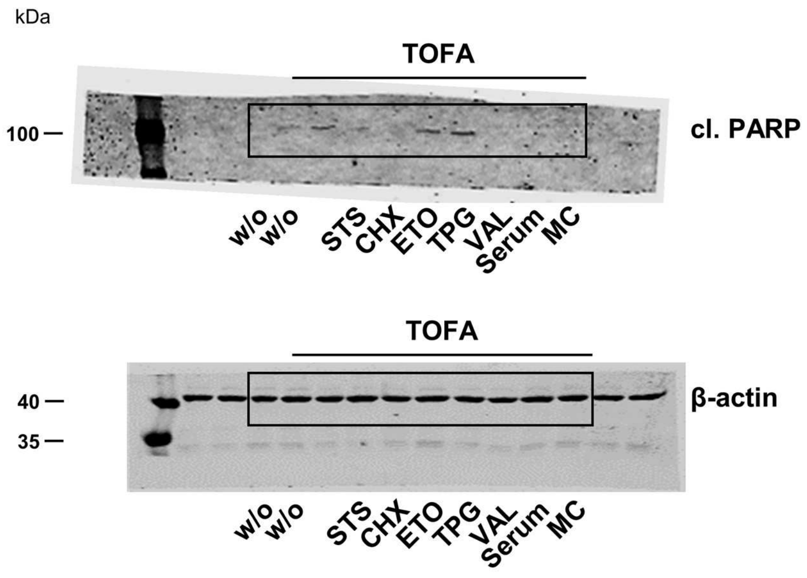

Supplementary Fig. 53 Uncropped versions of the Western Blots presented in Supplementary Fig. 31.

Gating Strategy

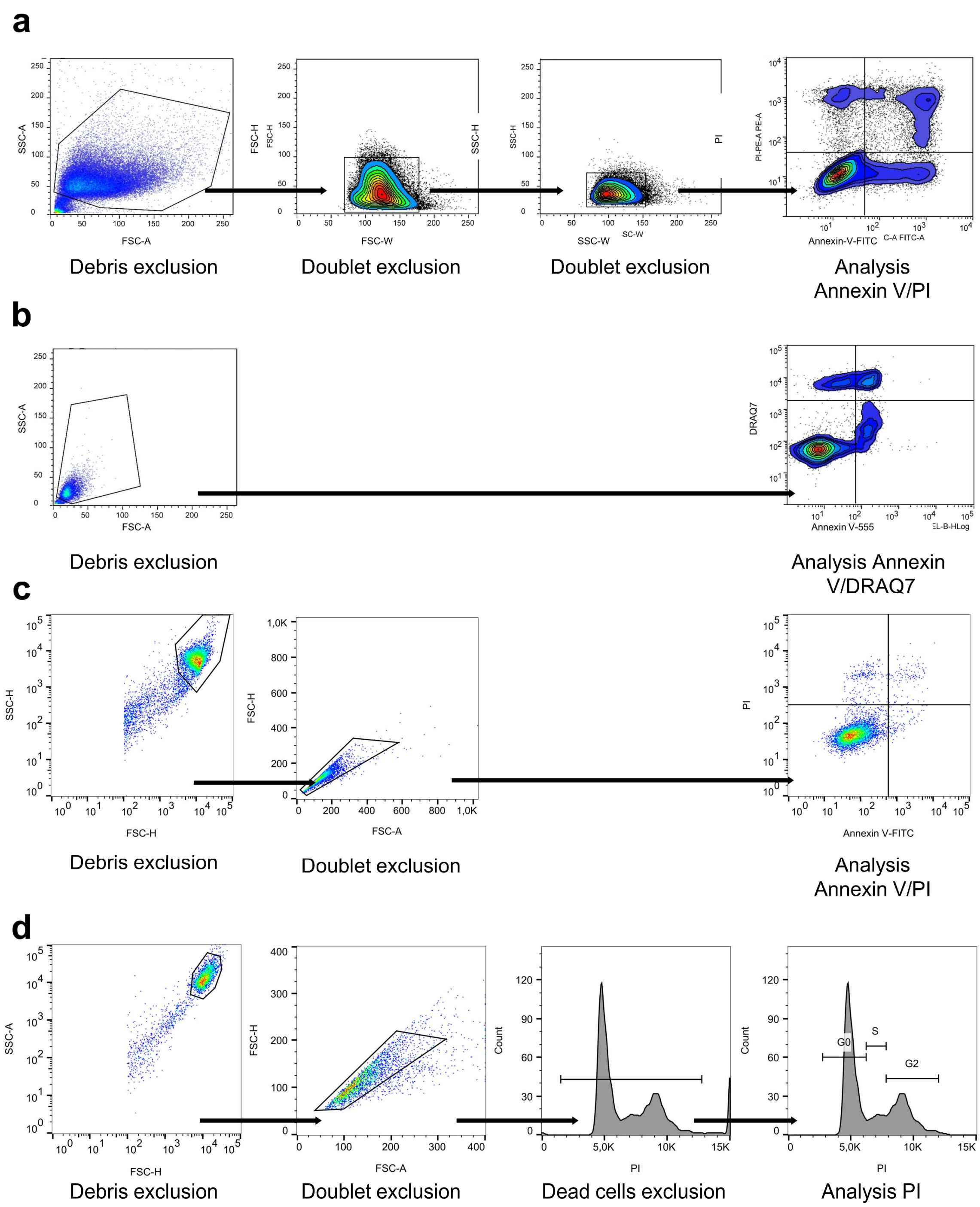

Supplementary Fig. 54 Gating strategies. **a** Supplementary Fig. 2b, c, 26c, d, and 27a, b. **b** Supplementary Fig. 28b, c and 29. **c** Supplementary Fig. 2d, e. **d** Supplementary Fig. 9c.

Supplementary Tables

Supplementary Table 1 Oxidized 20:4 fragment ions selected in Q3

	**Q3 (*m/z*)**	**ion**
+1[O]	319	$[PUFA+O-H]^-$
	317	$[PUFA+O-3H]^-$
	301	$[PUFA+O-H_2O-H]^-$
+2[O]	335	$[PUFA+2O-H]^-$
	331	$[PUFA+2O-5H]^-$
	317	$[PUFA+2O-H_2O-H]^-$

Supplementary Table 2 Retention times and MRM transitions for oxidized PE quantitation

species	retention time ranges [min]	Q1 (*m/z*)	Q3 (*m/z*)
PE(18:0/20:4+1[O])	4.33 – 5.00	782.5	319
PE(16:0/20:4+2[O])	3.65 – 4.05	770.5	331
	2.91 – 3.10	798.5	331
PE(18:0/20:4+2[O])	3.74 – 4.05	798.5	331
	4.13 – 4.40	798.5	331

Supplementary Notes

Supplementary Note 1

Induction of programmed cell death in fibroblasts by multiple pathways

NIH-3T3 fibroblasts were treated with VAL, MC, STS, CHX, ETO, or TPG or deprived of nutrients and growth factors by serum depletion to induce cell death. Cultivation of NIH-3T3 fibroblasts under these mechanistically distinct cytotoxic conditions resulted in a decrease in cell number relative to vehicle-treated cells within 24 to 48 h and induced morphological changes (**Supplementary Fig. 1b**, **c**). Effects on cell morphology were dominated by reduced cell spreading and varied widely between apoptosis inducers (**Supplementary Fig. 1b**). Moreover, cells were fragmented by STS (**Supplementary Fig. 1b**), along with an increase in particle count at 6 h (**Supplementary Fig. 1c**). Cellular dehydrogenase activity, a measure of metabolic activity and cell viability, was substantially reduced at 48 h for all cytotoxic conditions investigated (**Supplementary Fig. 1d**).

Apoptotic progression, as monitored by poly (ADP-ribose) polymerase (PARP) cleavage, started at 24 to 48 h and was most pronounced for ETO and TPG (**Supplementary Fig. 2a**). Externalization of PS, as determined by annexin-V staining and flow cytometry (**Supplementary Fig. 2b**, **c**), was observed for ETO > MC, serum depletion, STS > TPG, VAL, CHX. The cytotoxic stressors at the selected concentrations initially induced mild cell death after 48 h of treatment, which became more severe at higher concentrations, as shown for VAL (**Supplementary Fig. 2d, e**).

Membrane intactness, as determined by trypan blue exclusion, decreased slightly and was significant for VAL and TPG (**Supplementary Fig. 2f**). The induction of apoptosis and secondary necrosis (annexin-V-positive cells) was accompanied by a moderate increase in annexin-V-negative/propidium iodide-positive necrotic cells for MC, VAL, ETO, TPG, and

STS (**Supplementary Fig. 2b**, **c**). Together, VAL, MC, STS, CHX, ETO, TPG, and serum deprivation induce programmed cell death in fibroblasts with different efficiencies, kinetics, and characteristics.

Supplementary Note 2

Cytotoxic stress increases the PUFA-PC ratio independently of caspases

Using the pan-caspase inhibitor Q-VD-OPh[3], we investigated whether caspase activation is required to suppress Akt activation and increase the PUFA-PC ratio. First, we confirmed that Q-VD-OPh inhibits the cleavage and thus activation of caspase-3 under basal and cytotoxic conditions (**Supplementary Fig. 24a**). Next, we determined the effect of Q-VD-OPh on p-Akt depletion (**Fig. 24b**) and PUFA-PC upregulation (**Fig. 24c**) during cell death. Q-VD-OPh neither prevented Akt activation nor the increase in PUFA-PC proportion, and also the decrease in total PC was reduced only in VAL-treated cells (**Fig. 24d**). Thus, our data suggest that caspase-independent pathways essentially shape phospholipid metabolism during the initiation of cell death.

Supplementary Note 3

Akt impairs apoptotic progression in fibroblasts

Inhibition of Akt with GSK690693 per se did not increase apoptotic PARP cleavage (**Supplementary Fig. 26a**), PS externalization, or the number of non-viable cells within 48 to 72 h (**Supplementary Fig. 26c**). However, programmed cell death was enhanced in fibroblasts additionally challenged with VAL from 48 h (**Supplementary Fig. 26c**, **d**). In particular, PARP cleavage (**Supplementary Fig. 26b**) and the fraction of early apoptotic cells and, to a lesser extent, necrotic and late apoptotic cells (**Supplementary Fig. 26c**, **d**) increased under these conditions.

Supplementary Note 4

Cytotoxic regulation of the redox proteome

Val- and MC-treated fibroblasts highly express ERO1a (**Fig. 9c-e** and **Supplementary Fig. 36a**), an ER-localized oxidoreductase that re-oxidizes protein disulfide isomerases[4]. Ero1a is induced by the unfolded protein response (UPR) and burdens cells with hydrogen peroxide[4]. Note that VAL and MC initiate the UPR under our experimental conditions[2]. SCD1 inhibition caused a similar but less pronounced increase in ERO1a expression (**Fig. 9e** and **Supplementary Fig. 36a**, **b**), whereas serum depletion and 18:1 supplementation failed to elevate ERO1A levels (**Fig. 9e** and **Supplementary Fig. 36a**), suggesting that the regulation of ERO1A is independent of growth factor signaling and fatty acid metabolism.

In addition, cell death induction by VAL and MC reduced the availability of multiple proteins that protect against oxidative damage, either by eliminating ROS (SOD2), detoxifying (organic) peroxides, aldehydes, and epoxides (PRDX2, PRDX5, AKR7A2, EPHX1), or regenerating cellular antioxidants (GSR) (**Fig. 9c**, **d**, **g** and **Supplementary Fig. 36a**). The subsequent antioxidant response resulted in increased expression of nuclear factor erythroid-derived 2-like 2 (NRF2) target genes (HMOX1[5], ALDH3A1[6,7], PRDX1[8], GCLM[9], GSTA4[9], CTH[10] (**Fig. 9c**, **d**, **f**, **Supplementary Fig. 18** and **36a**). HMOX1 is a double-edged sword in redox homeostasis and, when highly upregulated, rather promotes ferroptotic cell death by mobilizing Fenton-active heme iron[11].

These (mal)adaptive regulations of the redox proteome were mimicked by SCD1 inhibition, with only few exceptions (HMOX1, ALDH3A1), and partially compensated by either 18:1 (GSR, HMOX1, CTH, GCLM, GSTA4), its metabolite PI(18:1/18:1) (PRDX5), or both of them (SOD2, PRDX1, EPHX1) (**Fig. 9c**, **d**, **f**, **Supplementary Fig. 36a-d**). Serum restriction influences the redox proteome differently than VAL and MC, but also causes an imbalance

(**Supplementary Fig. 36a**). In particular, the cellular levels of the glutamate-cysteine ligase modifier subunit (GCLM) decreased (**Fig. 9h** and **Supplementary Fig. 36a**), which is an integral part of the rate-limiting enzyme in glutathione biosynthesis glutamate-cysteine ligase (GCL). The decrease of GCLM is counteracted by the upregulation of protective enzymes against oxidative stress (IDH1, GSTP1, AKR1B1, GSR, EPHX1, PRDX3, GCLC) (**Supplementary Data 1** and **Supplementary Fig. 36a**).

Suppression of the mevalonate pathway seems to be another common mechanism by which cytotoxic stress increases the sensitivity to membrane peroxidation. In fact, 3-hydroxy-3-methylglutaryl-CoA synthase 1 (HMGCS1), mevalonate kinase (MVK), phosphomevalonate kinase (PMVK), and mevalonate diphosphate decarboxylase (MVD) were markedly repressed in VAL- and MC-treated cells (**Fig. 4a**, **b**, **Supplementary Fig. 19a** and **37**), likely affecting not only cholesterol biosynthesis but also the formation of other isoprenoids, including the radical ion traps ubiquinol and 7-dehydrocholesterol, which protect against ferroptosis[12,13]. In contrast, serum depletion upregulated enzymes involved in the mevalonate pathway (**Supplementary Fig. 19a**), as expected from the higher demand for de novo biosynthesized cholesterol.

Supplementary Note 5

Individual mechanisms of specific cytotoxic stressors

In this study, we investigated diverse cytotoxic agents covering a broad mechanistic range. Because of this heterogeneity and the complex nature of cell death, we observed many compound-specific effects, whose mechanisms have not been further characterized. Here, we did not pursue such compound-specific effects, but focused on mechanisms that might be of general relevance to cell death because they are shared by multiple cytotoxic inducers with different molecular targets.

Many of the cytotoxic conditions applied in this study have been previously reported to modulate lipid metabolism or the PI3K/Akt cascade. For example, TPG irreversibly traps the sarco/endoplasmic reticulum Ca^{2+}-ATPase (SERCA) pump in the Ca^{2+}-free state, thereby depleting intracellular Ca^{2+} stores[14]. The increase in cytoplasmic Ca^{2+} modulates fatty acid biosynthesis, β-oxidation, and phospholipid profiles through multiple pathways, among others, as a cofactor of phospholipases[15], PKC[16], and calmodulin[17]. In summary, a large body of literature describes effects of cytotoxic agents (including TPG) on lipid metabolism and survival signaling, but the underlying mechanisms are often obscure and causal relationships have not been established. By linking RTK/PI3K/Akt survival signaling to fatty acid metabolism and membrane homeostasis, we shed light on the mechanisms by which cytotoxic stress promotes membrane peroxidation and engages multiple cell death programs.

Supplementary Note 6

Putative relevance to phenotypes of ACC-deficient mice

Inhibition of de novo fatty acid biosynthesis by interference with ACC1 or SCD1[2] depletes phospholipids of SFAs and/or MUFAs while relatively accumulating multiple 20:4-, 22:5-, and 22:6-containing species. It is tempting to speculate that a shift in the phospholipid fatty acid composition towards PUFA-containing species contributes to the embryogenic lethality of homozygous *Acc1* knockout mice[18] by increasing the susceptibility to oxidative membrane damage under cell stress. Along these lines, inhibition of fatty acid and phospholipid biosynthesis is well-established to induce cell death[19], and inhibitors of fatty acid biosynthesis have been developed as potential anti-cancer drugs[20,21]. *Acc2*-deficient mice are instead viable, have a higher rate of fatty acid oxidation[22] and show improved insulin sensitivity[23,24] along with increased hepatic Akt phosphorylation[24]. Although our proteomics study did not detect ACC2 in fibroblasts, the very efficient decrease in cellular malonyl-CoA levels suggests that both the cytosolic (ACC1) and mitochondrial isoenzyme (ACC2) are depleted during initiation of cell death, with the latter likely contributing to the initial increase in β-oxidation rate[25].

Supplementary Note 7

Multiple cytotoxic mechanisms suppress fatty acid biosynthesis

Cell death induces a caspase-independent switch in lipid metabolism from de novo fatty acid biosynthesis to β-oxidation. The mechanisms by which fatty acid biosynthesis is decreased vary among cytotoxic stressors and range from transcriptional/post-translational regulation of fatty acid metabolism and substrate limitation to energy depletion through interference with glycolysis, the tricarboxylic acid cycle, and oxidative phosphorylation. Many cell death inducers act through more than one of these mechanisms. The decrease in acetyl-CoA levels might also impair histone acetylation, with potential consequences for the epigenetic regulation of lipogenic enzymes and other factors[26]. Different cell death conditions consequently converge in elevating the cellular proportion of PUFAs in phospholipids throughout intracellular membranes, while decreasing the overall phospholipid content. Serum deprivation engages similar mechanisms, reducing RTK signaling and SFA and MUFA biosynthesis, while maintaining or even increasing the capacity for PUFA membrane incorporation. The subsequent increase in the proportion of PUFA-containing phospholipids was nevertheless surprising, because exogenous PUFAs (other than those released by the cells) are not present in the serum-depleted culture medium. We hypothesize that autophagy, as confirmed under our experimental conditions for serum starvation[2], becomes a major source for fatty acids. Released PUFAs seem to be channeled preferentially into phospholipids over other fatty acids, as expected from the PUFA-specific acyl-CoA transferase ACSL4[27], which becomes dominant during serum deprivation.

Supplementary Note 8

Cytotoxic stress impacts the CL profile

CLs accumulate under cytotoxic stress (**Supplementary Fig. 3a)**, as does the proportion of saturated and unsaturated fatty acids in CLs (**Supplementary Fig. 7e**), and the fatty acid composition of individual species is also substantially altered (**Supplementary Fig. 5b**). As major components of the inner mitochondrial membrane, CLs are oxidized and externalized early in apoptosis, resulting in the release of pro-apoptotic factors that execute apoptosis[28,29]. In addition, CLs have been described to move to other cellular membranes during apoptosis, including the plasma membrane[30,31], a process that might be influenced by the fatty acid composition. Cell death-related changes of the mitochondrial lipid composition might also affect Akt activation, considering that the mitochondrial translocation of the kinase contributes to its anti-apoptotic activity[32].

Supplementary Note 9

Experimental setup for FLIM

The setup used for two photon FLIM has been previously described in detail[33,34]. Briefly, the laser system used for illumination consists of a high power ultrafast Ti:Sapphire laser (Mira HP, Coherent), which is pumped by a frequency doubled continuous wave Nd-Vanadate laser operating at 532 nm (Verdi V18, Coherent). The Ti:Sapphire laser emits 2 ps pulses at 76 MHz pulse repetition rate and approximately 830 nm central wavelength. Since the fluorescence emission is isotropic, it is collected by the water immersion objective (LD C-Apochromat, NA = 1.1, Zeiss) and directed to the FLIM detection module (Becker & Hickl) by a dichroic mirror (long pass 600 nm) and cleaned from residual laser light using a short pass filter (650 nm, Semrock) and a bandpass filter fitting the NAD(P)H emission window (426-490 nm, Semrock). The TCSPC technique has been employed for detecting the arrival time of the fluorescence photons with up to sub-ps time resolution for each pixel of the image. In TCSPC, the number of fluorescence photons and the arrival time after the excitation pulse are registered by a hybrid detector (HPM100, Becker & Hickl) in combination with a fast AD-conversion card (SPC-150, Becker & Hickl). The temporal detection window is determined by the laser pulse repetition rate to 13 ns. The resulting photon distribution can be fitted with up to 3 exponential functions in order to determine amplitude and decay time, which enables to i) identify the fluorophore ii) determine the spatial localization of the fluorophore, iii) investigate differences in the chemical environments of individual fluorophore molecules, and iv) analyze differences in the cell's metabolic states. To ensure sample integrity during the measurements, the laser power incident onto the sample has been reduced to avoid photo-induced sample degradation. Specifically, the laser power was adjusted such that during the exposure time of the FLIM measurement neither an increase in count rate due to the formation of fluorescent species by

phototoxic effects nor a significant decrease in signal level due to photobleaching was observed. The FLIM acquisition was stopped when more than 1000 photons were collected in the brightest pixel. This photon count enables accurate fitting using a bi-exponential decay function.

For data analysis, SPCimage (version 5.3, Becker & Hickl) has been used. The threshold parameter has been adjusted to fit only the image area occupied by fibroblasts to exclude background pixels. Typically, the binning parameter was set to 2 in order to increase the number of photons per decay trace and improve the fitting accuracy. This results in binning 5x5 pixels for analysis and increases the number of photons by a factor of up to 25. The threshold parameter is used to fit only pixels with sufficient photon counts and was set to 5 to 8. The threshold parameter is the maximum number of photons in a single time bin per decay trace after binning.

Supplementary References

1 Espada, L. *et al.* Loss of metabolic plasticity underlies metformin toxicity in aged Caenorhabditis elegans. *Nat Metab* **2**, 1316-1331 (2020).

2 Thürmer, M. *et al.* PI(18:1/18:1) is a SCD1-derived lipokine that limits stress signaling. *Nat Commun* **13**, 2982 (2022).

3 Caserta, T. M., Smith, A. N., Gultice, A. D., Reedy, M. A. & Brown, T. L. Q-VD-OPh, a broad spectrum caspase inhibitor with potent antiapoptotic properties. *Apoptosis* **8**, 345-352 (2003).

4 Zito, E. ERO1: A protein disulfide oxidase and H2O2 producer. *Free Radic Biol Med* **83**, 299-304 (2015).

5 Cuadrado, A. *et al.* Therapeutic targeting of the NRF2 and KEAP1 partnership in chronic diseases. *Nat Rev Drug Discov* **18**, 295-317 (2019).

6 Duong, H. Q., You, K. S., Oh, S., Kwak, S. J. & Seong, Y. S. Silencing of NRF2 Reduces the Expression of ALDH1A1 and ALDH3A1 and Sensitizes to 5-FU in Pancreatic Cancer Cells. *Antioxidants (Basel)* **6** (2017).

7 Matsumoto, R. *et al.* Nuclear Factor Erythroid 2-Related Factor 2 Depletion Sensitizes Pancreatic Cancer Cells to Gemcitabine via Aldehyde Dehydrogenase 3a1 Repression. *J Pharmacol Exp Ther* **379**, 33-40 (2021).

8 Kim, Y. J. *et al.* Human prx1 gene is a target of Nrf2 and is up-regulated by hypoxia/reoxygenation: implication to tumor biology. *Cancer Res* **67**, 546-554 (2007).

9 Lu, S. C. Regulation of glutathione synthesis. *Mol Aspects Med* **30**, 42-59 (2009).

10 Zhao, K., Li, H., Li, S. & Yang, G. Regulation of cystathionine gamma-lyase/H(2)S system and its pathological implication. *Front Biosci (Landmark Ed)* **19**, 1355-1369 (2014).

11 Chiang, S. K., Chen, S. E. & Chang, L. C. A Dual Role of Heme Oxygenase-1 in Cancer Cells. *Int J Mol Sci* **20** (2018).

12 Stockwell, B. R. *et al.* Ferroptosis: A Regulated Cell Death Nexus Linking Metabolism, Redox Biology, and Disease. *Cell* **171**, 273-285 (2017).

13 Freitas, F. P. *et al.* 7-Dehydrocholesterol is an endogenous suppressor of ferroptosis. *Nature* **626**, 401-410 (2024).

14 Clapham, D. E. Calcium signaling. *Cell* **80**, 259-268 (1995).

15 Ghosh, M., Tucker, D. E., Burchett, S. A. & Leslie, C. C. Properties of the Group IV phospholipase A2 family. *Prog Lipid Res* **45**, 487-510 (2006).

16 Spitaler, M. & Cantrell, D. A. Protein kinase C and beyond. *Nature immunology* **5**, 785-790 (2004).

17 Means, A. R. & Dedman, J. R. Calmodulin--an intracellular calcium receptor. *Nature* **285**, 73-77 (1980).

18 Abu-Elheiga, L. *et al.* Mutant mice lacking acetyl-CoA carboxylase 1 are embryonically lethal. *Proc Natl Acad Sci U S A* **102**, 12011-12016 (2005).

19 Currie, E., Schulze, A., Zechner, R., Walther, T. C. & Farese, R. V., Jr. Cellular fatty acid metabolism and cancer. *Cell Metab* **18**, 153-161 (2013).

20 Kuhajda, F. P. Fatty acid synthase and cancer: new application of an old pathway. *Cancer Res* **66**, 5977-5980 (2006).

21 Svensson, R. U. *et al.* Inhibition of acetyl-CoA carboxylase suppresses fatty acid synthesis and tumor growth of non-small-cell lung cancer in preclinical models. *Nat Med* **22**, 1108-1119 (2016).

22 Abu-Elheiga, L., Matzuk, M. M., Abo-Hashema, K. A. & Wakil, S. J. Continuous fatty acid oxidation and reduced fat storage in mice lacking acetyl-CoA carboxylase 2. *Science* **291**, 2613-2616 (2001).

23 Choi, C. S. *et al.* Continuous fat oxidation in acetyl-CoA carboxylase 2 knockout mice increases total energy expenditure, reduces fat mass, and improves insulin sensitivity. *Proc Natl Acad Sci U S A* **104**, 16480-16485 (2007).

24 Abu-Elheiga, L., Wu, H., Gu, Z., Bressler, R. & Wakil, S. J. Acetyl-CoA carboxylase 2-/- mutant mice are protected against fatty liver under high-fat, high-carbohydrate dietary and de novo lipogenic conditions. *J Biol Chem* **287**, 12578-12588 (2012).

25 Saggerson, D. Malonyl-CoA, a key signaling molecule in mammalian cells. *Annu Rev Nutr* **28**, 253-272 (2008).

26 McDonnell, E. *et al.* Lipids Reprogram Metabolism to Become a Major Carbon Source for Histone Acetylation. *Cell Rep* **17**, 1463-1472 (2016).

27 Grevengoed, T. J., Klett, E. L. & Coleman, R. A. Acyl-CoA metabolism and partitioning. *Annu Rev Nutr* **34**, 1-30 (2014).

28 Kagan, V. E. *et al.* Cytochrome c acts as a cardiolipin oxygenase required for release of proapoptotic factors. *Nat Chem Biol* **1**, 223-232 (2005).

29 Kagan, V. E., Chu, C. T., Tyurina, Y. Y., Cheikhi, A. & Bayir, H. Cardiolipin asymmetry, oxidation and signaling. *Chem Phys Lipids* **179**, 64-69 (2014).

30 Manganelli, V. *et al.* Altered Traffic of Cardiolipin during Apoptosis: Exposure on the Cell Surface as a Trigger for "Antiphospholipid Antibodies". *J Immunol Res* **2015**, 847985 (2015).

31 Sorice, M. *et al.* Cardiolipin and its metabolites move from mitochondria to other cellular membranes during death receptor-mediated apoptosis. *Cell Death Differ* **11**, 1133-1145 (2004).

32 Song, H. P., Chu, Z. G., Zhang, D. X., Dang, Y. M. & Zhang, Q. PI3K-AKT Pathway Protects Cardiomyocytes Against Hypoxia-Induced Apoptosis by MitoKATP-Mediated Mitochondrial Translocation of pAKT. *Cell Physiol Biochem* **49**, 717-727 (2018).

33 Heuke, S. *et al.* Detection and Discrimination of Non-Melanoma Skin Cancer by Multimodal Imaging. *Healthcare (Basel)* **1**, 64-83 (2013).

34 Heuke, S. *et al.* Multimodal mapping of human skin. *Br. J. Dermatol.* **169**, 794-803 (2013).
