# Supplementary material for: Attenuated growth factor signaling during cell death initiation sensitizes membranes towards peroxidation
Source: Nat Commun. 2025 Feb 25;16:1774. doi: 10.1038/s41467-025-56711-2 (PMC11861335; doi:10.1038/s41467-025-56711-2)
Supplement: Supplementary file 2 — Description of Additional Supplementary Files [file 41467_2025_56711_MOESM2_ESM.pdf]

## Description of Additional Supplementary Files

**File name: Supplementary Data 1**
Description: Processed proteomics data. Data exported and processed from quantitative proteomics analysis of NIH3T3 fibroblasts exposed to cytotoxic conditions, treated with an SCD1 inhibitor, and/or supplemented with lipids.

**File name: Supplementary Data 2**
Description: Proteomic analysis of pathways investigated. List of proteins considered for the analysis of glycolysis, gluconeogenesis, the tricarboxylic acid cycle, fatty acid biosynthesis, uptake and degradation, (phospho)lipid metabolism, peroxisome biogenesis and metabolism, insulin and growth factor (IGF1, PDGF, and FGF) signaling, and redox homeostasis (related to ROS regulation, antioxidant function, glutathione metabolism, iron metabolism, Nrf2 signaling, or ferroptosis).

**File name: Supplementary Data 3**
Description: Ranking of Gene Ontology terms based on proteomics data. List of Gene Ontology (GO) terms with adjusted P values based on the proteomics datasets for VAL- and MC-treated fibroblasts. The top 100 enriched processes were ranked, assigned scores reflecting the sum of the two individual rankings, and compared to all other treatment groups studied, separately for up- and downregulated biological processes.

**File name: Supplementary Data 4**
Description: Exact P-values from statistical comparisons in Supplementary Figures. List of exact P-values with 15 digits for those panels with at least one P value greater than 0.9999 or less than 0.0001, or for which exact P values are not reported in the Supplementary Information.
