# Supplementary material for: Attenuated growth factor signaling during cell death initiation sensitizes membranes towards peroxidation
Source: Nat Commun. 2025 Feb 25;16:1774. doi: 10.1038/s41467-025-56711-2 (PMC11861335; doi:10.1038/s41467-025-56711-2)
Supplement: Supplementary file 7 — Reporting Summary [file 41467_2025_56711_MOESM7_ESM.pdf]

nature portfolio

Corresponding author(s): Andreas Koeberle

Last updated by author(s): Jan 11, 2025

Reporting Summary

Nature Portfolio wishes to improve the reproducibility of the work that we publish. This form provides structure for consistency and transparency in reporting. For further information on Nature Portfolio policies, see our Editorial Policies and the Editorial Policy Checklist.

Statistics

For all statistical analyses, confirm that the following items are present in the figure legend, table legend, main text, or Methods section.

n/a	Confirmed	
☐	☒	The exact sample size (*n*) for each experimental group/condition, given as a discrete number and unit of measurement
☐	☒	A statement on whether measurements were taken from distinct samples or whether the same sample was measured repeatedly
☐	☒	The statistical test(s) used AND whether they are one- or two-sided<br>*Only common tests should be described solely by name; describe more complex techniques in the Methods section.*
☒	☐	A description of all covariates tested
☐	☒	A description of any assumptions or corrections, such as tests of normality and adjustment for multiple comparisons
☐	☒	A full description of the statistical parameters including central tendency (e.g. means) or other basic estimates (e.g. regression coefficient) AND variation (e.g. standard deviation) or associated estimates of uncertainty (e.g. confidence intervals)
☐	☒	For null hypothesis testing, the test statistic (e.g. *F*, *t*, *r*) with confidence intervals, effect sizes, degrees of freedom and *P* value noted<br>*Give P values as exact values whenever suitable.*
☒	☐	For Bayesian analysis, information on the choice of priors and Markov chain Monte Carlo settings
☒	☐	For hierarchical and complex designs, identification of the appropriate level for tests and full reporting of outcomes
☐	☒	Estimates of effect sizes (e.g. Cohen's *d*, Pearson's *r*), indicating how they were calculated

Our web collection on statistics for biologists contains articles on many of the points above.

Software and code

Policy information about availability of computer code

Data collection	Analyst 1.6 and 1.7 (Sciex), AxioVision 4.8 (Zeiss), BD FACSDIVA 8.0.1 (BD Biosciences), Evolution-Capt Edge Software Version 18.06 (Vilber Lourmat), guavaSoft 3.1.1 (Merck Millipore), Mx3005P v4.10 software (Agilent Technologies), NOVOstar software version 1.30 (BMG Labtech), Odyssey Infrared Imaging System Application Software Version 3.0 (LI-COR Biosciences), Vi-Cell XR Cell Viability Analyzer 2.03 and 2.06.3 (Beckman Coulter), Orbitrap Tribrid Series Tune software 3 (Thermo Fisher Scientific), SoftMax Pro 7.1 (Molecular Devices), SPC-150 v. 9.71 64 bit (Becker&Hickl), Wallac 1414 WinSpectral software (Perkin Elmer), BZ-X800 Analyzer and BZ-X800 Viewer (Keyence).
Data analysis	Analyst 1.6 and Analyst 1.7 (Sciex), Bio-1D Version 15.08c (Vilber Lourmat), BD FACSDiva 8.0.1 (BD Biosciences), Cancer Therapeutics Response Portal (https://portals.broadinstitute.org/ctrp.v2.1/; 09/2022), CorrelationCalculator v1.0.1 (Basu, S. et al. Sparse network modeling and metscape-based visualization methods for the analysis of large-scale metabolomics data. Bioinformatics 33, 1545-1553 (2017)), Cytoscape 3.9.1 (Cytoscape Consortium), Fiji (https://imagej.net/software/fiji/), FlowJo ™ v10.10 (BD Biosciences), Flowlogic 7.3 and 8.7 (Miltenyi Biotech, Bergisch Gladbach, Germany), GraphPad Prism 9 and 10 (GraphPad Software), MetScape v3.1.3 App (http://metscape.ncibi.org/) for Cytoscape (Cytoscape Consortium), Microsoft 365 (Microsoft), Odyssey Infrared Imaging System Application Software Version 3.0, OriginPro 2020 (OriginLab), Proteome Discoverer 2.4 (Thermo Fisher Scientific), SigmaPlot 14 (Systat Software GmbH) and SPCimage version 5.3 (Becker&Hickl).

For manuscripts utilizing custom algorithms or software that are central to the research but not yet described in published literature, software must be made available to editors and reviewers. We strongly encourage code deposition in a community repository (e.g. GitHub). See the Nature Portfolio guidelines for submitting code & software for further information.

Data

Policy information about availability of data

All manuscripts must include a data availability statement. This statement should provide the following information, where applicable:

- Accession codes, unique identifiers, or web links for publicly available datasets
- A description of any restrictions on data availability
- For clinical datasets or third party data, please ensure that the statement adheres to our policy

The mass spectrometric lipidomics data generated in this study have been deposited in the Metabolomics Workbench database (an international repository for metabolomics data and metadata, metabolite standards, protocols, tutorials and training, and analysis tools (Sud, M. et al. Metabolomics Workbench: An international repository for metabolomics data and metadata, metabolite standards, protocols, tutorials and training, and analysis tools. Nucleic Acids Res. 44, D463-470 (2016)) under accession codes PR001114 [ST001740; http://dx.doi.org/10.21228/M8Q39V]166 and PR002065 [ST003318, ST003319, ST003320, ST003321, ST003336, ST003337; http://dx.doi.org/10.21228/M8PR7B] or in the repository for research data of the University of Innsbruck powered by InvenioRDM (https://doi.org/10.48323/22gty-xjr51). The mass spectrometric proteomics data have been deposited to the ProteomeXchange Consortium via the PRIDE (Perez-Riverol, Y. et al. PRIDE Inspector Toolsuite: Moving Toward a Universal Visualization Tool for Proteomics Data Standard Formats and Quality Assessment of ProteomeXchange Datasets. Mol Cell Proteomics 15, 305-317 (2016)) partner repository with the dataset identifier PXD025396 [http://www.ebi.ac.uk/pride/archive/projects/PXD025396] and PXD053866 [https://www.ebi.ac.uk/pride/archive/projects/PXD053866]. The processed mass spectrometric data are available at the above-mentioned repositories, the Source Data or the Supplementary Data. The correlation data between gene expression and cellular resistance to small molecules used in this study are available at the Cancer Therapeutics Response Portal (https://portals.broadinstitute.org/ctrp.v2.1/; 09/2022). All other data generated or used in this study are provided in the Source Data, Supplementary Information, or Supplementary Data.

Research involving human participants, their data, or biological material

Policy information about studies with human participants or human data. See also policy information about sex, gender (identity/presentation), and sexual orientation and race, ethnicity and racism.

Reporting on sex and gender	This information is not available to use for the anonymized scientific study on primary human innate immune cells isolated from buffy coats in accordance with the ethical approval.
Reporting on race, ethnicity, or other socially relevant groupings	Regular blood donors at the Institute for Transfusion Medicine of the University Hospital Jena (Germany). More detailed information is not available to us.
Population characteristics	Human leukocyte concentrates were provided by the Institute for Transfusion Medicine of the University Hospital Jena (Germany). These subjects donated blood every 8 to 12 weeks, had no apparent infections, inflammatory conditions, or current allergic reactions (according to prior physical inspection by a clinician) and had not taken antibiotics or anti-inflammatory drugs for at least 10 days prior to blood collection.
Recruitment	Venous blood was collected from fasted (12 h) adult (18–65 years) male and female registered healthy volunteers, with informed consent, by the Institute for Transfusion Medicine of the University Hospital Jena (Germany). Authors of this study were neither involved in the recruitment nor selection of blood donors, who were randomly assigned by the Transfusion Medicine to research requests.
Ethics oversight	Ethical commission of the Friedrich-Schiller-University Jena

Note that full information on the approval of the study protocol must also be provided in the manuscript.

Field-specific reporting

Please select the one below that is the best fit for your research. If you are not sure, read the appropriate sections before making your selection.

☒ Life sciences ☐ Behavioural & social sciences ☐ Ecological, evolutionary & environmental sciences

For a reference copy of the document with all sections, see nature.com/documents/nr-reporting-summary-flat.pdf

Life sciences study design

All studies must disclose on these points even when the disclosure is negative.

Sample size	Sample size was not pre-determined by statistical methods but was estimated based on previous studies. Cell-based studies on the lipid profile, cell metabolism, lipid metabolism, mitogenic signaling, (oxidative) stress response, survival signaling, and cell death were at least performed in three independent biological experiments, as described before (Thürmer et al. PI(18:1/18:1) is a SCD1-derived lipokine that limits stress signaling. Nat Commun 13, 2982 (2022), Koeberle, A. et al. Role of p38 mitogen-activated protein kinase in linking stearoyl-CoA desaturase-1 activity with endoplasmic reticulum homeostasis. FASEB J 29, 2439-2449 (2015), and Bartel, K. et al. Connecting lysosomes and mitochondria - a novel role for lipid metabolism in cancer cell death. Cell Commun Signal 17, 87 (2019)). Investigations related to the lipid profile of C. elegans were based on ≥ 700 animals. The sample size was estimated based on previous experiments (Espada et al. Loss of metabolic plasticity underlies metformin toxicity in aged Caenorhabditis elegans. Nat Metab 2, 1316-1331 (2020)).

Data exclusions	Based on pre-established exclusion criteria, significant outliers (P < 0.05) were excluded from the analyses in Fig. 6a (STS, TPG, VAL, SER) and Fig. 6b (VAL, 24 h) using a Grubb's test (significance level of alpha = 0.05) or in Fig. 7e and Supplementary Fig. 21b using the ROUT method (Q = 0.1 %), but not from other figures. Unequal numbers of data sets in these analyses derive from different designs, samples not complying to our quality criteria, or loss of sample.
Replication	The replication of the data was successful. Data were generated in independent experiments or are representative for multiple animals.<br><br>1) Independent experiments on cultured or isolated cells: n ≥ 3<br>2) Lipidomic analyses of C. elegans are based on n ≥ 700 animals.<br><br>Detailed information about the exact number of experiments is given in the figure legends.
Randomization	Flasks/wells with seeded cells or animals were randomly assigned to treatment groups. Quantitative proteomics sample were run in a randomized order.
Blinding	Samples were not blinded. Rational: a) Readouts were quantitatively assessed. b) Sample preparation directly followed individual cell treatment by the same operator and was subjected to strict time schedules, which was poorly compatible to blinding. c) Experiments have been conducted following established protocols leaving no room for biased analysis. d) The data collection and technical analysis of cell counts, cell viability, lipids, and proteoms has been automatically conducted and manually controlled, which further minimized biased analysis.

Reporting for specific materials, systems and methods

We require information from authors about some types of materials, experimental systems and methods used in many studies. Here, indicate whether each material, system or method listed is relevant to your study. If you are not sure if a list item applies to your research, read the appropriate section before selecting a response.

Materials & experimental systems

n/a	Involved in the study	
☐	☒	Antibodies
☐	☒	Eukaryotic cell lines
☒	☐	Palaeontology and archaeology
☐	☒	Animals and other organisms
☒	☐	Clinical data
☒	☐	Dual use research of concern
☒	☐	Plants

Methods

n/a	Involved in the study	
☒	☐	ChIP-seq
☐	☒	Flow cytometry
☒	☐	MRI-based neuroimaging

Antibodies

Antibodies used

I. Western Blot - Primary Antibodies

Cell Signaling Technology (Danvers, MA):
- rabbit anti-acetyl-CoA carboxylase (1:1000; # 3662)
- mouse anti-Akt (pan; 40D4; 1:1000; # 2920)
- rabbit anti-caspase 3 (1:1000; # 9662)
- mouse anti-cleaved PARP (Asp214; 7C9; 1:1000; # 9548)
- rabbit anti-ERK1/2 (p44/42 MAPK; 137F5; 1:1000; # 4695)
- rabbit anti-fatty acid synthase (1:1000; # 3189)
- rabbit anti-GAPDH (14C10; 1:1000; # 2118)
- mouse anti-GAPDH (D4C6R; 1:1000; # 97166)
- mouse anti-GSK-3β (3D10; 1:1000; # 9832)
- rabbit anti-HA-Tag (C29F4; 1:1000; # 3724)
- mouse anti-IκBα (L35A5; 1:1000; # 4814)
- mouse anti-mTOR (L27D4; 1:1000; # 4517)
- rabbit anti-p70 S6 kinase (49D7; 1:1000; # 2708)
- rabbit anti-phospho-acetyl-CoA carboxylase (Ser79; D7D11; 1:1000; # 11818)
- rabbit anti-phospho-Akt (Ser473; 1:500 - 1:1000; # 9271)
- rabbit anti-phospho-AMPKα (Thr172; 40H9; 1:1000; # 2535)
- mouse anti-phospho-ERK1/2 (p44/42 MAPK; Thr202/Tyr204; E10; 1:1000; # 9106)
- rabbit anti-phospho-GSK-3β (Ser9; D85E12; 1:1000; # 5558)
- rabbit anti-phospho-IκBα (Ser32; 14D4; 1:1000; # 2859)
- rabbit anti-phospho-MARCKS (Ser152/156; 1:1000; # 2741)
- rabbit anti-phospho-mTOR (Ser2448; D9C2; 1:1000; # 5536)
- mouse anti-phospho-p70 S6 kinase (Thr389; 1A5; 1:1000; # 9206)
- rabbit anti-β-actin (13E5; 1:1000; # 4970)
- mouse anti-β-actin (8H10D10; 1:1000; # 3700)
- rabbit anti-β-tubulin (9F3; 1:1000; # 2128)
- rabbit anti-SCD1 (M38; 1:500; #2438)

Abcam (Cambridge, UK):
- rabbit anti-Lamin B1 (1:1000; # ab16048)
- rabbit anti-MARCKS (1:1000; # ab51100)
- mouse anti-plasma membrane Ca2+ ATPase (PMCA; 5F10; 1:1000; # ab2825)
- rabbit anti-SREBP1 (1:1000; #ab28481)

Santa Cruz (Dallas, TX)
- rabbit anti-calnexin (H-70; 1:1000; # sc-11397)

Proteintech (Manchester, UK)
- rabbit anti-ACSL3 (1: 5000; #20710-1-AP)
- rabbit anti-ACSL5 (1: 2000; #15708-1-AP)
- rabbit anti-ACSL4 (1:2500; #22401-1-AP)

ABclonal (Woburn, MA)
- rabbit anti-LPCAT3 (1: 2000; #A17604)

II. Western Blot - Secondary Antibodies
- IRDye 800CW-conjugated anti-rabbit IgG (1:10,000; # 926-32211, LI-COR Biosciences)
- IRDye 800CW-conjugated anti-mouse IgG (1:10,000; # 926-32210, LI-COR Biosciences)
- IRDye 680LT-conjugated anti-rabbit IgG (1:80,000; # 926-68021, LI-COR Biosciences)
- IRDye 680LT-conjugated anti-mouse IgG (1:80,000; # 926-68020, LI-COR Biosciences)
- DyLight® 800 anti-rabbit IgG (1:10,000; # SA510036, Thermo Fisher Scientific)
- DyLight® 680 anti-rabbit IgG (1:10,000, # 35569, Thermo Fisher Scientific)

III. Immunoprecipitation
- anti-pan-Akt (40D4; 1:200; # 2920, Cell Signaling Technology)

IV. FLIM/CARS microscopy
- rabbit anti-p-Akt (Ser472; Cell Signaling, #9271, 1:400)
- goat anti-rabbit IgG Alexa Fluor 488 (Thermo Fisher Scientific, A32731, 1:500)

V. Immunofluorescence microscopy - Primary Antibodies
- mouse anti-4-HNE (12F7) (1:50, # MA5-27570, Lot: ZC4192871; Thermo Fisher Scientific)
- rabbit anti-cleaved caspase-3 (Asp175) (1:400, #9661, Lot: 47; Cell Signaling Technology)
- rabbit anti-SREBP1 (1:100, #ab28481, Lot: 1051053-6; Abcam)

VI. Immunoflluorescence microscopy - Secondary Antibodies
- Alexa Fluor™ 555 goat anti-mouse IgG (1:1000, # A21422, Lot: 10143952; Thermo Fisher Scientific)
- Alexa Fluor™ 488 goat anti-rabbit IgG (1:500, # 11034, Lot: 10729174; Thermo Fisher Scientific)

Validation

All antibodies used in the current study were validated by the manufacturers. Detailed information about the manufacturer's validation process can be found at:

1) Cell Signaling Technology: https://www.cellsignal.com/about-us/cst-antibody-validation-principles
2) Abcam: https://www.abcam.com/primary-antibodies/how-we-validate-our-antibodies
3) Santa Cruz Biotechnology: https://www.scbt.com/about-us
4) LI-COR Biosciences: https://www.licor.com/bio/reagents/irdye-infrared-dyes
5) Thermo Fisher Scientific: https://www.thermofisher.com/at/en/home/life-science/antibodies.html
6) Proteintech https://www.ptglab.com/products/antibodies-and-immunoassays/#Antibodies

The following list gives the species reactivity of each antibody according to manufacturer's information:

I. Western Blot - Primary Antibodies
- rabbit anti-acetyl-CoA carboxylase: Species Reactivity: Human, Mouse, Rat, Monkey, Bovine
- mouse anti-Akt: Species Reactivity: Human, Mouse, Rat, Monkey
- rabbit anti-caspase 3: Species Reactivity: Human, Mouse, Rat, Monkey
- mouse anti-cleaved PARP: Species Reactivity: Mouse
- rabbit anti-ERK1/2: Species Reactivity: Human, Mouse, Rat, Hamster, Monkey, Mink, D. melanogaster, Zebrafish, Bovine, Dog, Pig, C. elegans
- rabbit anti-fatty acid synthase: Species Reactivity: Human, Mouse
- rabbit anti-GAPDH: Species Reactivity: Human, Mouse, Rat, Monkey, Bovine, Pig
- mouse anti-GAPDH: Species Reactivity: Human, Mouse, Rat, Monkey
- mouse anti-GSK-3β: Species Reactivity: Human, Mouse, Rat, Hamster, Monkey
- rabbit anti-HA-Tag: Species Reactivity: All Species Expected
- mouse anti-IκBα: Species Reactivity: Human, Mouse, Rat, Monkey, Bovine, Pig
- mouse anti-mTOR: Species Reactivity: Human, Mouse, Rat, Monkey
- rabbit anti-p70 S6 kinase: Species Reactivity: Human
- rabbit anti-phospho-acetyl-CoA carboxylase: Species Reactivity: Human, Mouse, Rat
- rabbit anti-phospho-Akt: Species Reactivity: Human, Mouse, Rat, Hamster, Monkey, D. melanogaster, Bovine, Dog
- rabbit anti-phospho-AMPKα: Species Reactivity: Chicken, Zebrafish, Bovine, Pig
- mouse anti-phospho-ERK1/2: Species Reactivity: Human, Mouse, Rat, Hamster, Monkey, Mink, Zebrafish, Bovine, Pig
- rabbit anti-phospho-GSK-3β: Species Reactivity: Human, Mouse, Rat, Hamster
- rabbit anti-phospho-IκBα: Species Reactivity: Human, Mouse, Rat, Monkey

- rabbit anti-phospho-MARCKS: Species Reactivity: Human, Mouse, Rat
- rabbit anti-phospho-mTOR: Species Reactivity: Human, Mouse, Rat, Monkey
- mouse anti-phospho-p70 S6 kinase: Species Reactivity: Human, Mouse, Rat, Monkey, D. melanogaster
- rabbit anti-β-actin: Species Reactivity: Human, Mouse, Rat, Monkey, Bovine, Pig
- mouse anti-β-actin: Species Reactivity: Human, Mouse, Rat, Hamster, Monkey, Dog
- rabbit anti-β-tubulin: Species Reactivity: Species Reactivity: Human, Mouse, Rat, Monkey, Zebrafish, Bovine
- rabbit anti-Lamin B1: Species Reactivity: Human, Mouse, Rat
- rabbit anti-MARCKS: Species Reactivity: Human
- mouse anti-plasma membrane Ca2+ ATPase: Species Reactivity: Mouse, Rat, Sheep, Rabbit, Chicken, Hamster, Cow, Cat, Dog, Human, Amphibian, Syrian hamster
- rabbit anti-calnexin: Species Reactivity: Human, Dog, Bovine, Pig
- rabbit anti-SCD1: Species Reactivity: Human, Mouse
- rabbit anti-SREBP1: Species Reactivity: Human, Mouse, Rat
- rabbit anti-ACSL3: Species Reactivity: Human, Mouse
- rabbit anti-ACSL5: Species Reactivity: Human, Mouse, Rat
- rabbit anti-ACSL4: Species Reactivity: Human, Mouse, Rat
- rabbit anti-LPCAT3: Species Reactivity: Human, Mouse, Rat

II. Western Blot - Secondary Antibodies
- IRDye 800CW-conjugated anti-rabbit IgG: Species Reactivity: Rabbit
- IRDye 800CW-conjugated anti-mouse IgG: Species Reactivity: Mouse
- IRDye 680LT-conjugated anti-rabbit IgG: Species Reactivity: Rabbit
- IRDye 680LT-conjugated anti-mouse IgG: Species Reactivity: Mouse
- DyLight® 800 anti-rabbit IgG: Species Reactivity: Rabbit
- DyLight® 680 anti-rabbit IgG: Species Reactivity: Rabbit

III. Immunoprecipitation
- anti-pan-Akt: Species Reactivity: Human, Mouse, Rat, Monkey

IV. FLIM/CARS microscopy
- rabbit anti-p-Akt: Species Reactivity: Human, Mouse, Rat, Hamster, Monkey, D. melanogaster, Bovine, Dog
- goat anti-rabbit IgG Alexa Fluor 488: Species Reactivity: Rabbit

V. Immunofluorescence microscopy - Primary Antibodies
- mouse anti-4-HNE (12F7): Species Reactivity: Chemical
- rabbit anti-cleaved caspase-3: Species Reactivity: Human, Mouse, Rat, Monkey
- rabbit anti-SREBP1: Species Reactivity: Human, Mouse, Rat

VI. Immunoflluorescence microscopy - Secondary Antibodies
- Alexa Fluor™ 555 goat anti-mouse IgG: Species Reactivity: Mouse
- Alexa Fluor™ 488 goat anti-rabbit IgG: Species Reactivity: Rabbit

Eukaryotic cell lines

Policy information about cell lines and Sex and Gender in Research

Cell line source(s)

Cells were from the German Collection of Microorganisms and Cell Cultures (DSMZ, Braunschweig, Germany), the American Type Culture Collection (ATCC, Manassas, VA), or the Japanese Collection of Research Bioresources Cell Bank (JCRB Cell Bank, Ibaraki, Japan)

- mouse NIH-3T3 fibroblasts (# ACC 59, DSMZ)
- mouse Swiss 3T3 fibroblasts (# CCL-92, ATCC)
- mouse 3T3-L1 preadipocytes (#CL-173, ATCC)
- mouse Hepa 1-6 hepatocarcinoma cells (#RCB1638, RIKEN)
- Human HeLa cervical carcinoma cells (# ACC 57, DSMZ)
- human MDA-MB-231 triple-negative breast cancer cells (#HTB-26, ATCC)
- human MDA-MB-231 triple-negative breast cancer cells stably transfected with GFP (a gift from Simone and Thomas Brabletz, University of Erlangen-Nürnberg, Germany)
- human HT-29 colon adenocarcinoma cells (# HTB-38, ATCC)
- human HEK-293 embryonic kidney cells (# CRL-1573, ATCC)
- human A549 lung carcinoma cells (# CCL-185, ATCC)
- human HepG2 hepatocarcinoma cells (# ACC 180, DSMZ)
- Human MCF-7 breast adenocarcinoma cells (# HTB-22, ATCC)
- HepaRG cells (# HPR101, Biopredic International, Rennes, France)
- Human HUH-7 hepatocarcinoma cells (# JCRB0403, JCRB Cell Bank)
- Human MM6 acute monocytic leukemia cells (# ACC 124, DSMZ)

Authentication

MCF-7 and HEK-293 cells were authenticated by Multiplexion (Friedrichshafen, Germany; December, 2020) using Single Nucleotide Polymorphism (SNP) profiling (Multiplex Cell Line Authentication, https://www.multiplexion.de/en/cell-line-testing-service/multiplex-human-cell-line-authentication). Other cell lines were not authenticated. Cell morphology of all cell lines was regularly inspected.

Mycoplasma contamination

Cell lines were tested for mycoplasma and found negative for contamination.

Commonly misidentified lines (See ICLAC register)	HEK-293 was reported to be a misidentified cell line and was used in this study due to high basal p-Akt levels.

Animals and other research organisms

Policy information about studies involving animals; ARRIVE guidelines recommended for reporting animal research, and Sex and Gender in Research

Laboratory animals	C. elegans strain N2 Bristol (Caenorhabditis Genetics Center, University of Minnesota, Minneapolis, MN), young (adulthood day 1, AD1) and old (AD10)
Wild animals	The study did not involve wild animals.
Reporting on sex	Does not apply to C. elegans
Field-collected samples	The study did not involve samples collected from the field.
Ethics oversight	Not applicable to studies on C. elegans.

Note that full information on the approval of the study protocol must also be provided in the manuscript.

Flow Cytometry

Plots

Confirm that:

- [x] The axis labels state the marker and fluorochrome used (e.g. CD4-FITC).
- [x] The axis scales are clearly visible. Include numbers along axes only for bottom left plot of group (a 'group' is an analysis of identical markers).
- [x] All plots are contour plots with outliers or pseudocolor plots.
- [x] A numerical value for number of cells or percentage (with statistics) is provided.

Methodology

Sample preparation	NIH-3T3 fibroblasts were harvested and stained with propidium iodide and annexin-V using an an Annexin V Apoptosis Detection Kit FITC (Thermo Fisher Scientific) according to the manufacturer's instructions. Alternatively, NIH-3T3 cells were stained with DRAQ7 (Deep Red Anthraquinone 7, 564904, BD Biosciences) and Annexin V Alexa Fluor 555 conjugate (# A35108, Thermo Fisher Scientific). For cell cycle analysis, NIH-3T3 cells were harvested, fixed with 70% EtOH, treated with bovine pancreas RNase A and subsequently stained with propidum iodide (HY-D0815, MedChemExpress).
Instrument	BD LSR Fortessa flow cytometer (BD Biosciences); Guava easyCyte 8HT flow cytometer (Merck Millipore)
Software	BD FACSDiva 8.0.1 (BD Biosciences); Flowlogic 7.3 (Miltenyi Biotech, Bergisch Gladbach, Germany); Flowlogic 8.7 (Miltenyi Biotech), FlowJo™ v10.10 (Ashland, OR)
Cell population abundance	Flow cytometric studies were conducted with a cell line (mouse NIH-3T3 cells).
Gating strategy	Cells were pre-gated on FSC/SSC following single cell identification to exclude debris and aggregates. Pre-gated cells were analyzed for Annexin V and PI signals. Alternatively, cells were pre-gated on FSC/SSC and analyzed for Annexin V and DRAQ7 signals. Boundaries between positive and negative fractions were partially defined using single stainings of treated samples. For cell cycle analysis, cells were pre-gated to exclude debris (FSC/SSC), aggregates and dead cells and then analyzed for PI signals.

- [x] Tick this box to confirm that a figure exemplifying the gating strategy is provided in the Supplementary Information.
